# Design, Synthesis and Anticandidal Evaluation of Indazole and Pyrazole Derivatives

**DOI:** 10.3390/ph14030176

**Published:** 2021-02-24

**Authors:** Karen Rodríguez-Villar, Alicia Hernández-Campos, Lilián Yépez-Mulia, Teresita del Rosario Sainz-Espuñes, Olivia Soria-Arteche, Juan Francisco Palacios-Espinosa, Francisco Cortés-Benítez, Martha Leyte-Lugo, Bárbara Varela-Petrissans, Edgar A. Quintana-Salazar, Jaime Pérez-Villanueva

**Affiliations:** 1 Doctorado en Ciencias Biológicas y de la Salud, Universidad Autónoma Metropolitana (UAM), Ciudad de México 04960, Mexico; qkarenrodv@hotmail.com; 2 Departamento de Farmacia, Facultad de Química, Universidad Nacional Autónoma de México (UNAM), Ciudad de México 04510, Mexico; hercam@unam.mx; 3 Unidad de Investigación Médica en Enfermedades Infecciosas y Parasitarias, UMAE Hospital de Pediatría, Centro Médico Siglo XXI, Instituto Mexicano del Seguro Social, Ciudad de México 06720, Mexico; lilianyepez@yahoo.com; 4 Departamento de Sistemas Biológicos, División de Ciencias Biológicas y de la Salud, Universidad Autónoma Metropolitana-Xochimilco (UAM-X), Ciudad de México 04960, Mexico; trsainz@correo.xoc.uam.mx (T.d.R.S.-E.); soriao@correo.xoc.uam.mx (O.S.-A.); jpalacios@correo.xoc.uam.mx (J.F.P.-E.); jcortesb@correo.xoc.uam.mx (F.C.-B.); bvra5302@gmail.com (B.V.-P.); edgarqsl2811@gmail.com (E.A.Q.-S.); 5 Catedrático CONACyT Comisionado al Departamento de Sistemas Biológicos, División de Ciencias Biológicas y de la Salud, Universidad Autónoma Metropolitana-Xochimilco (UAM-X), Ciudad de México 04960, Mexico; mleyte@correo.xoc.uam.mx

**Keywords:** anticandidal activity, indazole, pyrazole, 3-phenyl-1*H*-indazole, drug design

## Abstract

Candidiasis, caused by yeasts of the genus Candida, is the second cause of superficial and mucosal infections and the fourth cause of bloodstream infections. Although some antifungal drugs to treat candidiasis are available, resistant strains to current therapies are emerging. Therefore, the search for new candicidal compounds is certainly a priority. In this regard, a series of indazole and pyrazole derivatives were designed in this work, employing bioisosteric replacement, homologation, and molecular simplification as new anticandidal agents. Compounds were synthesized and evaluated against *C. albicans*, *C. glabrata*, and *C. tropicalis* strains. The series of 3-phenyl-1*H*-indazole moiety (**10a–i**) demonstrated to have the best broad anticandidal activity. Particularly, compound **10g**, with *N*,*N*-diethylcarboxamide substituent, was the most active against *C. albicans* and both miconazole susceptible and resistant *C. glabrata* species. Therefore, the 3-phenyl-1*H*-indazole scaffold represents an opportunity for the development of new anticandidal agents with a new chemotype.

## 1. Introduction

Candidiasis, caused by yeasts of the genus *Candida*, is the second cause of superficial and mucosal infections and the fourth cause of bloodstream infections [1,2]. *Candida* species are normal inhabitants of the oropharynx, gastrointestinal tract, and vagina in humans. However, these species are classified as opportunistic and can change from harmless to pathogenic upon variation of the host environment by physiological or non-physiological changes [1,3].

One of the most frequent mucosal infections is vulvovaginal candidiasis (VVC), which affects millions of women every year and is considered an important public health problem. An estimated 70–75% of women will be affected at some point in their lifetimes, of which approximately 40–50% of initially infected women will have two episodes, and 5–10% of them will develop recurrent VVC [3]. Indeed, this disease is associated with enhanced susceptibility to human immunodeficiency virus (HIV) infection, pregnancy complications, and increased risks of stillbirth or neonatal death. Moreover, when VVC is left untreated, many complications have been associated, such as pelvic inflammatory disease, infertility, ectopic pregnancy, pelvic abscess, spontaneous abortion, and menstrual disorders [3,4]. On the other hand, invasive candidiasis is of greater concern because it is the most common fungal disease among hospitalized patients associated with a 40% mortality rate [2,5,6]. According to estimates, invasive candidiasis affects more than 250,000 people globally every year, and it is the cause of more than 50,000 deaths [5]. In fact, the Centers for Disease Control and Prevention (CDC) estimated that approximately 25,000 cases of candidemia occur in the USA every year [7].

*Candida albicans* is the main etiologic species associated with VVC and invasive candidiasis globally [2,4,5]. However, in the last decades, other *Candida* species such as *C. glabrata*, *C. parapsilosis*, *C. tropicalis*, *C. krusei*, and lastly *C. auris*, have also been demonstrated to cause VVC and invasive candidiasis [2,3,6,7]. The conventional treatments for *Candida* infections are limited to amphotericin B (deoxycholate and various lipid formulations), echinocandins (anidulafungin, caspofungin, and micafungin), azoles (fluconazole, itraconazole, and voriconazole) and flucytosine (5-FC) [6,7,8,9]. Nowadays, the preferred treatment for mucosal and invasive candidiasis is fluconazole, which is active against most *Candida* species and is as effective as amphotericin B, but with fewer side effects. Nevertheless, fluconazole has limited activity against *C. glabrata* and *C. krusei*, and reports of fluconazole-resistant strains have increased [2,5,9].

**Figure 1:**
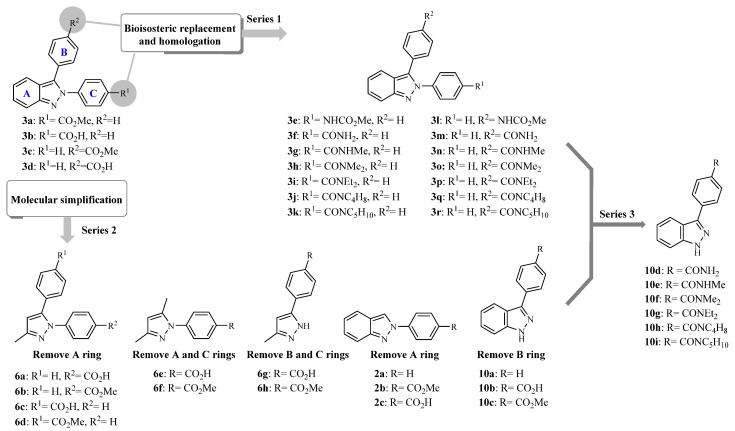
Compounds designed by bioisosteric replacement, homologation, and molecular simplification.

Indazole is an important heterocyclic scaffold in medicinal chemistry since it is associated with a broad range of biological activities, e.g., anti-inflammatory, antiprotozoal, antihypertensive, anticancer, antitumor, antifungal, antibacterial, anti-HIV, antiplatelet, and others [10,11,12,13,14,15,16,17]. Previous studies by our group showed that some 2,3-diphenil-2*H*-indazole derivatives substituted with methyl ester or carboxylic acid groups (compounds **3a**–**d**; Figure 1) have in vitro activity against *C. albicans* and *C. glabrata*. Particularly, compounds **3a** and **3c** showed a minimum inhibitory concentration (MIC) of 3.807 mM and 15.227 mM against *C. albicans* and *C. glabrata*, respectively [17]. However, the information available regarding the structural requirements for anticandidal activity is still limited. Therefore, it is necessary to design and synthesize new indazole derivatives to gain knowledge about the structural modifications required to improve the candicidal activity. As part of our efforts in the search for candicidal compounds, initially, fourteen 2,3-diphenyl-2*H*-indazole derivatives (series 1; Figure 1) were designed by bioisosteric replacement and homologation. It is worth mentioning that esters (e.g., **3a** and **3c**) can be easily hydrolyzed to carboxylic acids and are usually considered prodrugs; therefore, we proposed a replacement by amides that are a similar functional group, but slightly more stable. Different amines were considered to explore the effect of bulky substituents in the activity. Also, fourteen derivatives, which include 3,5-1*H*-pyrazole, 2-phenyl-2*H*-indazole and 3-phenyl-1*H*-indazole, were designed employing molecular simplification strategies by removing rings from the original 2,3-diphenil-2*H*-indazole scaffold (series 2, Figure 1). This strategy has been used to reduce structural complexity, improve physicochemical properties, and to find the minimum molecular features that are necessary for the anticandidal activity. Compounds proposed were synthesized and tested in vitro against four *Candida* strains. Then, taking advantage of the results revealed by the biological evaluations, the third series of six 3-phenyl-1*H*-indazole carboxamides were synthesized and tested.

## 2. Results and Discussion

### 2.1. Synthesis of 2-Phenyl-2H-Indazole and 2,3-Diphenyl-2H-Indazole Derivatives

**Scheme 1:**
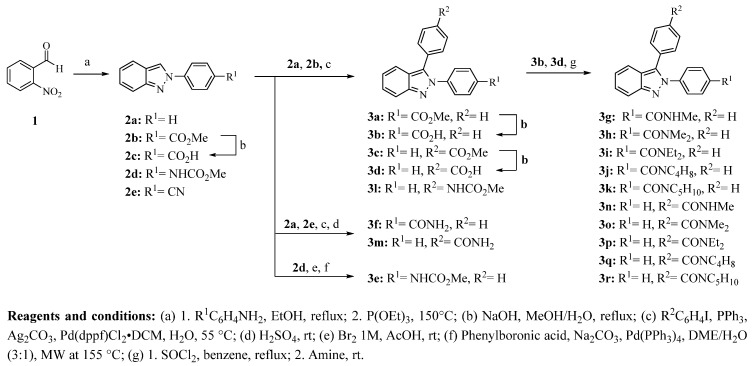
Synthesis of 2*H*-indazole derivatives **2a–e** and **3a–r**.

The synthesis of 2,3-diphenyl-2*H*-indazole derivatives **3a**–**r** was carried out as outlined in Scheme 1. 2-Phenyl-2*H*-indazoles (**2a, 2b**, **2d,** and **2e**) were synthesized by the Cadogan reaction starting from 2-nitrobenzaldehyde (**1**) and the appropriate *p*-substituted aniline under reflux with ethanol to obtain the Schiff’s base, which was then reduced as well as cyclized with P(OEt)_3_ [17,18]. Compound **2c** was obtained by basic hydrolysis of **2b**. Most of the 2,3-diphenyl-2*H*-indazole derivatives were prepared by a palladium-catalyzed arylation of the corresponding 2-phenyl-2*H*-indazole with substituted halobenzene as previously reported [17,19], whereas compound **3e** was synthesized by a Suzuki-Miyaura coupling of methyl (4-(3-bromo-2*H*-indazol-2-yl)phenyl)carbamate with phenylboronic acid [20]. Esters **3a** and **3c** were hydrolyzed with NaOH to give **3b** and **3d**, respectively, in good yields. Additionally, carboxylic acids **3b** and **3d** were converted to acyl chlorides and then treated with the corresponding amine yielding the carboxamides **3g**–**k** and **3n**–**r**. Compounds **3f** and **3m** were prepared by a palladium-catalyzed arylation with iodobenzene or 4-halobenzonitrile followed by acidic conversion to the appropriate carboxamide. Yields for 2-phenyl-2*H*-indazole derivatives (**2a**–**e**) were moderate to high. The palladium-catalyzed arylation yields were slightly lower than that of previously reported data [17,19]. Compound **3e** could not be obtained by this method; instead, a selective bromination of **2d** at position 3, followed by a Suzuki-Miyaura coupling reaction, was applied to give the product in moderate yield (58%).

### 2.2. Synthesis of 1H-Pyrazole and 1H-Indazole Derivatives

**Scheme 2:**
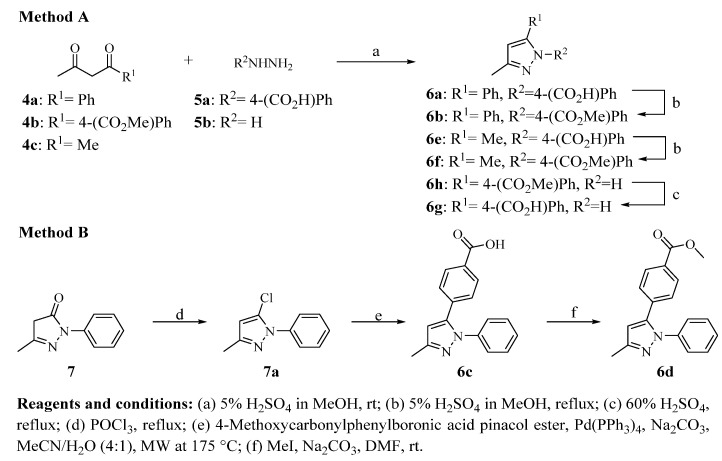
Synthesis of pyrazole derivatives **6a–g**.

The synthesis of 3,5-disubstituted pyrazole derivatives is displayed in Scheme 2. Pyrazoles **6a**, **6e,** and **6h** were prepared by cyclocondensation of the 1,3-dicarbonyl compounds (**4a**–**c**) and 4-hydrazinylbenzoic acid (**5a**) or hydrazine (**5b**) with diluted H_2_SO_4_ in MeOH at room temperature (Scheme 2, Method A) [21]. Compounds **6b** and **6f** were obtained by Fischer–Speier esterification from carboxylic acids **6a** and **6e**, respectively, whereas hydrolysis of **6h** produced the carboxylic acid **6g** in good yields. Compound **6c** was synthesized through a two-step sequence starting from 5-methyl-2-phenyl-2,4-dihydro-3*H*-pyrazol-3-one (**7**), which was first converted to 5-chloro-pyrazole intermediary (**7a**) and then coupled with 4-carbomethoxyphenylboronic acid pinacol ester under microwave irradiation (Scheme 2, Method B) [20]. *O*-Methylation of **6c** with methyl iodide at room temperature yielded the ester **6d**. It is important to mention that all pyrazole derivatives were obtained with moderate to good yields following the proposed chemical synthesis.

**Scheme 3:**
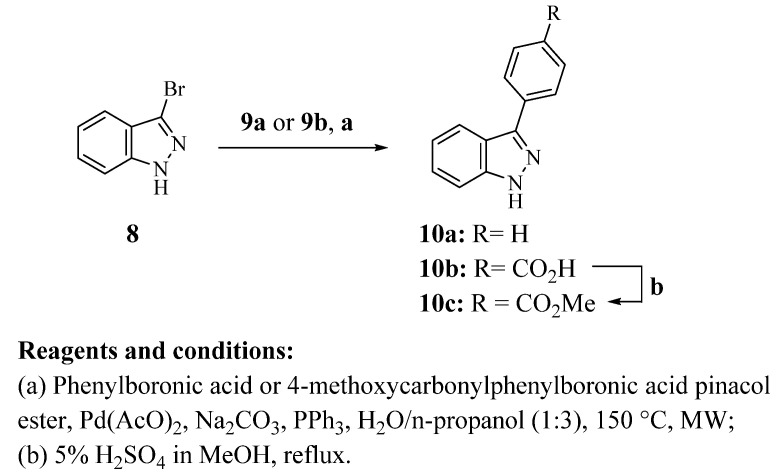
Synthesis of 3-phenyl-1*H*-indazole derivatives **10a–c**. All synthesized compounds were characterized by ^1^H NMR and ^13^C NMR spectra and the new structures were also characterized by mass spectrometry. The nuclear magnetic resonance and mass spectra of compounds can be found in Figures S2–S71 in the Supplementary Materials.

Finally, a slight modification of the method reported by Huff et al. was performed, since 3-bromo-1*H*-indazole (**8**) was coupled under microwave irradiation with phenylboronic acid (**9a**) or 4-methoxycarbonylphenylboronic acid pinacol ester (**9b**) to afford the 3-phenyl-1*H*-indazole derivatives **10a** and **10b**, respectively (Scheme 3) [22]. Fischer–Speier esterification of compound **10b** gave **10c** in good yield.

### 2.3. Anticandidal Activity

Some structural changes were performed to gain knowledge about the features that would improve the anticandidal activity of 2,3-diphenyl-2*H*-indazole derivatives. The methyl ester group of **3a** and **3c**, and the carboxylic acid group of **3b** and **3d** were replaced by methyl carbamate (**3e**, **3l**), as well as carboxamide and *N*-substituted carboxamides (**3f**–**k** and **3m**–**r**). The anticandidal activity of these new derivatives was tested against *C. albicans*, miconazole susceptible and resistant *C. glabrata* (ATCC 90030 and 32554, respectively) and *C. tropicalis* (ATCC 750) using the cylinder-plate method [23], which results are presented in Table 1. Overall, *N*-substituted carboxamides **3h**–**k** and **3n**–**r** showed activity against *C. albicans* at 1 mM. Particularly, compound **3j** showed the maximum inhibition of *C. albicans* growth at 10 mM, but it also had activity at 1 mM. It also showed weak activity against miconazole-resistant *G. glabrata* at 10 mM. Compounds **3h** and **3p** were active against *C. albicans*, and miconazole-resistant *C. glabrata* species at 1 mM. Regarding carboxamide derivatives, compound **3f** was active just against *C. tropicalis* at 10 mM, while compound **3m** had weak anticandidal activity against *C. albicans* and miconazole susceptible *C. glabrata* at 10 mM. Concerning methyl carbamate derivatives **3e** and **3l**, they did not show activity against *C. albicans*, but **3e** was active against *C. tropicalis* at 10 mM and **3l** had activity against susceptible and resistant miconazole *C. glabrata* at 10 mM. All these results suggest that compounds **3h** and **3p** (*N*,*N-*dimethyl and *N*,*N*-diethyl carboxamide, respectively) had the best candicidal activity against *C. albicans* and miconazole resistant *C. glabrata* followed by **3j** and **3r** (pyrrolidine and piperidine carboxamide, respectively). It is worth noticing that these compounds demonstrated better activity against the miconazole-resistant *C. glabrat*a than to the susceptible *C. glabrata*.

On the other hand, to understand the effect of the molecular simplification of the indazole nucleus on the anticandidal activity 3,5-1*H*-pyrazole (**6a**–**h**), 2-phenyl-1*H*-indazole (**2a**–**c**) and 3-phenyl-1*H*-indazole derivatives (**10a**–**c**) were tested against *Candida* strains. Their anticandidal activity is presented in Table 1. Noteworthy, most of the 3,5-1*H*-pyrazole derivatives (**6b**–**d** and **6f**) showed anticandidal activity at lower concentrations than those of 2,3-diphenyl indazole analogs (**3a**–**d**). These were active against *C. albicans* and miconazole-resistant *C. glabrat*a at 1 mM. Compounds **6a** and **6e** showed only activity against miconazole-resistant *C. glabrat*a (at 1 mM), and **6g** and **6h** were only active against *C. albicans* at 1 mM. It is worth noticing that the pyrazole **6f** exhibited broad growth inhibition against *C. albicans*, miconazole susceptible and resistant *C. glabrata-strains* and *C. tropicalis*.

Regarding the activity of 2-phenyl-2*H*-indazole (**2a**–**c**) and 3-phenyl-1*H*-indazole (**10a**–**c**) derivatives, these compounds showed anticandidal activity at lower concentrations than 2,3-diphenyl indazole and 3,5-1*H*-pyrazole derivatives. Compound **2b** had the best activity against *C. albicans* and miconazole susceptible *C. glabrata* strain at 100 µM and 1 mM respectively, while **2c** had the best activity against miconazole resistant *C. glabrata* at 1 mM. The 3-phenyl-1*H*-indazole compounds, **10a** and **10c**, displayed broad activity against *C. albicans*, miconazole susceptible and resistant *C. glabrata.* Notably, the 3-phenylindazole **10a** had activity against *C. albicans* and the miconazole resistant *C. glabrata* strain at 100 µM, and to miconazole susceptible *C. glabrata* at 1 mM, whereas **10c** had the best inhibition against *C. albicans* and miconazole susceptible *C. glabrata* at 100 µM. Concerning **10b**, it showed activity against *C. albicans* at 1 mM and miconazole susceptible *C. glabrata* at 100 µM. Additionally, to discard the DMSO effect in the growth inhibition, a control of solvent was included in all assays (Figure S1, supporting material).

**Figure 2:**
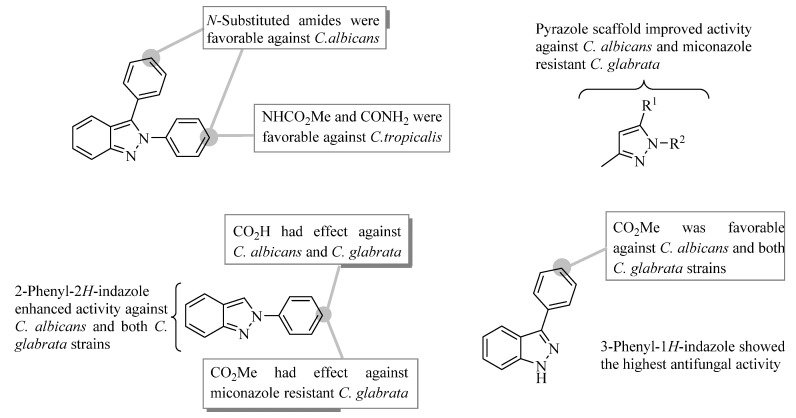
Principally structure-activity relationship features derived from compounds tested.

According to these results, principally structure-activity relationship features derived from compounds tested are exhibited in Figure 2. The replacement of the carbonyl group by *N*-substituted carboxamides on 2,3-diphenyl-2*H*-indazole nucleus led to an increase in the growth inhibitory activity against *C. albicans*. The molecular simplification of 2,3-diphenylindazole improved the antifungal activity against *Candida* strains tested. The 3,5-disubstituted pyrazoles displayed better growth inhibition than the 2,3-diphenylindazole analogs against *C. albicans* and miconazole resistant *C*. *glabrata.* At the same time, 2-phenyl-2*H*-indazoles were active against *C. albicans* and both *C. glabrata* strains. It is important to emphasize that 3-phenyl-1*H*-indazole derivatives showed the highest antifungal activity against all *Candida* strains tested except for *C. tropicali*s.

It is important to mention that prototype compound **10a**, which represents the nucleus for the most active compounds, was also tested on three representative human cell lines employing the MTT method [17]. The results showed 100% of cellular viability on HeLa (human cervix), K562 (human chronic myelogenous leukemia) and SW620 (human colon) cell lines after 48h of compound exposition at 50 µM.

### 2.4. Synthesis and Determination of Minimum Inhibitory Concentration (MIC) of 3-Phenyl-1H-Indazole Derivatives

**Scheme 4:**
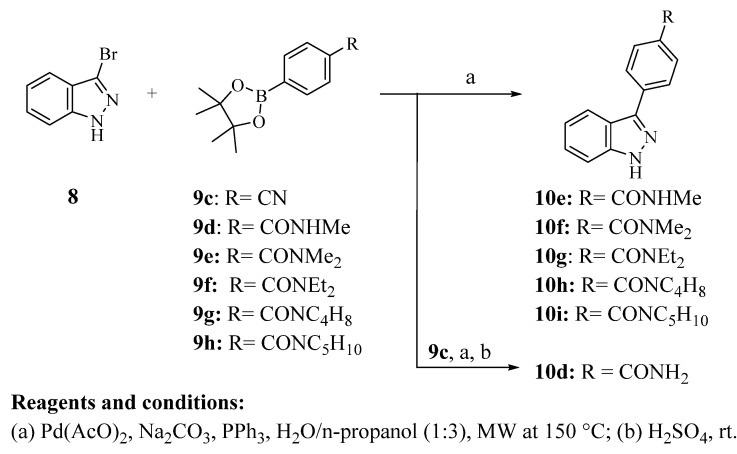
Synthesis of carboxamide 3-phenyl-1*H*-indazole derivatives.

Considering all the data obtained about 2,3-diphenyl indazole amides, as well as molecular simplification used to select the best scaffold, six new 3-phenyl-1*H*-indazole derivatives bearing small or bulky amides were synthesized (**10d**–**i**). These compounds were synthetized through the Suzuki-Miyaura reaction of coupling 3-bromo-1*H*-indazole (**8**) with several 4-phenylboronic acid pinacol esters (**9c**–**h**) (Scheme 4) [22]. Yields of carboxamide derivatives were lower compared to their unsubstituted or carboxylic acid analogs. The above indicates that phenylboronic acid pinacol esters with a carboxamide group are less reactive.

The in vitro anticandidal assays against *C. albicans* and *C. glabrata* strains of 3-phenyl-1*H*-indazole derivates **10a–i** were carried out following the cylinder-plate method previously described [23]. The minimum inhibitory concentration (MIC) values were determined and are shown in Table 2. It is noteworthy that fluconazole and miconazole were used as reference drugs, and in addition, the purity of the compounds was determined by quantitative NMR (purity > 95%) to ensure that the observed MIC values were caused by the reported compounds (Table S1 and Figure S72, supporting material). Most of the amides kept their activity against all *Candida* strains, similar to compounds **10a**–**c**. Indeed, some of them showed a slight increase in anticandidal activity. From this series, compounds **10c**, **10g**, and **10i** had the best activity against *C. albicans*. In contrast, **10f** is the most active compound against both miconazole susceptible and resistant *C. glabrata*. In addition, **10g** was active against miconazole-resistant *C. glabrata*. It is important to emphasize that compounds with bulky carboxamides (**10g**–**i**) had better anticandidal activity against *C. albicans* than small carboxamides (**10d**–**f**). These results indicated that the carboxamide group on the 3-phenyl-1*H*-indazole scaffold kept antifungal activity against tested *C. albicans* and *C. glabrata* strains. The MIC determination of the compounds tested showed to be less active than the reference drugs. However, the antifungal activity of the proposed compounds is still relevant and can be considered as leads for the development of new anticandidal agents.

## 3. Materials and Methods

### 3.1. Chemistry

All chemicals and starting materials were obtained from Sigma-Aldrich (Toluca, MC, Mexico). Reactions were monitored by TLC on 0.2 mm percolated silica gel 60 F254 plates (Merck, Darmstadt, Germany) and visualized by irradiation with a UV lamp (Upland, CA, USA). Silica gel 60 (70–230 mesh, Düren, Germany) was used for column chromatography. Melting points were determined in open capillary tubes with a Büchi M-565 melting point apparatus (Flawil, Switzerland). Microwave-assisted reactions were carried out in a monomodal reactor equipped with a hydraulic pressure sensing device and an infrared temperature-sensor (Anton Parr Monowave 300, Anton Parr, Graz, Austria). ^1^H-NMR and ^13^C-NMR spectra were measured with an Agilent DD2 spectrometer (Santa Clara, CA, USA), operating at 600 MHz and 151 MHz for ^1^H and ^13^C, respectively. Chemical shifts are given in parts per million relatives to tetramethylsilane (TMS, δ = 0); *J* values are given in Hz. Splitting patterns are expressed as follow: s, singlet; d, doublet; t, triplet; q, quartet; dd, doublet of doublet; dt, doublet of triplete; ddd, doublet of doublets of doublets; m, multiplet; bs, broad singlet. High-resolution mass spectra were recorded on a Bruker ESI/APCI-TOF, MicroTOF-II-Focus spectrometer (Billerica, MA, USA) by electrospray ionization (ESI) and low-resolution mass spectra were recorded on a Water Xevo TQ-MS by ESI. The purity of compounds **10a–i** was determined by quantitative NMR spectroscopy (qNMR) using sodium 2,2-dimethyl- 2-silapentane-5-sulfonate (DSS) as internal standard (Cambridge Isotope Laboratories, Inc., Tewksbury, MA, USA). All compounds were named using the automatic name generator tool implemented in ChemDraw 19.1.1.21 software (PerkinElmer, Waltham, MA, USA), according to IUPAC rules.

### 3.2. General Procedure for the Synthesis

#### 3.2.1. 2-Phenyl-2*H*-Indazole Derivatives (**2a**, **2b**, **2d**, and **2e**)

2-Phenyl-2*H*-indazole derivatives were synthesized employing a slight modification of the Cadogan method [17,18]. A mixture of 2-nitrobenzaldehyde (200 mg, 1.32 mmol) and aniline or the corresponding substituted aniline (1.32 mmol) was dissolved in ethanol (10 mL) and heated at reflux for 2 h. Then, the solvent was removed under vacuum to give the appropriate 1-(2-nitrophenyl)-*N*-phenylmethanimine. This same reaction was carried out at room temperature to obtain the methyl 4-((2-nitrobenzylidene)amino)benzoate. Later, triethyl phosphite (3.96 mmol) was added and heated at 150 °C employing an oil bath for 2 h under N_2_ atmosphere, until the starting material was consumed. After cooling, the mixture was treated with 20 mL of 5% hydrogen peroxide solution, and the product was extracted with ethyl acetate (3 × 15 mL). The combined organic layers were dried over Na_2_SO_4_ and concentrated in vacuo. The crude product was purified by column chromatography using hexane/ethyl acetate (80:20). Only compound **2b** was isolated by vacuum filtration and washed with cold ethanol to give a pure compound.

2-Phenyl-2*H*-indazole (**2a**). White solid (60% yield); m.p.: 81.2–81.6 °C (lit. [18]: 81–82 °C); the spectroscopic data matched previously reported data [17,24]: ^1^H NMR (600 MHz, CDCl_3_) δ 8.40 (d, *J* = 0.9 Hz, 1H), 7.91–7.88 (m, 2H), 7.79 (dd, *J* = 8.8, 0.9 Hz, 1H), 7.70 (dt, *J* = 8.5, 1.0 Hz, 1H), 7.54–7.50 (m, 2H), 7.41–7.37 (m, 1H), 7.32 (ddd, *J* = 8.8, 6.6, 1.0 Hz, 1H), 7.11 (ddd, *J* = 8.4, 6.6, 0.7 Hz, 1H); ^13^C NMR (151 MHz, CDCl_3_): δ 149.78, 140.52, 129.54, 127.88, 126.81, 122.76, 122.44, 120.99, 120.39, 120.37, 117.94.

Methyl 4-(2*H*-indazol-2-yl) benzoate (**2b**). After completion of the reaction, the product was filtrated and washed with cold MeOH. White solid (38% yield); m.p.: 185.8–186.2 °C (lit. [25]: 186–187 °C); the spectroscopic data matched previously reported data [17]: ^1^H NMR (600 MHz, CDCl_3_) δ 8.47 (d, *J* = 0.7 Hz, 1H), 8.22–8.18 (m, 2H), 8.02–7.99 (m, 2H), 7.77 (dd, *J* = 8.8, 0.8 Hz, 1H), 7.69 (d, *J* = 8.5 Hz, 1H), 7.33 (ddd, *J* = 8.8, 6.6, 1.0 Hz, 1H), 7.14–7.10 (m, 1H), 3.95 (s, 3H); ^13^C NMR (151 MHz, CDCl_3_) δ 166.19, 150.19, 143.64, 131.16, 129.27, 127.45, 123.01, 122.98, 120.47, 120.26, 118.06, 52.33.

Methyl (4-(2*H*-indazol-2-yl)phenyl)carbamate (**2d**). Pale orange solid (17% yield); m.p.: 163.7–165.0 °C; ^1^H NMR (600 MHz, CDCl_3_) δ 8.46 (bs, 1H), 8.38 (d, *J* = 0.9 Hz, 1H), 7.83–7.79 (m, 2H), 7.76 (dd, *J* = 8.8, 0.9 Hz, 1H), 7.70 (d, *J* = 8.4 Hz, 1H), 7.64 (d, *J* = 7.6 Hz, 2H), 7.31 (ddd, *J* = 8.7, 6.6, 1.1 Hz, 1H), 7.10 (ddd, *J* = 8.4, 6.6, 0.8 Hz, 1H), 3.80 (s, 3H); ^13^C NMR (151 MHz, CDCl_3_) δ 154.27, 149.48, 138.52, 135.46, 126.59, 122.62, 122.20, 121.44, 120.30, 120.25, 119.20, 117.62, 52.17; MS [M+H]^+^ *m*/*z* 268.12.

4-(2*H*-indazol-2-yl)benzonitrile (**2e**). Yellow solid (19% yield); m.p.; 162.0–163.5 °C; (lit. [24]: 163.4–164.6 °C); the spectroscopic data matched previously reported data [24]: ^1^H NMR (600 MHz, CDCl_3_) δ 8.47 (d, *J* = 0.9 Hz, 1H), 8.10–8.05 (m, 2H), 7.85–7.80 (m, 2H), 7.76 (dd, *J* = 8.8, 0.9 Hz, 1H), 7.70 (dt, *J* = 8.5, 1.0 Hz, 1H), 7.35 (ddd, *J* = 8.8, 6.6, 1.1 Hz, 1H), 7.14 (ddd, *J* = 8.5, 6.6, 0.8 Hz, 1H); ^13^C NMR (151 MHz, CDCl_3_) δ 150.37, 143.33, 133.69, 127.87, 123.36, 123.14, 120.85, 120.49, 120.37, 118.13, 118.08, 111.18; MS [M + H]^+^ *m*/*z* 220.11.

#### 3.2.2. 4-(2*H*-Indazol-2-yl) Benzoic Acid (**2c**)

Methyl 4-(2*H*-indazol-2-yl) benzoate (300 mg, 1.26 mmol) was dissolved in methanol (10 mL) and an aqueous solution of NaOH (3.77 mmol in 5 mL of water) was added. The reaction mixture was heated under reflux for 5 h. After completion, the reaction mixture was cooled on an ice bath and acidified to pH 1 with HCl to induce precipitation. The solid was filtered under vacuum and dried.

4-(2*H*-Indazol-2-yl) benzoic acid (**2c**). White solid (96% yield); m.p.: 288.3–288.5 °C (lit. [25]: 286–288 °C); the spectroscopic data matched previously reported data [17]: ^1^H NMR (600 MHz, DMSO-*d*6) δ 13.14 (bs, 1H), 9.22 (s, 1H), 8.26 (d, *J* = 8.6 Hz, 2H), 8.15 (d, *J* = 8.6 Hz, 2H), 7.79 (d, *J* = 8.4 Hz, 1H), 7.74 (d, *J* = 8.7 Hz, 1H), 7.41–7.30 (m, 1H), 7.19–7.08 (m, 1H); ^13^C NMR (151 MHz, DMSO-*d*6) δ 166.47, 149.23, 142.84, 130.83, 129.65, 127.28, 122.55, 122.44, 122.03, 120.99, 119.86, 117.49.

#### 3.2.3. 2,3-Diphenyl-2*H*-Indazole Derivatives (**3a**, **3c**, and **3l**)

2,3-Diphenyl-2*H*-indazole derivatives **3a**, **3c**, and **3l** were synthesized by a palladium-catalyzed arylation previously described by Ohnmacht et al. [19]. Compounds **3a** and **3c** were synthesized using the appropriate 2-phenyl-2*H*-indazole and the substituted 4-iodobenzene, whereas compound **3l** was synthesized from 2-phenyl-2*H*-indazole and methyl(4-bromophenyl) carbamate.

Methyl 4-(3-phenyl-2*H*-indazol-2-yl) benzoate (**3a**). Pale yellow solid (40% yield); m.p.: 152.4*–*154.9 °C (lit. [17]: 152.4*–*154.9 °C); the spectroscopic data matched previously reported data [17]: ^1^H NMR (600 MHz, CDCl_3_) δ 8.09–8.03 (m, 2H), 7.80 (d, *J* = 8.8 Hz, 1H), 7.69 (d, *J* = 8.5 Hz, 1H), 7.57–7.49 (m, 2H), 7.41 (dt, *J* = 3.7, 1.2 Hz, 3H), 7.35 (dd, *J* = 7.5, 2.0 Hz, 3H), 7.15 (ddd, *J* = 8.5, 6.6, 0.8 Hz, 1H), 3.93 (s, 3H); ^13^C NMR (151 MHz, CDCl_3_) δ 166.19, 149.33, 143.75, 135.68, 130.40, 129.68, 129.61, 128.96, 128.64, 127.44, 125.68, 122.88, 122.06, 120.52, 117.79, 52.32.

Methyl 4-(2-phenyl-2*H*-indazol-3-yl) benzoate (**3c**). Pale yellow solid (76% yield): m.p.: 164.5–166.3 °C (lit. [17]: 164.5–166.3 °C) the spectroscopic data matched previously reported data [17,26]: ^1^H NMR (600 MHz, CDCl_3_) δ 8.09–8.03 (m, 2H), 7.82 (d, *J* = 8.8 Hz, 1H), 7.72 (dt, *J* = 8.5, 0.9 Hz, 1H), 7.46–7.36 (m, 8H), 7.19 (ddd, *J* = 8.5, 6.5, 0.6 Hz, 1H), 3.93 (s, 3H); ^13^C NMR (151 MHz, CDCl_3_) δ 166.55, 149.08, 139.99, 134.37, 134.13, 129.97, 129.66, 129.49, 129.18, 128.59, 127.14, 126.04, 123.18, 121.90, 120.09, 118.02, 52.29.

Methyl (4-(2-phenyl-2*H*-indazol-3-yl)phenyl)carbamate (**3l**). Pale brown solid (26% yield): m.p.: 197.9–199.7 °C; ^1^H NMR (600 MHz, DMSO-*d*_6_) δ 9.85 (s, 1H), 7.70 (ddt, *J* = 24.0, 8.5, 0.9 Hz, 2H), 7.53 (d, *J* = 8.6 Hz, 2H), 7.50–7.41 (m, 5H), 7.37 (ddd, *J* = 8.7, 6.6, 1.1 Hz, 1H), 7.30–7.26 (m, 2H), 7.14 (ddd, *J* = 8.5, 6.5, 0.8 Hz, 1H), 3.68 (s, 3H); ^13^C NMR (151 MHz, DMSO-*d*_6_) δ 154.36, 148.61, 140.36, 139.82, 135.37, 130.46, 129.52, 128.89, 127.25, 126.45, 123.41, 122.67, 121.46, 120.95, 118.66, 117.78, 52.20; MS [M+H]^+^ *m*/*z* 344.1381.

#### 3.2.4. 2,3-Diphenyl-2*H*-Indazole Derivatives (**3b** and **3d**)

Employing the hydrolysis method 3.2.2 described above, compounds **3b** and **3d** were prepared from their esters **3a** or **3c**, respectively.

4-(3-Phenyl-2*H*-indazol-2-yl) benzoic acid (**3b**). White solid (70% yield); m.p.: 129.2–130.1 °C (lit. [17]: 129.2–130.1 °C); the spectroscopic data matched previously reported data [17]: ^1^H NMR (600 MHz, DMSO-*d*_6_) δ 8.01 (d, *J* = 8.6 Hz, 2H), 7.77 (d, *J* = 8.8 Hz, 1H), 7.69 (d, *J* = 8.5 Hz, 1H), 7.57 (d, *J* = 8.6 Hz, 2H), 7.44 (dddd, *J* = 11.9, 7.6, 5.4, 3.9 Hz, 6H), 7.21–7.15 (m, 1H); ^13^C NMR (151 MHz, DMSO-*d*_6_) δ 166.49, 148.52, 143.08, 135.26, 130.53, 130.15, 129.52, 129.03, 128.95, 128.71, 127.26, 125.99, 122.81, 121.38, 120.40, 117.49.

4-(2-Phenyl-2*H*-indazol-3-yl) benzoic acid (**3d**). White solid (88% yield); mp: 296.2–298.2 °C (lit. [17]: 296.2–298.2 °C); the spectroscopic data matched previously reported data [17]: ^1^H NMR (600 MHz, DMSO-*d*_6_) δ δ 7.92 (d, *J* = 8.2 Hz, 2H), 7.75 (d, *J* = 8.7 Hz, 1H), 7.71 (d, *J* = 8.5 Hz, 1H), 7.50–7.41 (m, 5H), 7.38 (ddd, *J* = 8.7, 6.6, 0.9 Hz, 1H), 7.28 (d, *J* = 8.3 Hz, 2H), 7.19–7.13 (m, 1H); ^13^C NMR (151 MHz, DMSO-*d*_6_) δ 169.13, 148.11, 140.15, 139.79, 135.10, 129.32, 129.17, 128.98, 128.37, 128.26, 126.77, 125.88, 122.34, 121.02, 120.38, 117.29.

#### 3.2.5. 2,3-Diphenyl-2*H*-Indazole Derivatives (**3f** and **3m**)

The proper 2-phenyl-2*H*-indazole (**2a** or **2e**) and 4-bromobenzonitrile or 4-iodobenzene were reacted employing the previously described method by Ohnmacht et al. [19] to give the benzonitrile derivative. Then, the intermediate was dissolved and stirred in H_2_SO_4_ (1 mL) at room temperature overnight. After completion, the mixture was poured into ice-water (15 mL) to induce precipitation. The solid was filtered under vacuum and dried.

4-(3-Phenyl-2*H*-indazol-2-yl)benzamide (**3f**). White solid (57% yield): m.p.: 221.6–222.8 °C; ^1^H NMR (600 MHz, DMSO-*d*_6_) δ 8.09 (s, 1H), 7.97–7.92 (m, 2H), 7.76 (dt, *J* = 8.8, 0.9 Hz, 1H), 7.68 (dt, *J* = 8.5, 1.0 Hz, 1H), 7.55–7.37 (m, 9H), 7.18 (ddd, *J* = 8.5, 6.6, 0.8 Hz, 1H). ^13^C NMR (151 MHz, DMSO-*d*_6_) δ 167.30, 148.85, 142.33, 135.65, 134.40, 129.97, 129.50, 129.45, 129.09, 128.77, 127.59, 126.14, 123.16, 121.78, 120.81, 117.91; MS [M+H]^+^ *m*/*z* 314.1294.

4-(2-Phenyl-2*H*-indazol-3-yl)benzamide (**3m**). Pale yellow solid (34% yield): m.p.: 230.6–232.7 °C; ^1^H NMR (600 MHz, DMSO-*d*_6_) δ 8.05 (s, 1H), 7.93 (d, *J* = 8.3 Hz, 2H), 7.77 (d, *J* = 8.7 Hz, 1H), 7.72 (d, *J* = 8.5 Hz, 1H), 7.47 (ddd, *J* = 15.3, 11.3, 7.9 Hz, 8H), 7.40 (dd, *J* = 11.7, 3.7 Hz, 1H), 7.20 (dd, *J* = 8.1, 6.9 Hz, 1H); ^13^C NMR (151 MHz, DMSO-*d*_6_) δ 167.63, 148.69, 140.16, 134.64, 134.28, 132.31, 129.70, 129.64, 129.16, 128.40, 127.41, 126.57, 123.30, 121.71, 120.68, 117.96; MS [M+H]^+^ *m*/*z* 314.1281.

#### 3.2.6. Methyl (4-(3-Phenyl-2*H*-indazol-2-yl)phenyl) Carbamate (**3e**)

Methyl (4-(phenyl-2*H*-indazole-2-yl)phenyl) carbamate **2d** (500 mg, 1.88 mmol) was dissolved in acetic acid (5 mL). Bromine (1.8 mL of 1 M solution in acetic acid) was slowly added at 0–5 °C and then led to warm at room temperature and stirred overnight. After completion of the reaction, ice-water was added and the solid formed was filtered and dried under vacuum. The crude intermediate (0.5 mmol) was treated with phenylboronic acid (0.55 mmol), Na_2_CO_3_ (1.5 mmol), Pd(PPh_3_)_4_ (0.01 mmol) and 3 mL of DME/water (3:1). The mixture was heated under microwave irradiation at 155 °C for 30 min [20]. After cooling, the solvent was removed under vacuum and the obtained product was purified by column chromatography using hexane/ethyl acetate (60:40).

Methyl (4-(3-phenyl-2*H*-indazol-2-yl)phenyl)carbamate (**3e**). White solid (58% yield): m.p.: 201.5–202.5 °C; ^1^H NMR (600 MHz, DMSO-*d*_6_) δ 9.91 (s, 1H), 7.73 (dt, *J* = 8.8, 0.9 Hz, 1H), 7.67 (dt, *J* = 8.5, 1.0 Hz, 1H), 7.53 (d, *J* = 8.9 Hz, 2H), 7.48–7.44 (m, 2H), 7.43–7.39 (m, 1H), 7.39–7.31 (m, 5H), 7.15 (ddd, *J* = 8.5, 6.6, 0.8 Hz, 1H), 3.69 (s, 3H); ^13^C NRM (151 MHz, DMSO-*d*_6_) δ 154.39, 148.48, 139.76, 135.26, 134.59, 129.87, 129.71, 129.32, 128.83, 127.16, 127.02, 122.81, 121.45, 120.71, 118.52, 117.77, 52.25; MS [M+H]^+^ *m*/*z* 344.1391.

#### 3.2.7. 2,3-Diphenyl-2*H*-Indazole Carboxamides (**3g**–**k** and **3n**–**r**)

Method A: To a solution of carboxylic acids **3b** or **3d** (250 mg, 0.8 mmol) in benzene (5 mL), SOCl_2_ (0.35 mL, 4.8 mmol) was added. The mixture was heated at 70 °C for 4 h. After completion, the excess of SOCl_2_ was distilled-off at reduced pressure (3 × 5 mL of benzene) to give the acyl chloride intermediate and then stirred with an excess of the adequate amine (16 mmol) at room temperature for 30 min. The mixture was poured in methanol and heated until solids were dissolved. Water was added to induces the precipitation of the compound. The formed solid was separated by vacuum filtration. Finally, the crude product was purified by column chromatography using hexane/ethyl acetate (80:20).

*N,N-*Diethyl-4-(3-phenyl-2*H*-indazol-2-yl)benzamide (**3i**). Pale yellow solid (80% yield); m.p.: 136.5–138.0 °C. ^1^H NMR (600 MHz, CDCl_3_) δ 7.79 (d, *J* = 8.8 Hz, 1H), 7.71 (d, *J* = 8.5 Hz, 1H), 7.52–7.48 (m, 2H), 7.48–7.35 (m, 8H), 7.15 (ddd, *J* = 8.3, 6.5, 0.5 Hz, 1H), 3.55 (s, 2H), 3.24 (s, 2H), 1.25 (s, 3H), 1.09 (s, 3H); ^13^C NMR (151 MHz, CDCl_3_) δ 170.25, 149.18, 140.78, 137.00, 135.61, 129.71, 129.57, 128.99, 128.57, 127.29, 127.21, 125.93, 122.73, 121.91, 120.59, 117.72, 43.35, 39.47, 14.21, 12.90; MS [M+H]^+^ *m*/*z* 370.23.

(4-(3-Phenyl-2*H*-indazol-2-yl)phenyl)(pyrrolidin-1-yl)methanone (**3j**). Pale brown solid (40% yield); m.p.: 186.1–186.2 °C; ^1^H NMR (600 MHz, CDCl_3_) δ 7.79 (d, *J* = 8.8 Hz, 1H), 7.71 (d, *J* = 8.5 Hz, 1H), 7.61–7.52 (m, 2H), 7.52–7.45 (m, 2H), 7.45–7.34 (m, 6H), 7.18–7.12 (m, 1H), 3.65 (t, *J* = 7.0 Hz, 2H), 3.40 (t, *J* = 6.6 Hz, 2H), 2.00–1.93 (m, 2H), 1.92–1.85 (m, 2H); ^13^C NMR (151 MHz, CDCl_3_) δ 168.60, 149.15, 141.22, 136.84, 135.56, 129.66, 129.56, 128.95, 128.54, 127.97, 127.26, 125.70, 122.71, 121.90, 120.54, 117.70, 49.56, 46.29, 26.40, 24.41; MS [M+H]^+^ *m*/*z* 368.24.

(4-(3-Phenyl-2*H*-indazol-2-yl)phenyl)(piperidin-1-yl)methanone (**3k**). White solid (73% yield); m.p.: 136.0–138.0 °C; ^1^H NMR (600 MHz, CDCl_3_) δ 7.81 (d, *J* = 8.8 Hz, 1H), 7.73 (d, *J* = 8.5 Hz, 1H), 7.48–7.34 (m, 10H), 7.20–7.14 (m, 1H), 3.71 (bs, 2H), 3.38 (bs, 2H), 1.85–1.57 (bs, 6H); ^13^C NMR (151 MHz, CDCl_3_) 169.56, 149.00, 140.02, 136.12, 134.41, 131.01, 129.56, 129.13, 128.45, 127.41, 127.04, 126.02, 122.85, 121.81, 120.22, 117.89, 48.77, 43.20, 26.59, 25.54, 24.54; MS [M+H]^+^ *m*/*z* 382.26.

*N*,*N-*Diethyl-4-(2-phenyl-2*H*-indazol-3-yl)benzamide (**3p**). White solid (88 % yield); m.p.: 148.4–149.6 °C*;*^1^H NMR (600 MHz, CDCl_3_) δ 7.81 (d, *J* = 8.8 Hz, 1H), 7.73 (d, *J* = 8.5 Hz, 1H), 7.49–7.36 (m, 10H), 7.19–7.14 (m, 1H), 3.56 (bs, 2H), 3.29 (bs, 2H), 1.26 (bs, 3H), 1.14 (bs, 3H); ^13^C NMR (151 MHz, CDCl_3_) δ 170.57, 149.03, 140.06, 136.93, 134.47, 130.79, 129.59, 129.13, 128.47, 127.06, 126.92, 126.05, 122.86, 121.80, 120.24, 117.92, 43.39, 39.41, 14.25, 12.89; MS [M+H]^+^ *m*/*z* 370.26.

(4-(2-Phenyl-2*H*-indazol-3-yl)phenyl)(pyrrolidin-1-yl)methanone (**3q**). White solid (37% yield); m.p.: 167.0–168.8 °C; ^1^H NMR (600 MHz, CDCl_3_) δ 7.81 (d, *J* = 8.8 Hz, 1H), 7.72 (dt, *J* = 8.5, 0.8 Hz, 1H), 7.56 (d, *J* = 8.3 Hz, 2H), 7.46–7.36 (m, 8H), 7.17 (ddd, *J* = 8.4, 6.5, 0.7 Hz, 1H), 3.66 (t, *J* = 7.0 Hz, 2H), 3.46 (t, *J* = 6.6 Hz, 2H), 2.01–1.95 (m, 2H), 1.94–1.88 (m, 2H); ^13^C NMR (151 MHz, CDCl_3_) δ 168.95, 149.03, 140.04, 136.82, 134.46, 131.39, 129.44, 129.15, 128.49, 127.68, 127.09, 126.04, 122.90, 121.84, 120.23, 117.91, 49.60, 46.33, 26.46, 24.43; MS [M+H]^+^ *m*/*z* 368.24.

(4-(2-Phenyl-2*H*-indazol-3-yl)phenyl)(piperidin-1-yl)methanone (**3r**). White solid (90% yiled); m.p.: 198.2–199.2 °C; ^1^H NMR (600 MHz, CDCl_3_) δ 7.81 (d, *J* = 8.8 Hz, 1H), 7.73 (d, *J* = 8.5 Hz, 1H), 7.55–7.28 (m, 10H), 7.20–7.14 (m, 1H), 3.72 (bs, 2H), 3.38 (bs, 2H), 1.69 (bs, 6H).^13^C NMR (151 MHz, CDCl_3_) δ 169.56, 149.00, 140.02, 136.12, 134.41, 131.01, 129.56, 129.13, 128.45, 127.41, 127.04, 126.02, 122.85, 121.81, 120.22, 117.89, 48.77, 43.22, 26.57, 25.59, 24.54; MS [M+H]^+^ *m*/*z* 382.26.

Method B: The acyl chlorides were synthesized employing the same procedure as described in method A. Next, the crude acyl chloride was mixed with methylamine hydrochloride or *N*,*N*-dimethylamine hydrochloride (1 mmol) and dissolved in anhydrous CH_2_Cl_2_ (3 mL). Then, triethylamine (1.0 mmol) was slowly added to the mixture and stirred at room temperature for 2 h. After completion, the mixture was concentrated in vacuo. The product was purified by column chromatography using hexane/ethyl acetate (70:30).

*N-*Methyl-4-(3-phenyl-2*H*-indazol-2-yl)benzamide (**3g**). Pale brown solid (43% yield); m.p.:194.3–195.4 °C. ^1^H NMR (600 MHz, CDCl_3_) δ 7.78 (dt, *J* = 8.9, 0.9 Hz, 1H), 7.77–7.74 (m, 2H), 7.70 (dt, *J* = 8.5, 0.9 Hz, 1H), 7.53–7.43 (m, 2H), 7.43–7.30 (m, 6H), 7.15 (ddd, *J* = 8.5, 6.5, 0.8 Hz, 1H), 6.38 (d, *J* = 4.3 Hz, 1H), 3.01 (d, *J* = 4.8 Hz, 3H); ^13^C NMR (151 MHz, CDCl_3_) δ 167.20, 149.22, 142.42, 135.66, 134.18, 129.65, 129.53, 128.94, 128.60, 127.68, 127.40, 125.88, 122.81, 121.95, 120.53, 117.66, 26.89; MS [M+H]^+^ *m*/*z* 328.1439.

*N,N*-Dimethyl-4-(3-phenyl-2*H*-indazol-2-yl)benzamide (**3h**). Pale brown solid (70% yield); m.p.:153.0–154.5 °C. ^1^H NMR (600 MHz, DMSO-*d*_6_) δ 7.75 (d, *J* = 8.7 Hz, 1H), 7.68 (d, *J* = 8.5 Hz, 1H), 7.53–7.35 (m, 10H), 7.20–7.14 (m, 1H), 2.99 (s, 3H), 2.91 (s, 3H); ^13^C NMR (151 MHz, DMSO-*d*_6_) δ 169.52, 148.78, 140.70, 136.78, 135.56, 129.95, 129.48, 129.43, 129.08, 128.23, 127.53, 126.32, 123.12, 121.67, 120.82, 117.89, 35.18; MS [M+H]^+^ *m*/*z* 342.24.

*N-*Methyl-4-(2-phenyl-2*H*-indazol-3-yl)benzamide (**3n**). Pale brown solid (62% yield); m.p.: 235.7–236.6 °C; ^1^H NMR (600 MHz, DMSO-*d*_6_) δ 8.52 (d, *J* = 4.5 Hz, 1H), 7.89–7.86 (m, 2H), 7.79–7.75 (m, 1H), 7.72 (dd, *J* = 8.5, 1.0 Hz, 1H), 7.51–7.44 (m, 7H), 7.40 (ddd, *J* = 8.6, 6.6, 0.9 Hz, 1H), 7.23–7.17 (m, 1H), 2.79 (d, *J* = 4.6 Hz, 3H); ^13^C NMR (151 MHz, DMSO-*d*_6_) 166.40, 148.69, 140.16, 134.62. 134.54, 132.10, 129.76, 129.63, 129.14, 127.98, 127.41, 126.56, 123.29, 121.69, 120.67, 117.97, 26.72; MS [M+H]^+^ *m*/*z* 328.1442.

*N,N*-Dimethyl-4-(2-phenyl-2*H*-indazol-3-yl)benzamide (**3o**). White solid (82% yield); m.p.: 166.2–168.0 °C; ^1^H NMR (600 MHz, DMSO-*d*_6_) 7.76 (dt, *J* = 8.7, 0.7 Hz, 1H), 7.72 (dt, *J* = 8.5, 0.8 Hz, 1H), 7.51–7.44 (m, 7H), 7.43–7.38 (m, 3H), 7.19 (ddd, *J* = 8.5, 6.6, 0.7 Hz, 1H), 2.99 (s, 3H), 2.92 (s, 3H); ^13^C NMR (151 MHz, DMSO-*d*_6_) 174.63, 153.43, 144.91, 141.38, 139.46, 135.24, 134.48, 134.37, 133.89, 132.67, 132.15, 131.28, 127.94, 126.42, 125.46, 122.70, 39.94; MS [M+H]^+^ *m*/*z* 342.21.

#### 3.2.8. Synthesis of Pyrazole Derivatives (**6a**, **6e**, and **6h**)

To a solution of 1,3-dicarbonyl compound (2 mmol) in 5 mL of a 5% methanolic solution of H_2_SO_4_, hydrazine hydrate or 4-hydrazinylbenzoic acid (2 mmol) was added. The reaction mixture was stirred at room temperature for 2–5 h and then cooled on an ice bath. To induce a complete precipitation, water (2–5 mL) was added. The resulting solid was separated in vacuo and dried [21].

4-(3-Methyl-5-phenyl-1*H*-pyrazol-1-yl)benzoic acid (**6a**). White solid (91% yield); m.p.: 126.3–128.3 °C; the spectroscopic data matched previously reported data [27] ^1^H NMR (600 MHz, CDCl_3_) δ 8.06–8.01 (m, 2H), 7.40–7.36 (m, 2H), 7.36–7.31 (m, 3H), 7.25–7.20 (m, 2H), 6.34 (s, 1H), 3.49 (s, 1H), 2.41 (s, 3H); ^13^C NMR (151 MHz CDCl_3_) δ 170.56, 150.50, 144.12, 144.10, 130.96, 130.43, 128.73, 128.66, 128.56, 127.58, 124.29, 109.03, 13.53; MS [M+H]^+^ *m*/*z* 279.17.

4-(3,5-Dimethyl-1*H*-pyrazol-1-yl)benzoic acid (**6e**). White solid (90% yield); m.p.: 160.7–161.8 °C; the spectroscopic data matched previously reported data [28]: ^1^H NMR (600 MHz, CDCl_3_) 8.19 (d, *J* = 8.7 Hz, 2H), 7.59 (d, *J* = 8.7 Hz, 2H), 6.05 (s, 1H), 2.39 (d, *J* = 0.6 Hz, 3H), 2.33 (s, 3H); RMN ^13^C (151 MHz, CDCl_3_) δ 170.33, 150.06, 143.94, 139.73, 131.15, 127.74; MS [M+H]^+^ *m*/*z* 217.10.

Methyl 4-(3-methyl-1*H*-pyrazol-5-yl)benzoate (**6h**). Yellow solid (82% yield); m.p.: 188.0–191 °C; ^1^H NMR (600 MHz, DMSO-*d*_6_) δ 12.80 (bs, 1H), 7.97 (d, *J* = 8.5 Hz, 2H), 7.90 (d, *J* = 8.4 Hz, 2H), 6.56 (s, 1H), 3.86 (s, 3H), 2.27 (s, 3H); ^13^C NMR (151 MHz, DMSO-*d*_6_) δ 166.48, 130.06, 128.42, 125.38, 125.09, 102.41, 52.47, 11.22; MS [M+H]^+^ *m*/*z* 217.10.

#### 3.2.9. Pyrazole Derivatives (**6b** and **6f**)

A solution of carboxylic acid (**6a** or **6e**, 1.1 mmol) in 5 mL of a 5% methanolic solution of H_2_SO_4_ was heated under reflux for 2 h. When the reaction was completed, the mixture was neutralized with a 10% aqueous solution of NaHCO_3_. The resulting solid was separated under vacuum filtration and dried.

Methyl 4-(3-methyl-5-phenyl-1*H*-pyrazol-1-yl)benzoate (**6b**). White solid (84% yield); m.p.: 100.2–101.6 °C; ^1^H NMR (600 MHz, CDCl_3_) δ 7.97 (d, *J* = 8.8 Hz, 2H), 7.37–7.33 (m, 2H), 7.33–7.29 (m, 3H), 7.24–7.19 (m, 2H), 6.33 (s, 1H), 3.90 (s, 3H), 2.39 (s, 3H). ^13^C NMR (151 MHz, CDCl_3_) δ 166.38, 150.28, 143.95, 143.63, 130.51, 130.29, 128.67, 128.56, 128.41, 128.18, 124.19, 108.84, 52.14, 13.56; MS [M+H]^+^ *m*/*z* 293.19.

Methyl 4-(3,5-dimethyl-1*H*-pyrazol-1-yl)benzoate (**6f**). White solid (73% yield); m.p.: 60.2–61.2 °C; ^1^H NMR (600 MHz, CDCl_3_) 8.12 (d, *J* = 8.8 Hz, 2H), 7.55 (d, *J* = 8.8 Hz, 2H), 6.03 (s, 1H), 3.94 (s, 3H), 2.37 (d, *J* = 0.6 Hz, 3H), 2.30 (s, 3H); ^13^C NMR (151 MHz, CDCl_3_) 166.38, 149.87, 143.61, 139.53, 130.52, 128.25, 123.54, 108.16, 52.19, 13.48, 12.78; MS [M+H]^+^ *m*/*z* 231.13.

#### 3.2.10. 4-(3-Methyl-1*H*-pyrazol-5-yl) Benzoic Acid (**6g**)

Methyl 4-(3-methyl-1*H*-pyrazol-5-yl) benzoate **6h** (250 mg, 1.1 mmol) was dissolved in a 5 mL of a 60% aqueous solution of H_2_SO_4_ and heated under reflux for 2 h. After completion, the reaction was poured into 10 mL of iced water. The resulting solid was filtered in vacuo, washed with cold water and dried.

4-(3-Methyl-1*H*-pyrazol-5-yl)benzoic acid (**6g**). Yellow solid (95% yield); m.p.: 285–286 °C; ^1^H NMR (600 MHz, DMSO-*d*_6_) δ 7.96 (d, *J* = 8.5 Hz, 2H), 7.88 (d, *J* = 8.5 Hz, 2H), 6.55 (d, *J* = 0.7 Hz, 1H), 2.29–2.26 (m, 3H); ^13^C NMR (151 MHz, DMSO-*d*_6_) δ 167.55, 148.45, 141.87, 137.76, 130.20, 129.65, 125.27, 102.40, 11.40; MS [M+H]^+^ *m*/*z* 203.13.

#### 3.2.11. 4-(3-Methyl-1-phenyl-1*H*-pyrazol-5-yl) Benzoic Acid (**6c**)

5-Methyl-2-phenyl-2,4-dihydro-3*H*-pyrazol-3-one (1.0 g, 5.74 mmol) was dissolved in POCl_3_ (1.5 mL) and heated under reflux for 5 h. The mixture was cooled on an ice bath and quenched with 10 mL of water. The product was extracted with ethyl acetate (3 × 10 mL), the combined organic layers were dried over Na_2_SO_4_ and concentrated in vacuo. The resulting oily product (200 mg, 1.0 mmol) was mixed with 4-carbomethoxyphenylboronic acid pinacol ester (300 mg, 1.1 mmol), Pd(PPh_3_)_4_ (116 mg, 0.1 mmol), Na_2_CO_3_ (424 mg, 4 mmol) and 5 mL of MeCN/H_2_O (4:1). The reaction was heated under microwave irradiation at 175 °C for 20 min twice. Then, the solvent was removed in vacuo. The resulting crude product was purified by column chromatography using hexane/ethyl acetate (80:20).

4-(3-Methyl-1-phenyl-1*H*-pyrazol-5-yl)benzoic acid (**6c**). Pale brown solid (55% yield); m.p.: 221.0–223.3 °C; ^1^H NMR (600 MHz, CDCl_3_) δ 8.05–7.97 (m, 2H), 7.37–7.32 (m, 2H), 7.32–7.28 (m, 3H), 7.27 (dd, *J* = 8.4, 1.4 Hz, 2H), 6.41 (s, 1H), 2.41 (s, 3H).; ^13^C NMR (151 MHz, CDCl_3_) δ 170.85, 149.72, 142.48, 139.73, 135.69, 130.23, 129.04, 128.77, 128.47, 127.56, 125.24, 108.41, 13.46; MS [M+H]^+^ *m*/*z* 279.17.

#### 3.2.12. Methyl 4-(3-Methyl-1-phenyl-1*H*-pyrazol-5-yl) Benzoate (**6d**)

A mixture of 4-(3-methyl-1-phenyl-1*H*-pyrazol-5-yl) benzoic acid **6c** (200 mg, 0.7 mmol) and Na_2_CO_3_ (75 mg, 0.7 mmol), DMF (2 mL) and water (0.5 mL) was stirred for 15 min at room temperature. Afterward, methyl iodide (0.7 mmol) was added to the mixture and stirred at room temperature for 1 h. Then, the reaction was poured into water (10 mL) and extracted with ethyl acetate (3 × 5 mL). The combined organic layers were dried over Na_2_SO_4_ and concentrated under vacuum. The crude product was purified by recrystallization from ethanol.

Methyl 4-(3-methyl-1-phenyl-1*H*-pyrazol-5-yl)benzoate (**6d**). White solid (82% yield); 92.0–94.3 °C; the spectroscopic data matched previously reported data [29] ^1^H NMR (600 MHz, CDCl_3_) 7.95 (d, *J* = 8.6 Hz, 2H), 7.36–7.31 (m, 2H), 7.31–7.23 (m, 5H), 6.39 (s, 1H), 3.91 (s, 3H), 2.39 (s, 3H); ^13^C NMR (151 MHz, CDCl_3_) 166.57, 149.64, 142.54, 139.87, 135.02, 129.63, 129.47, 128.99, 128.40, 127.42, 125.17, 108.28, 52.18, 13.52; MS [M+H]^+^ *m*/*z* 293.15.

#### 3.2.13. Synthesis of 3-Phenyl-1*H*-Indazoles (**10a**, **10b**, and **10d–i**)

A mixture of 3-bromo-1*H*-indazole (200 mg, 1.01 mmol), phenylboronic acid or appropriate aryl boronic acid pinacol ester (1.11 mmol), Na_2_CO_3_ (128 mg, 1.21 mmol), PPh_3_ (8 mg, 0.03 mmol), Pd(OAc)_2_ (2.2 mg, 0.01 mmol) and 5 mL of *n*-propanol/H_2_O (3:1) was heated under microwave irradiation at 150 °C for 20 min. The solvent was removed in vacuo and the resulting residue was purified by column chromatography using hexane/ethyl acetate (50:50).

3-Phenyl-1*H*-indazole (**10a**). The product was purified using column chromatography and hexane/ethyl acetate (80:20) as mobile phase. White solid (80% yield). m.p.: 105.2–106.8 °C [lit. [30]: 106–107 °C]; the spectroscopic data matched previously reported data [31,32]: ^1^H NMR (600 MHz, CDCl_3_) δ 11.44 (bs, 1H), 8.05–7.99 (m, 3H), 7.56–7.51 (m, 2H), 7.47–7.42 (m, 1H), 7.37–7.33 (m, 1H), 7.29–7.25 (m, 1H), 7.23–7.19 (m, 1H); ^13^C NMR (151 MHz, CDCl_3_) δ 145.74, 141.68, 133.56, 128.92, 128.16, 127.71, 126.77, 121.33, 121.08, 120.95, 110.21; Purity (qNMR, % *w*/*w*): 98.89 ± 2.29.

4-(1*H*-Indazol-3-yl)benzoic acid (**10b**). White solid (82% yield); m.p.: 283.5–286 °C; the spectroscopic data matched previously reported data [32]: ^1^H NMR (600 MHz, DMSO-*d*_6_) δ 13.52 (bs, 1H), 13.05 (bs, 1H), 8.19–8.14 (m, 3H), 8.12–8.08 (m, 2H), 7.64 (d, *J* = 8.4 Hz, 1H), 7.48–7.42 (m, 1H), 7.26 (ddd, *J* = 7.8, 6.8, 0.8 Hz, 1H); ^13^C NMR (151 MHz, DMSO-*d*_6_) δ 172.33, 148.26, 147.20, 146.82, 143.12, 135.17, 134.68, 131.70, 131.48, 126.67, 125.81, 125.76, 125.28, 116.01; Purity (qNMR, % *w*/*w*): 96.48 ± 0.42.

4-(1*H*-Indazol-3-yl)benzamide (**10d**). Pale brown solid (45% yield); m.p.: 253.0–255.5 °C; ^1^H NMR (600 MHz, DMSO-*d*_6_) δ 13.43 (s, 1H), 8.14 (d, *J* = 8.3 Hz, 1H), 8.12–8.09 (m, 3H), 8.04 (d, *J* = 8.5 Hz, 2H), 7.63 (d, *J* = 8.4 Hz, 1H), 7.49–7.41 (m, 2H), 7.25 (ddd, *J* = 7.9, 6.9, 0.7 Hz, 1H); ^13^C NMR (151 MHz, DMSO-*d*_6_) δ 168.04, 142.74, 142.03, 136.86, 133.54, 128.59, 126.71, 126.68, 121.80, 121.08, 120.52, 111.16; MS [M+H]^+^ *m*/*z* 238.14; Purity (qNMR, % *w*/*w*): 95.23±0.71.

4-(1*H*-Indazol-3-yl)-*N*-methylbenzamide (**10e**). White solid (34% yield); m.p.: 239.5–241.0 °C; ^1^H NMR (600 MHz, DMSO-*d*_6_) δ 13.41 (s, 1H), 8.55 (d, *J* = 4.5 Hz, 1H), 8.14 (d, *J* = 8.2 Hz, 1H), 8.10 (d, *J* = 8.4 Hz, 2H), 7.99 (d, *J* = 8.4 Hz, 2H), 7.62 (d, *J* = 8.4 Hz, 1H), 7.47–7.41 (m, 1H), 7.28–7.22 (m, 1H), 3.38 (s, 3H); ^13^C NMR (151 MHz DMSO-*d*_6_) δ 166.17, 142.17, 141.47, 136.12, 133.22, 127.59, 126.22, 126.13, 121.23, 120.54, 119.96, 110.60, 26.19; MS [M+H]^+^ *m*/*z* 252.17; Purity (qNMR, % *w*/*w*): 96.59 ± 0.80.

4-(1*H*-Indazol-3-yl)-*N,N*-dimethylbenzamide (**10f**). White solid (48% yield); m.p.: 160.5–163.0 °C; the spectroscopic data matched previously reported data [12]: ^1^H NMR (600 MHz, DMSO-*d*_6_) δ 13.38 (s, 1H), 8.12 (d, *J* = 8.3 Hz, 1H), 8.07 (d, *J* = 8.3 Hz, 2H), 7.62 (d, *J* = 8.4 Hz, 1H), 7.56 (d, *J* = 8.3 Hz, 2H), 7.46–7.40 (m, 1H), 7.24 (ddd, *J* = 7.9, 6.8, 0.8 Hz, 1H), 3.01 (d, *J* = 16.2 Hz, 6H); ^13^C NMR (151 MHz, DMSO-*d*_6_) δ 169.76, 142.30, 141.47, 135.30, 134.58, 127.60, 126.27, 126.12, 121.16, 120.47, 119.92, 110.60, 34.71; MS [M+H]^+^ *m*/*z*: 266.20; Purity (qNMR, % *w*/*w*): 97.56 ± 1.07.

*N,N*-Diethyl-4-(1*H*-indazol-3-yl)benzamide (**10g**). White solid (53% yield); m.p.: 259.3–261.5 °C; ^1^H NMR (600 MHz, DMSO-*d*_6_) δ 13.37 (s, 1H), 8.12 (d, *J* = 8.2 Hz, 1H), 8.07 (d, *J* = 8.3 Hz, 2H), 7.62 (d, *J* = 8.4 Hz, 1H), 7.50 (d, *J* = 8.3 Hz, 2H), 7.46–7.40 (m, 1H), 7.27–7.21 (m, 1H), 3.46 (s, 2H), 3.27 (s, 2H), 1.16 (bs,3H), 1.10 (bs, 3H); ^13^C NMR (151 MHz, DMSO-*d*_6_) δ 169.62, 142.31, 141.47, 136.17, 134.25, 126.71, 126.42, 126.11, 121.14, 120.47, 119.90, 110.59, 42.78, 13.98, 12.74; MS [M+H]^+^ *m*/*z* 294.23; Purity (qNMR, % *w*/*w*): 96.68 ± 0.33.

(4-(1*H*-Indazol-3-yl)phenyl)(pyrrolidin-1-yl)methanone (**10h**). White solid (21% yield); m.p.: 201.0–202.3 °C; ^1^H NMR (600 MHz, DMSO-*d*_6_) δ 13.39 (s, 1H), 8.12 (d, *J* = 8.3 Hz, 1H), 8.07 (dd, *J* = 6.6, 1.8 Hz, 2H), 7.70–7.66 (m, 2H), 7.62 (d, *J* = 8.4 Hz, 1H), 7.46–7.40 (m, 1H), 7.24 (ddd, *J* = 7.9, 6.9, 0.8 Hz, 1H), 3.52–3.46 (m, 4H), 1.91–1.81 (m, 4H); ^13^C NMR (151 MHz DMSO-*d*_6_) δ 167.79, 142.28, 141.47, 135.97, 135.04, 134.91, 127.70, 126.17, 126.11, 121.18, 120.46, 119.93, 110.60, 48.88, 45.91, 25.93, 23.82; MS [M+H]^+^ *m*/*z* 292.20; Purity (qNMR, % *w*/*w*): 99.12 ± 1.25.

(4-(1*H*-indazol-3-yl)phenyl)(piperidin-1-yl)methanone (**10i**). White solid (42% yield); m.p.: 193.5–195.5 °C; ^1^H NMR (600 MHz, DMSO-*d*_6_) δ 13.38 (s, 1H), 8.12 (d, *J* = 8.2 Hz, 1H), 8.07 (d, *J* = 8.2 Hz, 2H), 7.62 (d, *J* = 8.6 Hz, 1H), 7.52 (d, *J* = 8.2 Hz, 2H), 7.46–7.39 (m, 1H), 7.27–7.20 (m, 1H), 3.61 (bs, 2H), 3.36 (bs, 2H), 1.65–14.5 (m, 6H); ^13^C NMR (151 MHz, DMSO-*d*_6_) δ 169.08, 142.84, 142.00, 135.86, 135.06, 127.83, 126.94, 126.66, 121.69, 121.01, 120.45, 111.13, 48.56, 42.84, 26.47, 25.74, 24.51; MS [M+H]^+^ *m*/*z* 306.23; Purity (qNMR, % *w*/*w*): 97.32 ± 0.62.

#### 3.2.14. Methyl 4-(1*H*-Indazol-3-yl) Benzoate (**10c**)

Employing the esterification method 3.2.9. described above, compound **10c** was synthesized from acid derivative **10b**.

Methyl 4-(1*H*-indazol-3-yl)benzoate (**10c**). Yellow solid (94% yield); m.p.: 205.5–208.0 °C; the spectroscopic data matched previously reported data [16]: ^1^H NMR (600 MHz, DMSO-*d*_6_) δ 13.52 (s, 1H), 8.19 (d, *J* = 8.4 Hz, 2H), 8.16 (d, *J* = 8.1 Hz, 1H), 8.11 (d, *J* = 8.4 Hz, 2H), 7.64 (d, *J* = 8.4 Hz, 1H), 7.45 (dd, *J* = 11.3, 3.8 Hz, 1H), 7.27 (dd, *J* = 11.2, 3.9 Hz, 1H), 3.90 (s, 3H); ^13^C NMR (151 MHz DMSO-*d*_6_) δ 165.95, 141.78, 141.53, 138.24, 129.73, 128.20, 126.54, 126.24, 121.46, 120.47, 120.00, 110.74, 52.07; Purity (qNMR, % *w*/*w*): 96.76 ± 1.06.

### 3.3. Anticandidal Activity Assays

#### 3.3.1. Agar Diffusion Method

Susceptibility assays were carried out using the cylinder-plate method (agar diffusion method) described in the general methods of analysis MGA 0100 of the Mexican Pharmacopeia [23]. *Candida* strains from the American Type Culture Collection (ATCC) were used: *Candida albicans* (18,804), *Candida glabrata* (90,030, susceptible to miconazole), *Candida glabrata* (32,554, resistant to miconazole), and *Candida tropicalis* (750). Compounds tested, fluconazole and miconazole were properly weighed and solubilized in dimethyl sulfoxide (DMSO) at 100%. The microorganisms were grown and maintained on Sabouraud dextrose broth (SB, Bioxon, Mexico) for 24 h at 35 ± 2 °C. A base layer was prepared into Petri dishes (100 × 20 mm) employing 10 mL of a mixture of agar SB with *Candida* suspension adjusted to 0.5 McFarland standard. After solidification, a second layer was formed by the addition of 10 mL of agar SB. In each plate, five stainless steel cylinders of uniform size (8 × 6 × 10 mm) were placed on the surface and filled with 100 µL of a solution of the compounds at 10, 1 and 0.1 mM. Fluconazole (1 mM) and miconazole (1 mM) were used as positive controls, and vehicle as a negative control. The plates were incubated at 35 ± 2 °C for 24 h. The degree of effectiveness was measured by determining the zone of inhibition in millimeters produced by the tested compounds and considering the effect produced by DMSO.

#### 3.3.2. Determination of Minimum Inhibitory Concentration (MIC) of the Most Effective Compounds

Minimum inhibitory concentration is defined as the lowest concentration of compounds against *Candida* strains that inhibit their growth after 24 h of incubation. The 3-phenyl-1*H*-indazole derivatives, which showed antimicrobial activity at the least concentration tested (100 µM), were evaluated against *Candida albicans* (18,804), *Candida glabrata* (90,030, susceptible to miconazole), *Candida glabrata* (32,554, resistant to miconazole). The cylinder-plate method and different concentrations of tested compounds (50, 75, 100, 125, 150, and 200 µM) were used for the assay. Fluconazole and miconazole were used as positive controls and vehicle (DMSO) as negative control. Experiments were carried out in duplicate and repeated three times.

### 3.4. Cytotoxicity Assays

HeLa (human cervix), K562 (human chronic myelogenous leukemia), and SW620 (human colon) cells were grown in DMEM (Invitrogen Corporation, Carlsbad, CA, USA) supplemented with 10% FBS (BioWest, Riverside, MO, USA) and maintained in standard culture conditions (37 °C, 95% humidity, and 5% CO_2_). Cells were grown to a density of 80% and then were harvested using sterile PBS/EDTA (pH 7.4) before starting every experiment. Cells were seeded in 96-well plates (7000 cells/well in 200 µL of DMEM). After 24 h the cells were exposed to test compounds dissolved in 0.5 % DMSO and diluted with DMEM at 50 µM, to reach 250 µL in the well. The exposure time was 48 h, and then viability was determined by MTT assay. The absorbance of formazan was determined for each well and its viability was related to the vehicle (100%) [17].

### 3.5. Quantitative Nuclear Magnetic Resonance Spectroscopy

The purity of compounds **10a–i** was determined by qNMR using sodium 2,2-dimethyl-2-silapentane-5-sulfonate (DSS, 97%, CIL) as an internal standard. The 4-bromobenzonitrile (99%, Sigma-Aldrich) was used for the validation of the method. As solvent deuterated dimethyl sulfoxide-d6 (DMSO-*d_6_*, >99.96%, CIL) was used.

Samples were prepared as a mixture of compounds tested and DSS was dissolved in DMSO-*d_6_*. The measurements were carried out with an Agilent DD2 spectrometer operating at 600 MHz. In general, the experiments were acquired employing the parameters: 90° pulse, an acquisition time of 7 s, relaxation delay of 60 s, digital resolution of 0.8 Hz, a spectral window of 15 ppm, and a total of 64 scans. Phase and baseline corrections were done automatically using the software MestReNova v.6.0.2. The signal integration was done in automatic mode. For determination of purity were used separated signals (H-5, H-6, or NH) in the aromatic region and three signals of DSS (CH_2_) in the aliphatic region. The percentage of purity was calculated by Equation (1):

(1) $$P_{Sample}=\;\frac{I_{Sample}}{I_{Std}}\times \frac{N_{Std}}{N_{Sample}}\times \frac{M_{Sample}}{M_{Std}}\times \frac{m_{Std}}{m_{Sample}}\times P_{Std}$$

where *I* is the integrated area, *N* is the number of spins, *M* is the molar mass, *m* is the gravimetric weight, and P is the purity in % *w*/*w* [33,34].

## 4. Conclusions

In the present study, several indazole and pyrazole derivatives were designed and synthesized by bioisosteric replacement, homologation, and molecular simplification. Besides, a new series of derivatives was designed by combining the 3-phenyl-1*H*-indazole moiety with carboxamide substituents. Taking the activity data from the series of the 3-phenyl-1*H*-indazole moiety, compounds **10c**, **10g**, and **10i** had the best effect against *C. albicans.* In comparison, **10f** and **10g** were active against both miconazole susceptible and resistant *C. glabrata* strains. In particular, compound **10g** was demonstrated to have the best anticandidal activity against *Candida* strains tested. According to the data, the molecular simplification along with *p*-carbonyl substituents was crucial for the anticandidal activity. Therefore, the 3-phenyl-1*H*-indazole nucleus is a new scaffold for anticandidal agents that has not been reported to date. In light of these results, new research could be conducted to explore other substituents attached to the 3-phenyl group to generate more information about the structural requirements needed to improve the anticandidal activity.

## Figures and Tables

**Table 1:** In vitro growth inhibition of 2,3-diphenyl-2*H*-indazoles, 2-phenyl-2*H*-indazole, 3,5-1*H*-pyrazole, and 3-phenyl-1*H*-indazole derivatives against *Candida* species.

ID	R ^1^	R ^2^	*C. albicans*			*C. glabrata*			*C. glabrata* ^1^			*C. tropicalis*
			[ ] mM									
			10	1	0.1	10	1	0.1	10	1	0.1	10
**3a**	CO_2_Me	H	+	–	–	–	–	–	–	–	–	–
**3b**	CO_2_H	H	+	–	–	+	–	–	+	–	–	+ +
**3c**	H	CO_2_Me	–	–	–	–	–	–	–	–	–	–
**3d**	H	CO_2_H	+	–	–	–	–	–	+	–	–	–
**3e**	NHCO_2_Me	H	–	–	–	–	–	–	–	–	–	+ +
**3f**	CONH_2_	H	–	–	–	–	–	–	–	–	–	+ +
**3g**	CONHMe	H	–	–	–	+	–	–	–	–	–	–
**3h**	CONMe_2_	H	+	+	–	–	–	–	+	+	–	–
**3i**	CONEt_2_	H	+	+	–	–	–	–	–	–	–	–
**3j**	CONC_4_H_8_	H	+ +	+	–	–	–	–	+	–	–	–
**3k**	CONC_5_H_10_	H	+	+	–	–	–	–	–	–	–	–
**3l**	H	NHCO_2_Me	–	–	–	+	–	–	+	–	–	–
**3m**	H	CONH_2_	+	–	–	+	–	–	–	–	–	–
**3n**	H	CONHMe	+	+	–	–	–	–	–	–	–	–
**3o**	H	CONMe_2_	+	+	–	–	–	–	–	–	–	–
**3p**	H	CONEt_2_	+	+	–	–	–	–	+	+	–	–
**3q**	H	CONC_4_H_8_	+	+	–	–	–	–	–	–	–	–
**3r**	H	CONC_5_H_10_	+	+	–	–	–	–	+	–	–	–
**6a**	Ph	4-(CO_2_H)Ph	–	–	–	–	–	–	+	+	–	–
**6b**	Ph	4-(CO_2_Me)Ph	+	+	–	+	–	–	+	+	–	–
**6c**	4-(CO_2_H)Ph	Ph	+	+	–	–	–	–	+	+	–	–
**6d**	4-(CO_2_Me)Ph	Ph	+	+	–	–	–	–	+	+	–	–
**6e**	Me	4-(CO_2_H)Ph	–	–	–	–	–	–	+	+	–	–
**6f**	Me	4-(CO_2_Me)Ph	+	+	–	+	+	–	+	+	–	+ +
**6g**	4-(CO_2_H)Ph	H	+	+	–	–	–	–	–	–	–	–
**6h**	4-(CO_2_Me)Ph	H	+	+	–	–	–	–	–	–	–	–
**2a**	H	–	+	+	–	–	–	–	–	–	–	–
**2b**	CO_2_H	–	+	+	+	+ ^2^	+ +	–	–	–	–	–
**2c**	CO_2_Me	–	–	+	–	+	–	–	+ +	+ +	–	–
**10a**	H	–	+ + +	+ +	+	+ +	+	–	+ + +	+ +	+	–
**10b**	CO_2_H	–	+ +	+ +	–	+	+	+	–	–	–	–
**10c**	CO_2_Me	–	+ + +	+ + +	+ +	+ +	+	+	– ^2^	+ +	–	–
**V ^3^**				–			–			–		–
**M ^4^**				+ + +			+ + +			+		++
**F ^5^**				+ +			–			+ + +		+

^1^ Miconazole resistant strain; ^2^ compound precipitation was observed at 10 mM. (–) inactive (<12 mm); (+) weakly active (13–16 mm); (+ +) moderately active (17–21 mm); (+ + +) strongly active (22–29 mm). ^3^ Vehicle; ^4^ Miconazole; ^5^ Fluconazole.

**Table 2:** Minimum Inhibitory Concentration (µM) of 3-phenyl-1*H*-indazole derivatives.

ID	R	*C. albicans*	*C. glabrata*	*C. glabrata* ^1^
**10a**	H	100	150	100
**10b**	CO_2_H	200	100	–
**10c**	CO_2_Me	75	100	200
**10d**	CONH_2_	150	150	100
**10e**	CONHMe	150	100	100
**10f**	CONMe_2_	150	75	75
**10g**	CONEt_2_	75	100	75
**10h**	CONC_4_H_8_	100	100	150
**10i**	CONC_5_H_10_	75	100	125
Fluconazole		50	–	50
Miconazole		<50	<50	–

^1^ Miconazole resistant; (–) inactive.

## Data Availability

The data presented in this study are available in supplementary material.

## Supplementary Materials

The following are available online at https://www.mdpi.com/1424-8247/14/3/176/s1, Figure S1: Determination of the inhibition zone for compound 2b tested at 10, 1, and 0.1 mM against *C. albicans*, Figures S2–S41: ^1^H NMR and ^13^C NMR spectra of compounds 2a–e, 3a–r, 6a–h and 10a–i, Figures S42–S71: MS spectra of compounds 2d, 2e, 3e–r, 6a–h and 10d–i, Table S1: Percentage of purity of compounds 10a–i by qNMR used an internal standard and Figure S72: Spectra of qNMR for 4-bromobenzonitrile (purity > 99%) as a reference compound.

## References

[1] Brown G.D., Denning D.W., Gow N.A.R., Levitz S.M., Netea M.G., White T.C. (2012). Hidden Killers: Human Fungal Infections. Sci. Transl. Med..

[2] Bongomin F., Gago S., Oladele R.O., Denning D.W. (2017). Global and Multi-National Prevalence of Fungal Diseases—Estimate Precision. J. Fungi.

[3] Gonçalves B., Ferreira C., Alves C.T., Henriques M., Azeredo J., Silva S. (2015). Vulvovaginal candidiasis: Epidemiology, microbiology and risk factors. Crit. Rev. Microbiol..

[4] Centers for Disease Control and Prevention (2015). Sexually transmitted diseases treatment guidelines, 2015. MMWR. Recommen-dations and reports: Morbidity and mortality weekly report. Recomm. Rep..

[5] Kullberg B.J., Arendrup M.C. (2015). Invasive Candidiasis. N. Engl. J. Med..

[6] Pfaller M.A., Diekema D.J. (2007). Epidemiology of Invasive Candidiasis: A Persistent Public Health Problem. Clin. Microbiol. Rev..

[7] Centers for Disease Control and Prevention Invasive Candidiasis. https://www.cdc.gov/fungal/diseases/candidiasis/invasive/statistics.html.

[8] Fisher M.C., Hawkins N.J., Sanglard D., Gurr S.J. (2018). Worldwide emergence of resistance to antifungal drugs challenges human health and food security. Science.

[9] Pappas P.G., Kauffman C.A., Andes D.R., Clancy C.J., Marr K.A., Ostrosky-Zeichner L., Reboli A.C., Schuster M.G., Vazquez J.A., Walsh T.J. (2016). Clinical Practice Guideline for the Management of Candidiasis: 2016 Update by the Infectious Diseases Society of America. Clin. Infect. Dis..

[10] Thangadurai A., Minu M., Wakode S., Agrawal S., Narasimhan B. (2012). Indazole: A medicinally important heterocyclic moiety. Med. Chem. Res..

[11] Gaikwad D.D., Chapolikar A.D., Devkate C.G., Warad K.D., Tayade A.P., Pawar R.P., Domb A.J. (2015). Synthesis of indazole motifs and their medicinal importance: An overview. Eur. J. Med. Chem..

[12] Reddy G., Ivaturi K., Allaka T. (2019). Design, Synthesis and Docking Studies of New Indazole Derivatives as Potent Cytotoxic and Antibacterial Agents. Indian J. Heterocycl. Chem..

[13] Lipunova G.N., Nosova E.V., Charushin V.N., Chupakhin O.N. (2016). Fluorine-containing indazoles: Synthesis and biological activity. J. Fluor. Chem..

[14] Vega M.C., Rolón M., Montero-Torres A., Fonseca-Berzal C., Escario J.A., Gómez-Barrio A., Gálvez J., Marrero-Ponce Y., Arán V.J. (2012). Synthesis, biological evaluation and chemometric analysis of indazole derivatives. 1,2-Disubstituted 5-nitroindazolinones, new prototypes of antichagasic drug. Eur. J. Med. Chem..

[15] Park J.S., A Yu K., Kang T.H., Kim S., Suh Y.-G. (2007). Discovery of novel indazole-linked triazoles as antifungal agents. Bioorganic Med. Chem. Lett..

[16] Chen H.-S., Kuo S.-C., Teng C.-M., Lee F.-Y., Wang J.-P., Lee Y.-C., Kuo C.-W., Huang C.-C., Wu C.-C., Huang L.-J. (2008). Synthesis and antiplatelet activity of ethyl 4-(1-benzyl-1H-indazol-3-yl)benzoate (YD-3) derivatives. Bioorganic Med. Chem..

[17] Pérez-Villanueva J., Yépez-Mulia L., González-Sánchez I., Palacios-Espinosa J.F., Soria-Arteche O., Sainz-Espuñes T.R., Cerbón M.A., Rodríguez-Villar K., Rodríguez-Vicente A.K., Cortés-Gines M. (2017). Synthesis and Biological Evaluation of 2H-Indazole Derivatives: Towards Antimicrobial and Anti-Inflammatory Dual Agents. Molecules.

[18] (1968). 2-PHENYLINDAZOLE. Org. Synth..

[19] Ohnmacht S.A., Culshaw A.J., Greaney M.F. (2010). Direct Arylations of 2H-Indazoles On Water. Org. Lett..

[20] Kümmerle A.E., Schmitt M., Cardozo S.V.S., Lugnier C., Villa P., Lopes A.B., Romeiro N.C., Justiniano H., Martins M.A., Fraga C.A.M. (2012). Design, Synthesis, and Pharmacological Evaluation ofN-Acylhydrazones and Novel Conformationally Constrained Compounds as Selective and Potent Orally Active Phosphodiesterase-4 Inhibitors. J. Med. Chem..

[21] Wang Z.-X., Qin H.-L. (2004). Solventless syntheses of pyrazole derivativesElectronic supplementary information (ESI) available: Analytical and spectroscopic data. Green Chem..

[22] Huff B.E., Koenig T.M., Mitchell D., Staszak M.A. (2003). Synthesis of Unsymmetrical Biaryls Using a Modified Suzuki Cross-Coupling: 4-Biphenylcarboxaldehyde. Org. Synth..

[23] Secretaria de Salud, Comisión permanente de la Farmacopea de los Estados Unidos Mexicanos (2018). Valoración microbiológica de antibióticos, MGA 0100. Farmacopea de los Estados Unidos Mexicanos FEUM.

[24] Haag B., Peng Z., Knochel P. (2009). Preparation of Polyfunctional Indazoles and Heteroarylazo Compounds Using Highly Functionalized Zinc Reagents. Org. Lett..

[25] Zhou D., Sze J.Y.-C., Gross J.L., Robichaud A.J. (2008). Preparation of Azacyclylbenzamide Derivatives as Histamine-3 Antago-nists for Treating CNS Disorders. U.S. Patent.

[26] Geng X., Wang C. (2015). Rhenium-Catalyzed [4 + 1] Annulation of Azobenzenes and Aldehydes via Isolable Cyclic Rhenium(I) Complexes. Org. Lett..

[27] Vickerstaffe E. (2004). The Development and Application of Automated Multi-Step Polymer Assisted Solution Phase Synthesis for the Preparation of Biologically Active Compound Arrays. Ph.D. Thesis.

[28] Porcelloni M., D’Andrea P., Rossi C., Sisto A., Ettorre A., Madami A., Altamura M., Giuliani S., Meini S., Fattori D. (2008). α,α-Cyclopentaneglycine Dipeptides Capped with Biaryls as Tachykinin NK2Receptor Antagonists. ChemMedChem.

[29] Sar D., Bag R., Yashmeen A., Bag S.S., Punniyamurthy T. (2015). Synthesis of Functionalized Pyrazoles via Vanadium-Catalyzed C–N Dehydrogenative Cross-Coupling and Fluorescence Switch-On Sensing of BSA Protein. Org. Lett..

[30] Croce P.D., La Rosa C. (1984). A Convenient Synthesis of Indazoles. Synthesis.

[31] Counceller C.M., Eichman C.C., Wray B.C., Stambuli J.P. (2008). A Practical, Metal-Free Synthesis of 1H-Indazoles. Org. Lett..

[32] Shamsabadi A., Chudasama V. (2018). A facile route to 1H- and 2H-indazoles from readily accessible acyl hydrazides by exploiting a novel aryne-based molecular rearrangement. Chem. Commun..

[33] Malz F., Jancke H. (2005). Validation of quantitative NMR. J. Pharm. Biomed. Anal..

[34] Schoenberger T., Bernstein M., Mestrelab U.K., Braouet C., Ron Crouch J., Suematsu T., Guillou C., Klare H., Cologne B.W.Z., Bernie O. (2019). Guideline for QNMR Analysis. http://enfsi.eu/wp-content/uploads/2017/06/qNMR-Guideline_version001.pdf.

