# Supplementary material for: Design, Synthesis and Anticandidal Evaluation of Indazole and Pyrazole Derivatives
Source: Pharmaceuticals (Basel). 2021 Feb 24;14(3):176. doi: 10.3390/ph14030176 (PMC7996157; doi:10.3390/ph14030176)
Supplement: Supplementary file 1 [file pharmaceuticals-14-00176-s001.pdf]

Supplementary Materials

Design, synthesis and anticandidal evaluation of indazole and pyrazole derivatives

Karen Rodríguez-Villar [1], Alicia Hernández-Campos [2], Lilián Yépez-Mulia [3], Teresita del Rosario Sainz-Espuñes [4], Olivia Soria-Arteche [4], Juan Francisco Palacios-Espinosa [4], Francisco Cortés-Benítez [4], Martha Leyte-Lugo [5], Bárbara Varela-Petrissans [4], Edgar A. Quintana-Salazar [4] and Jaime Pérez-Villanueva [4,*]

[1] Doctorado en Ciencias Biológicas y de la Salud, Universidad Autónoma Metropolitana (UAM), Ciudad de México 04960, Mexico; qkarenrodv@hotmail.com (K.R.-V.).

[2] Departamento de Farmacia, Facultad de Química, Universidad Nacional Autónoma de México (UNAM), 04510 Ciudad de México, Mexico; hercam@unam.mx (A.H.-C.).

[3] Unidad de Investigación Médica en Enfermedades Infecciosas y Parasitarias, UMAE Hospital de Pediatría, Centro Médico Siglo XXI, Instituto Mexicano del Seguro Social, Ciudad de México 06720, Mexico; lilianyepez@yahoo.com (L.Y.-M.).

[4] Departamento de Sistemas Biológicos, División de Ciencias Biológicas y de la Salud, Universidad Autónoma Metropolitana-Xochimilco (UAM-X), Ciudad de México 04960, Mexico; trsainz@correo.xoc.uam.mx (T.R.S.-E.), soriao@correo.xoc.uam.mx (O.S.-A.), jpalacios@correo.xoc.uam.mx (J.F.P.-E.), jcortesb@correo.xoc.uam.mx (F.C.-B.), bvra5302@gmail.com (B.V.-P.), edgarqsl2811@gmail.com (E.A.Q.-S.), jpvillanueva@correo.xoc.uam.mx (J.P.-V.).

[5] Catedrático CONACyT comisionado al Departamento de Sistemas Biológicos, División de Ciencias Biológicas y de la Salud, Universidad Autónoma Metropolitana-Xochimilco (UAM-X), Ciudad de México 04960, Mexico; mleyte@correo.xoc.uam.mx (M.L.-L.).

* Correspondence: jpvillanueva@correo.xoc.uam.mx; Tel.: +525 54 83 72 59; fax: +525 55 9479 29.

Cylinder plate method

and

^{1}H NMR, ^{13}C NMR and MS spectra

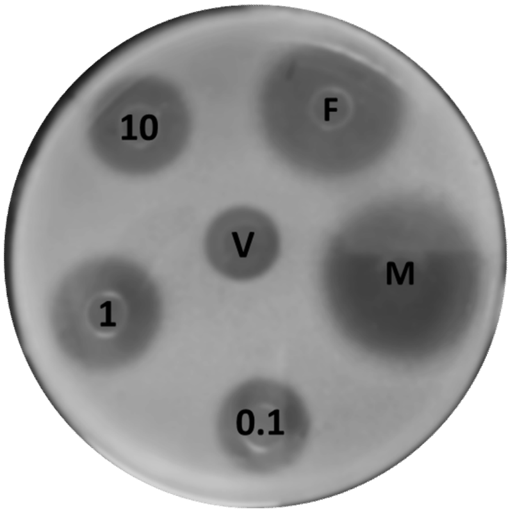

Figure S1. Determination of the inhibition zone for compound **2b** tested at 10, 1 at 0.1mM against *C. albicans*. DMSO was included as a solvent control (V), fluconazole (F) and miconazole (M) were used as positive controls.

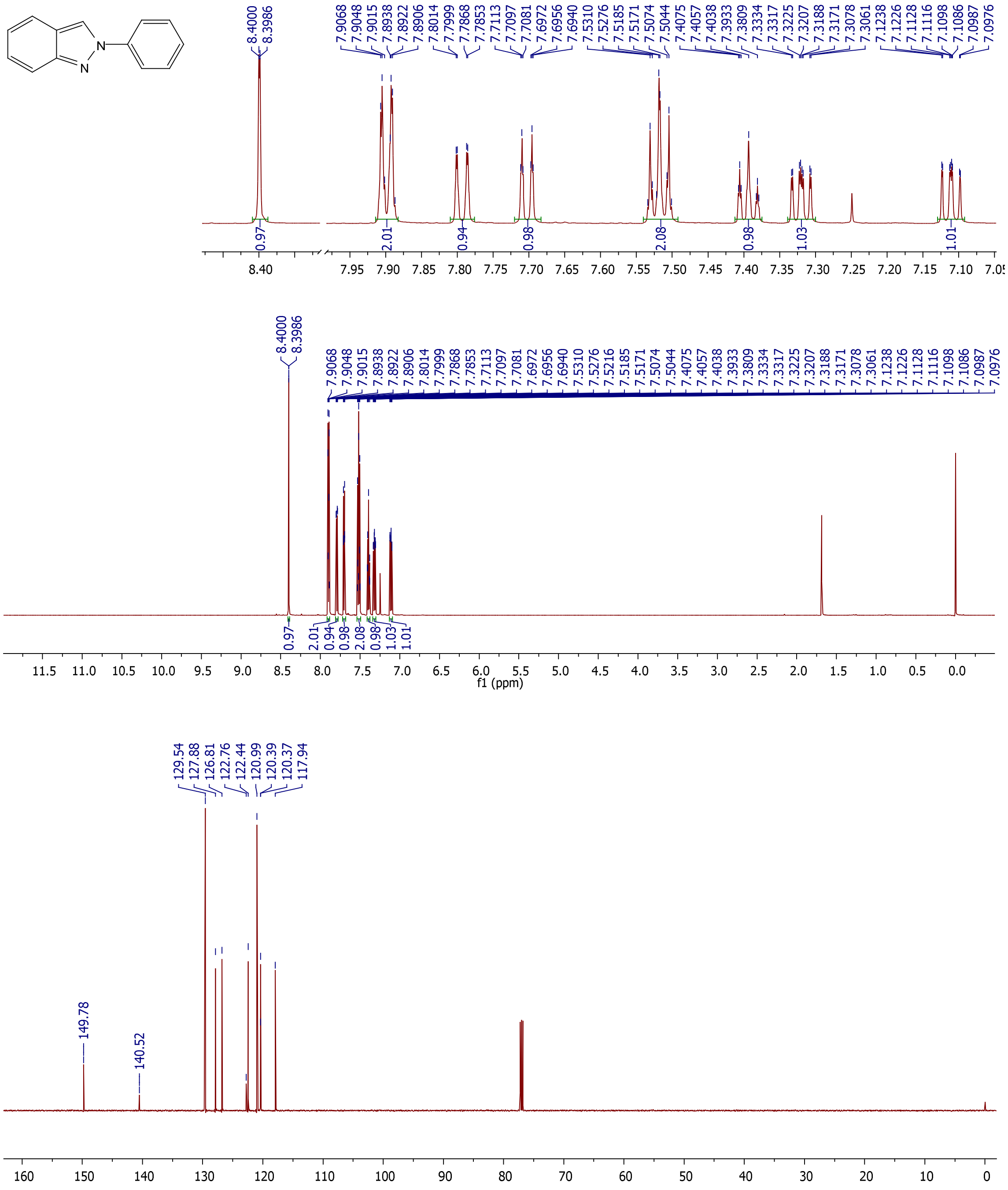

Figure S2. ¹H and ¹³C NMR of *2-phenyl-2H-indazole* **2a.**

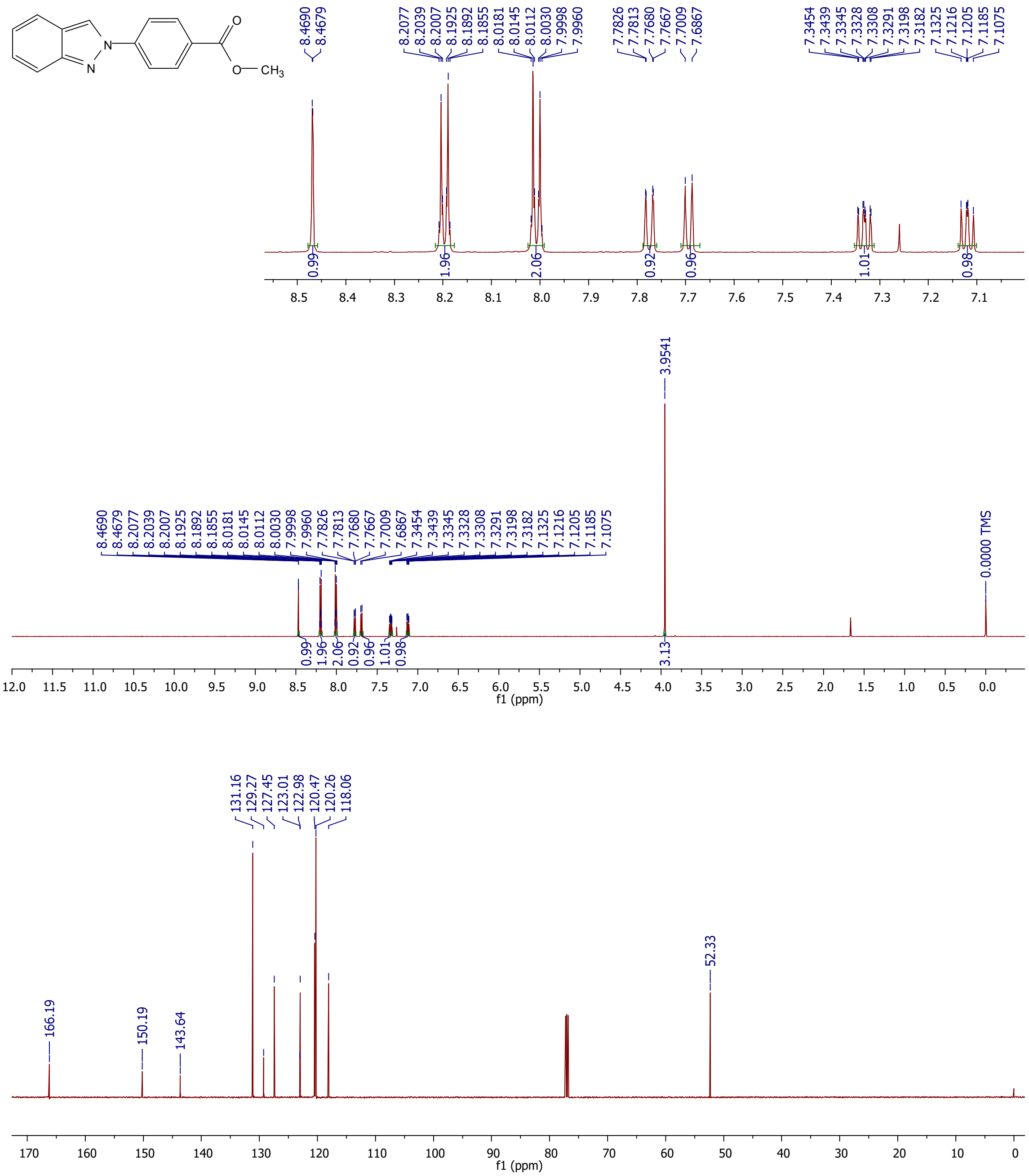

Figure S3. ^{1}H and ^{13}C NMR of *methyl 4-(2H-indazol-2-yl) benzoate* **2b**.

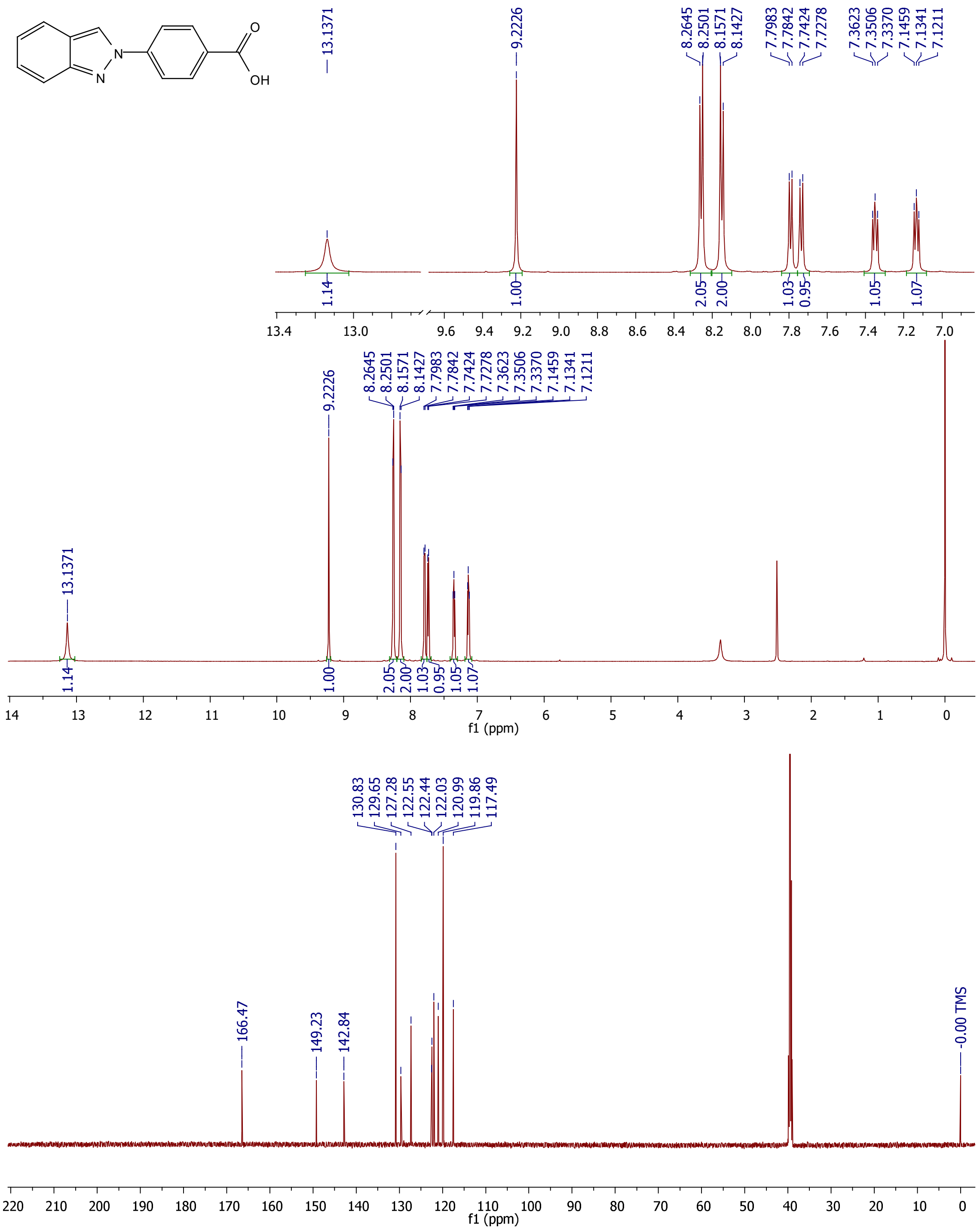

Figure S4. ^{1}H and ^{13}C NMR of *4-(2H-indazol-2-yl) benzoic acid* **2c**

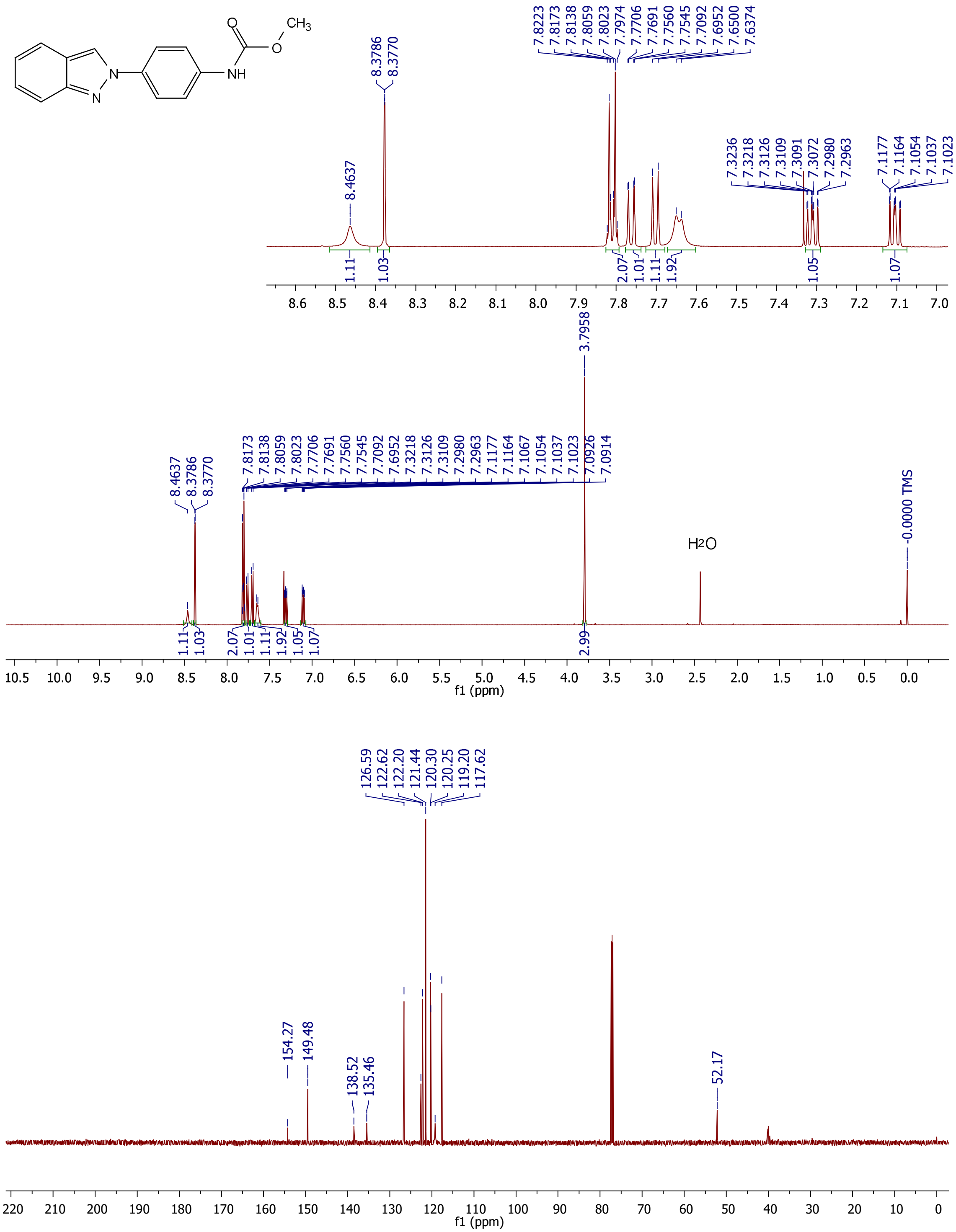

Figure S5. ^{1}H and ^{13}C NMR of. *Methyl (4-(2H-indazol-2-yl)phenyl)carbamate* **2d**

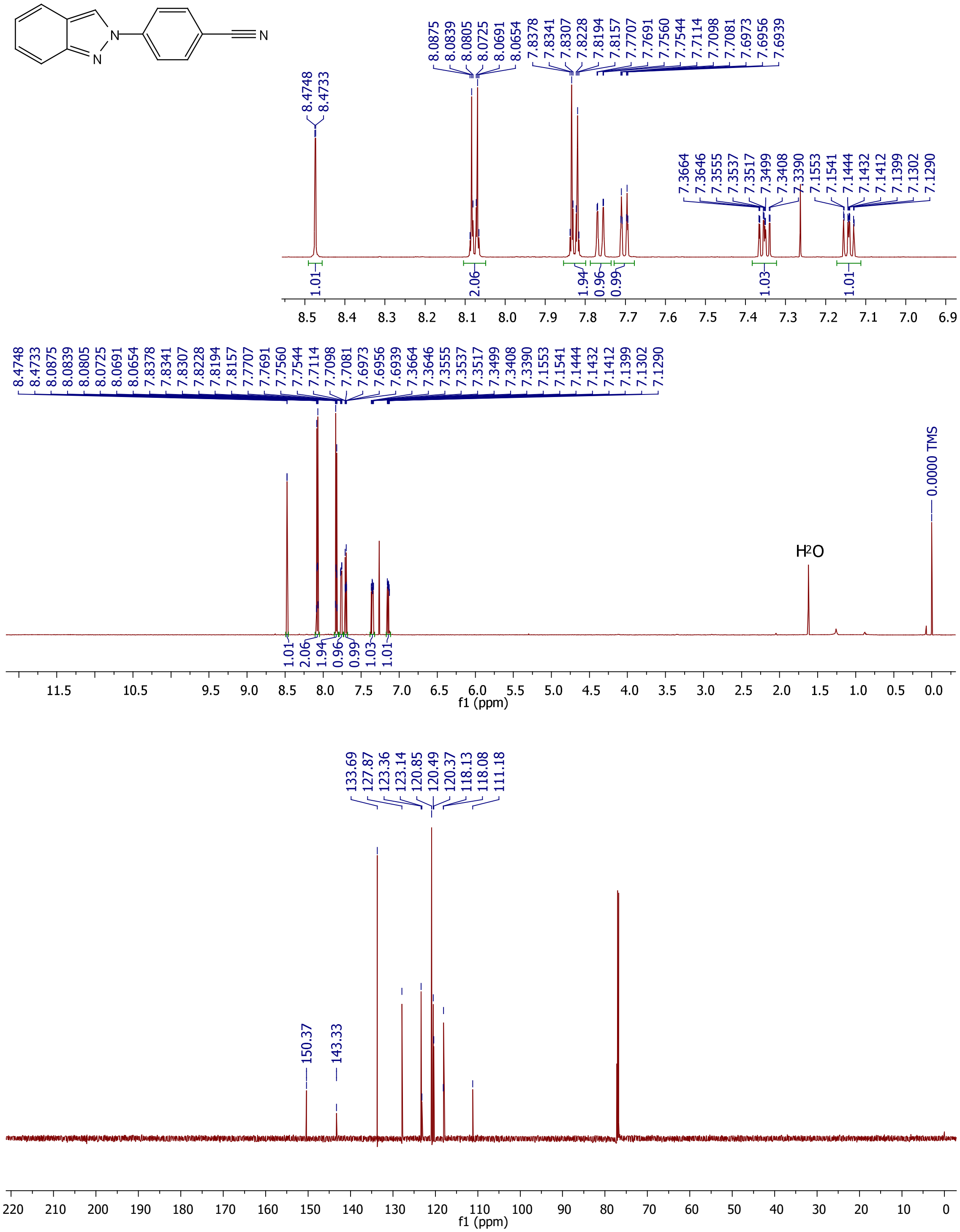

Figure S6. ^{1}H and ^{13}C NMR of *4-(2H-indazol-2-yl)benzonitrile* **2e**.

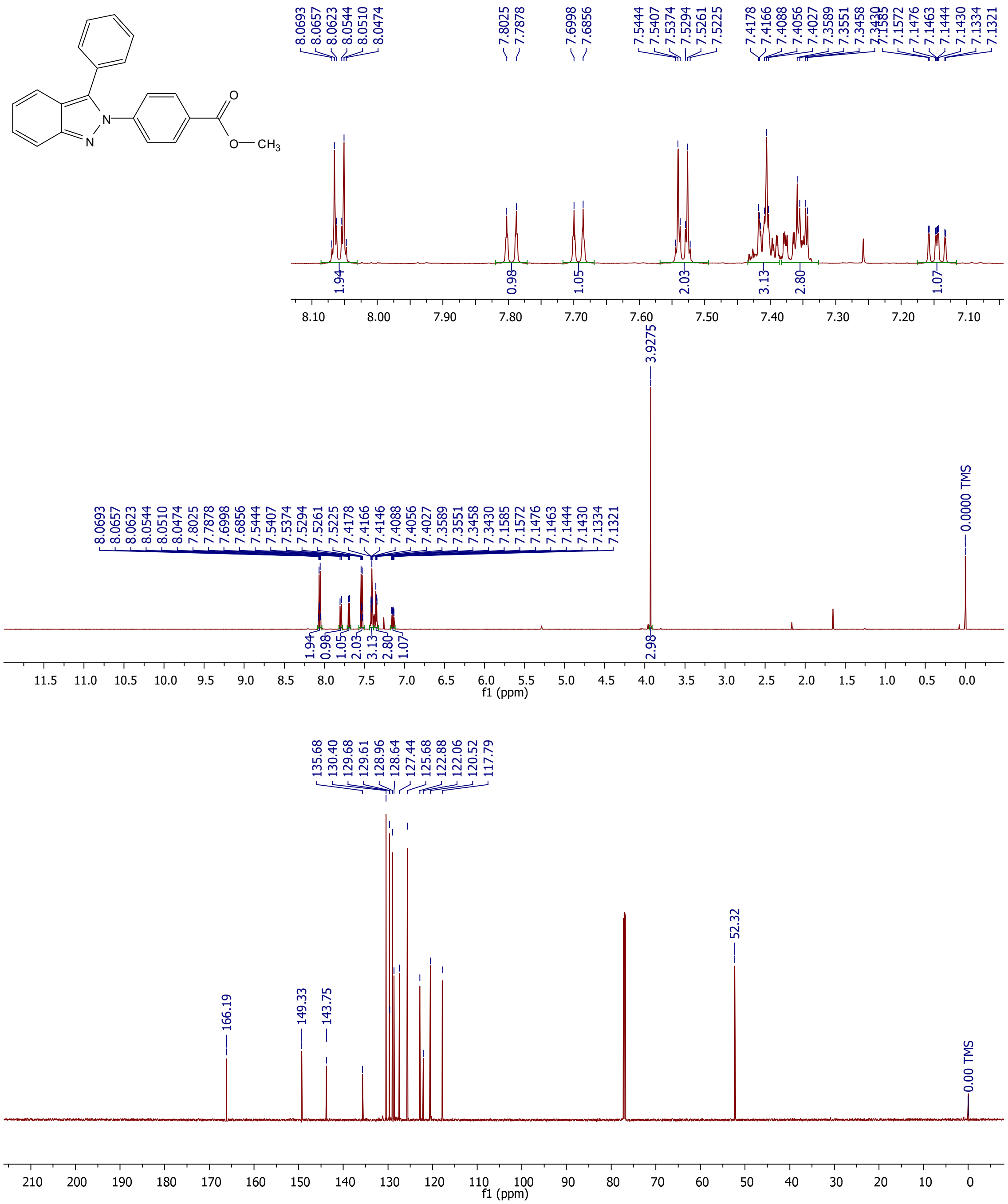

Figure S7. ^{1}H and ^{13}C NMR of *methyl 4-(3-phenyl-2H-indazol-2-yl) benzoate* **3a**.

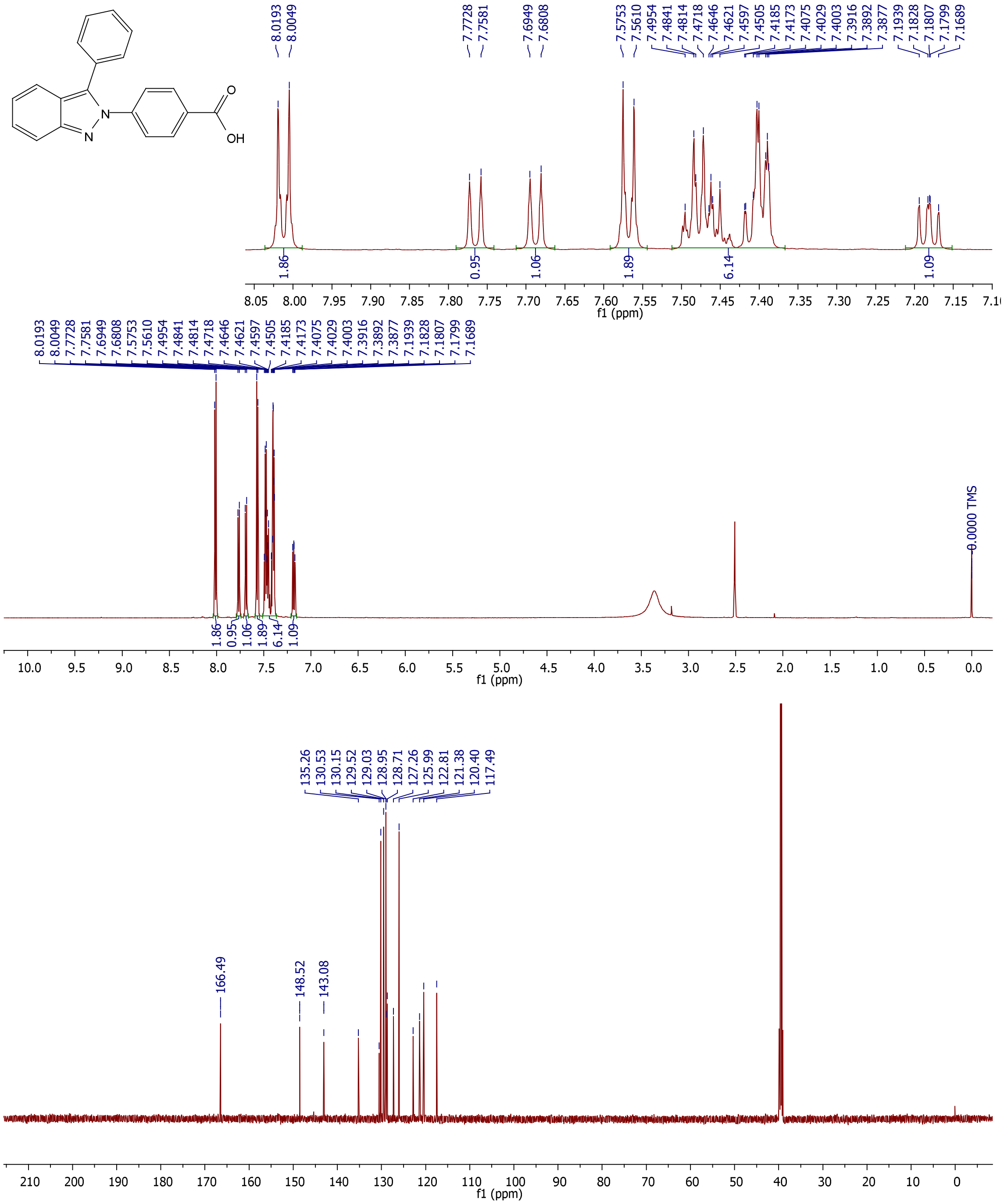

Figure S8. ^{1}H and ^{13}C NMR of *4-(3-phenyl-2H-indazol-2-yl) benzoic acid* **3b**

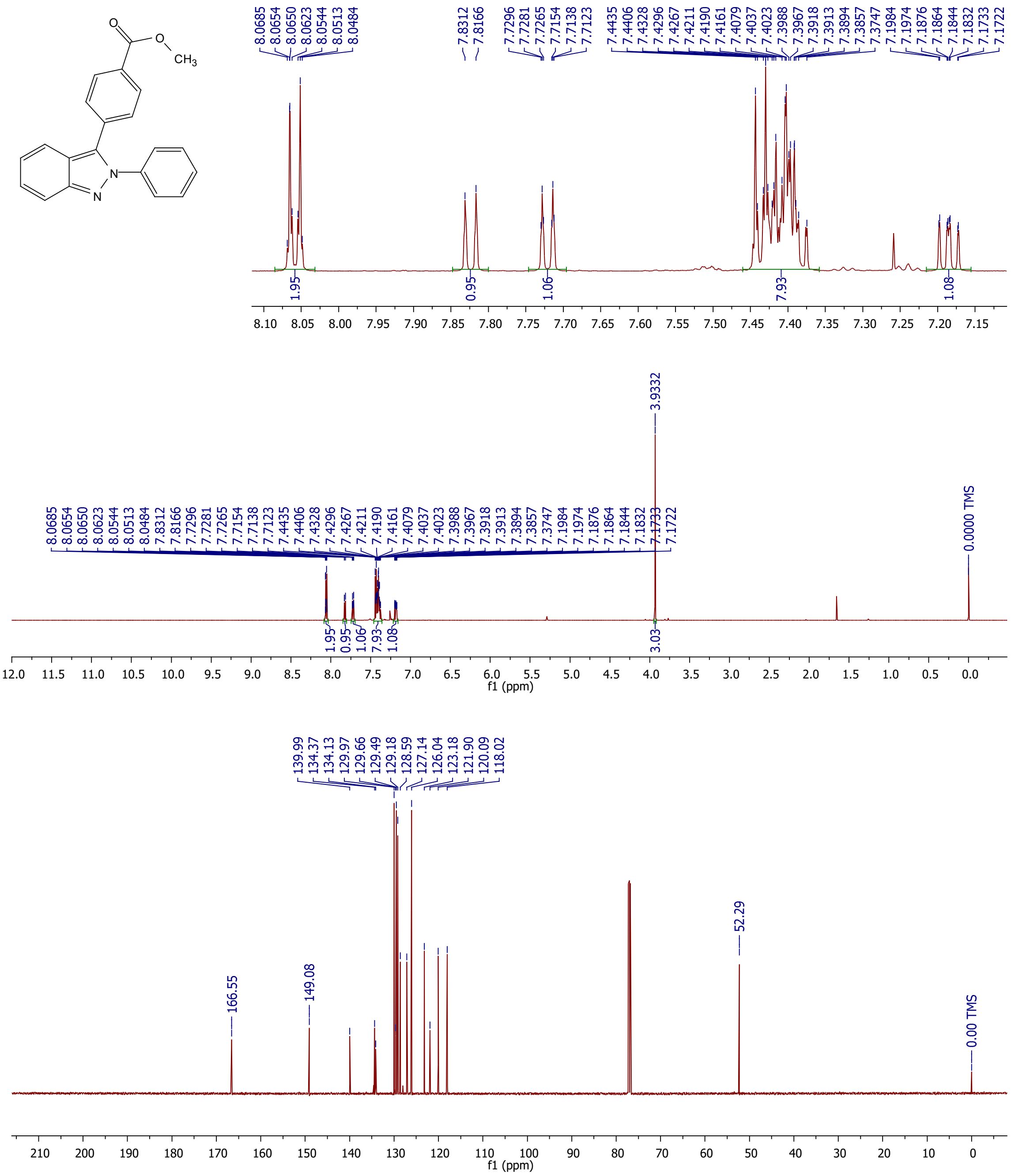

Figure S9. ^{1}H and ^{13}C NMR of *methyl 4-(2-phenyl-2H-indazol-3-yl) benzoate* **3c**

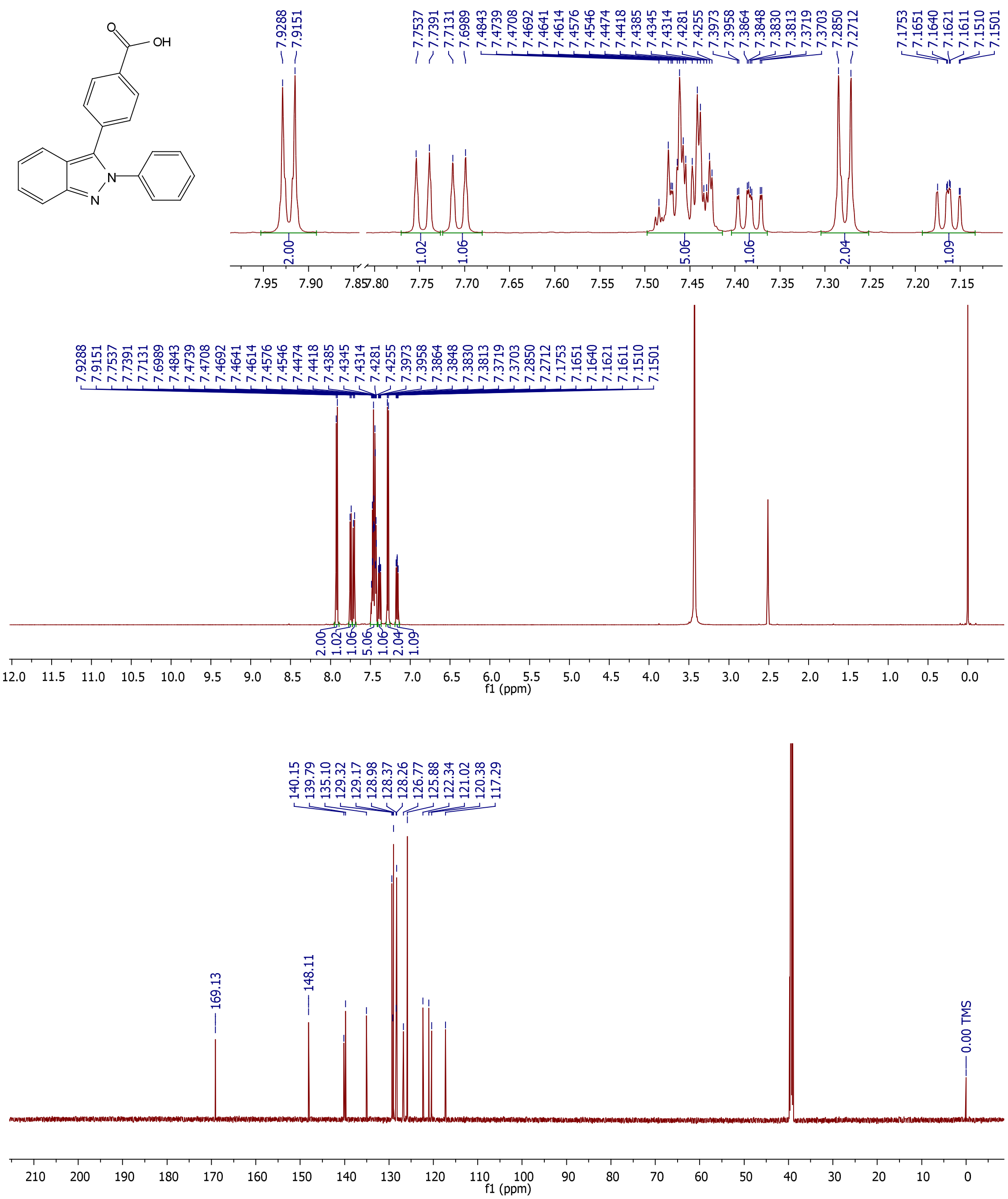

Figure S10. ^{1}H and ^{13}C NMR of *4-(2-phenyl-2H-indazol-3-yl) benzoic acid* **3d**

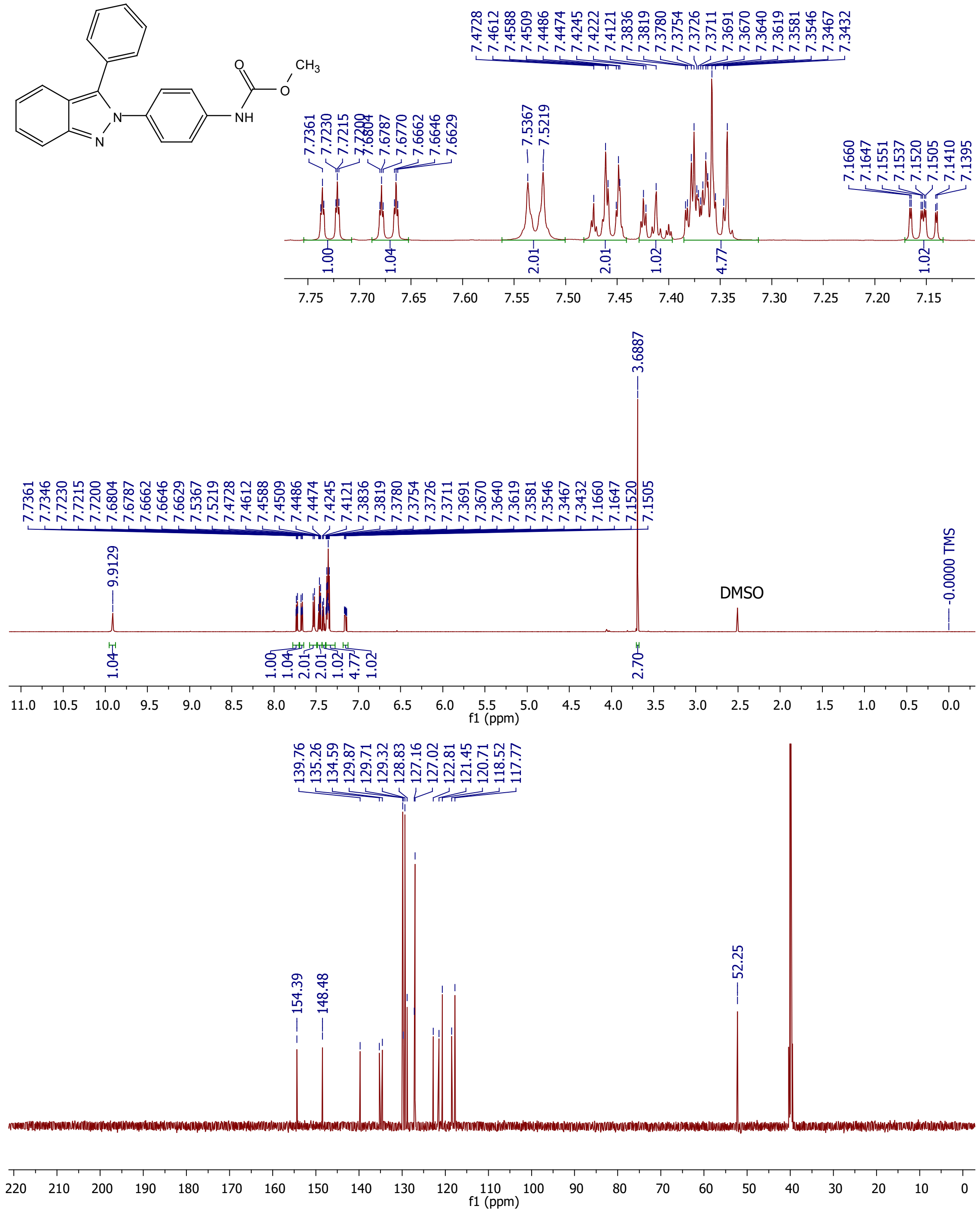

Figure S11. ^{1}H and ^{13}C NMR of *Methyl (4-(3-phenyl-2H-indazol-2-yl)phenyl)carbamate* **3e**.

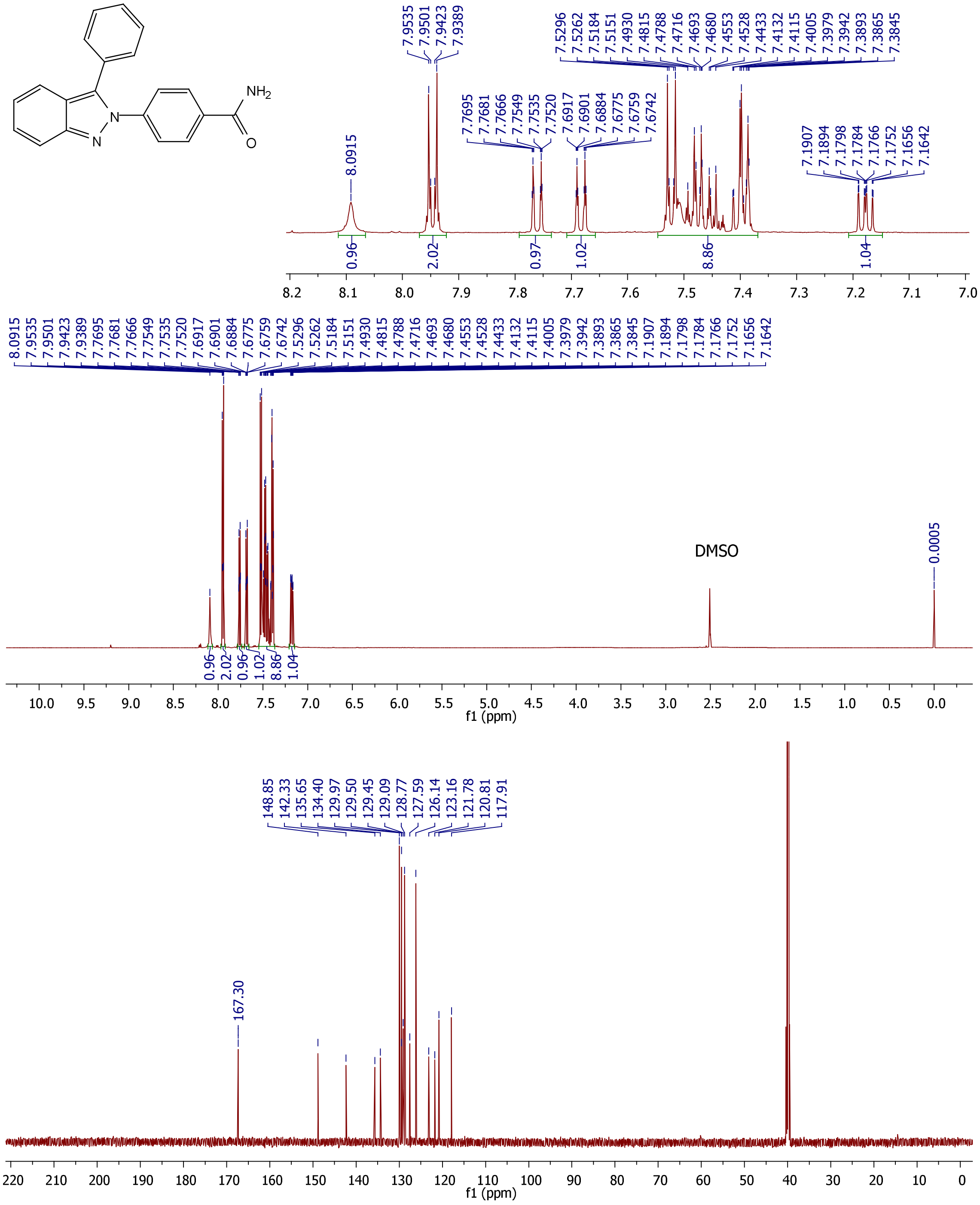

Figure S12. ^{1}H and ^{13}C NMR of *4-(3-phenyl-2H-indazol-2-yl)benzamide* **3f**.

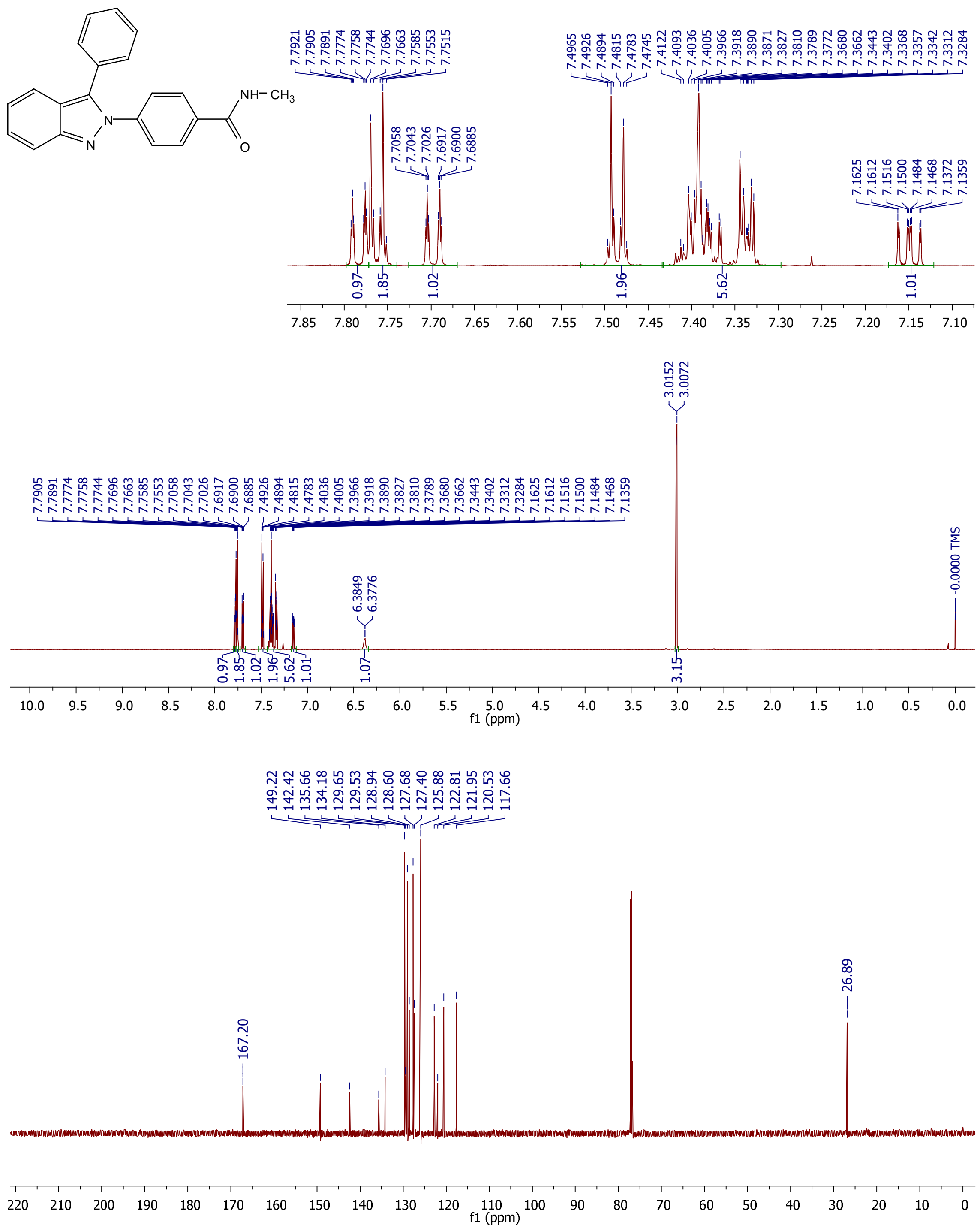

Figure S13. ^{1}H and ^{13}C NMR of *N-methyl-4-(3-phenyl-2H-indazol-2-yl)benzamide* **3g**.

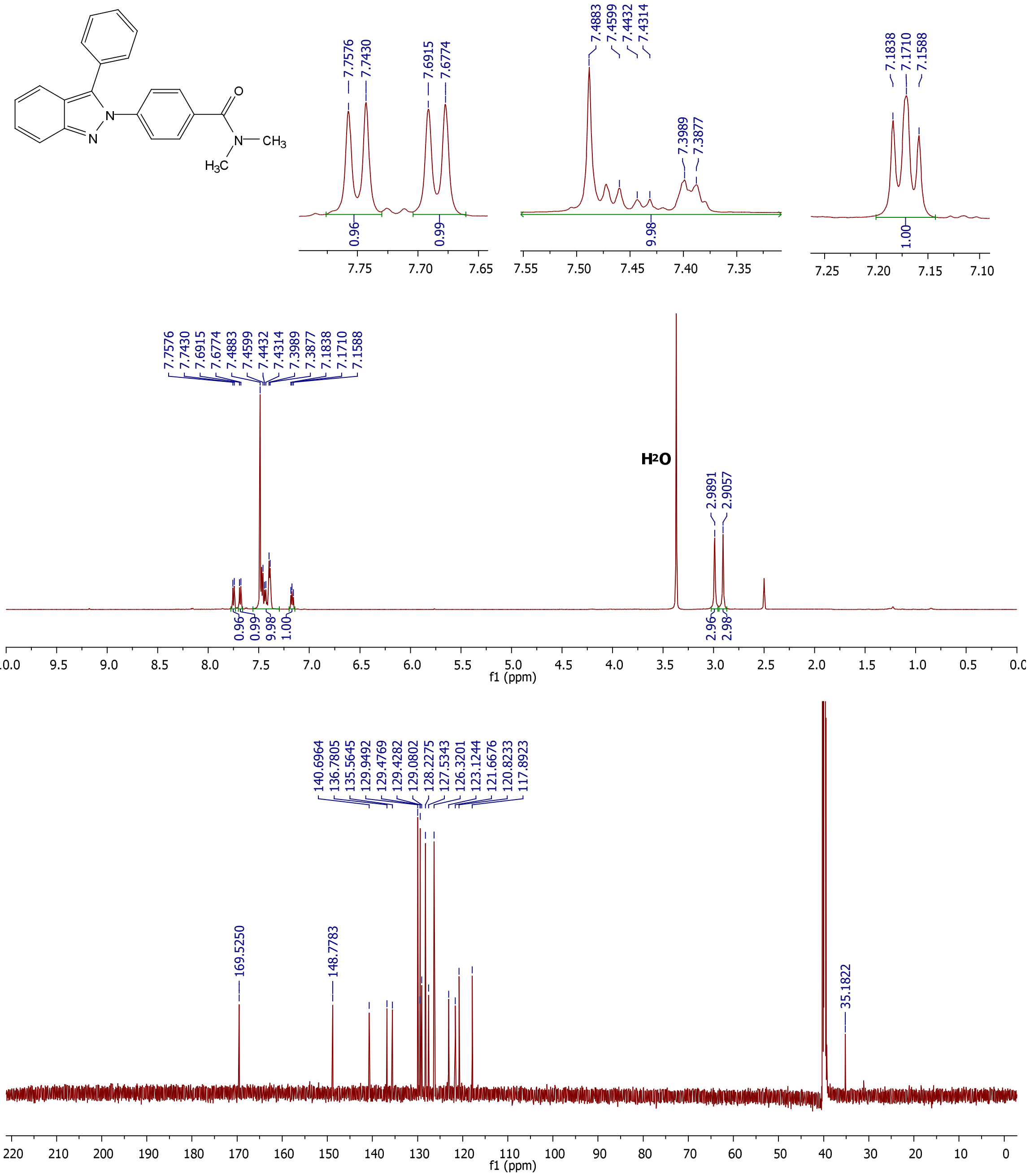

Figure S14. ^{1}H and ^{13}C NMR of *N,N-Dimethyl-4-(3-phenyl-2H-indazol-2-yl)benzamide* **3h**.

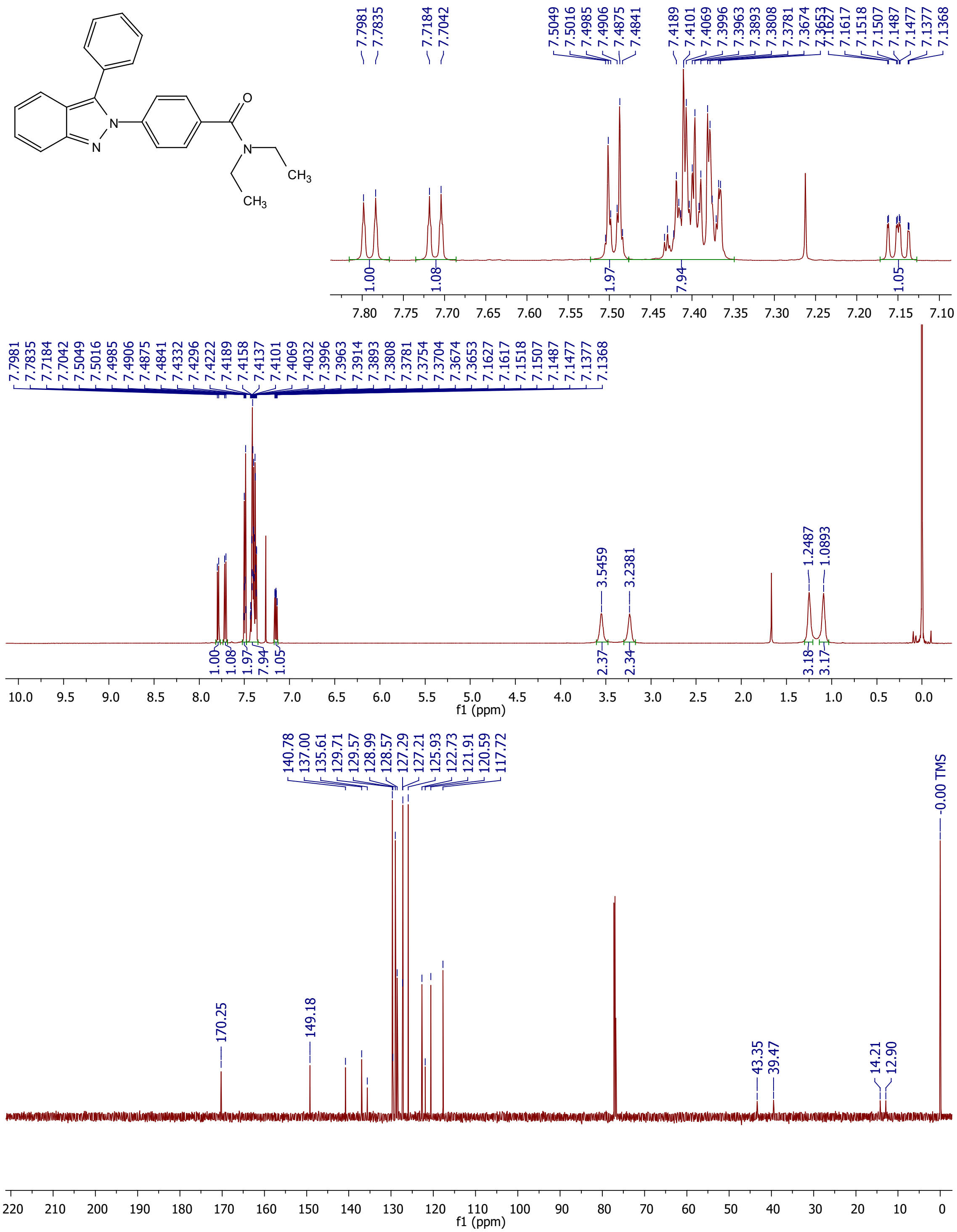

Figure S15. ^{1}H and ^{13}C NMR of *N,N-diethyl-4-(3-phenyl-2H-indazol-2-yl)benzamide* **3i**.

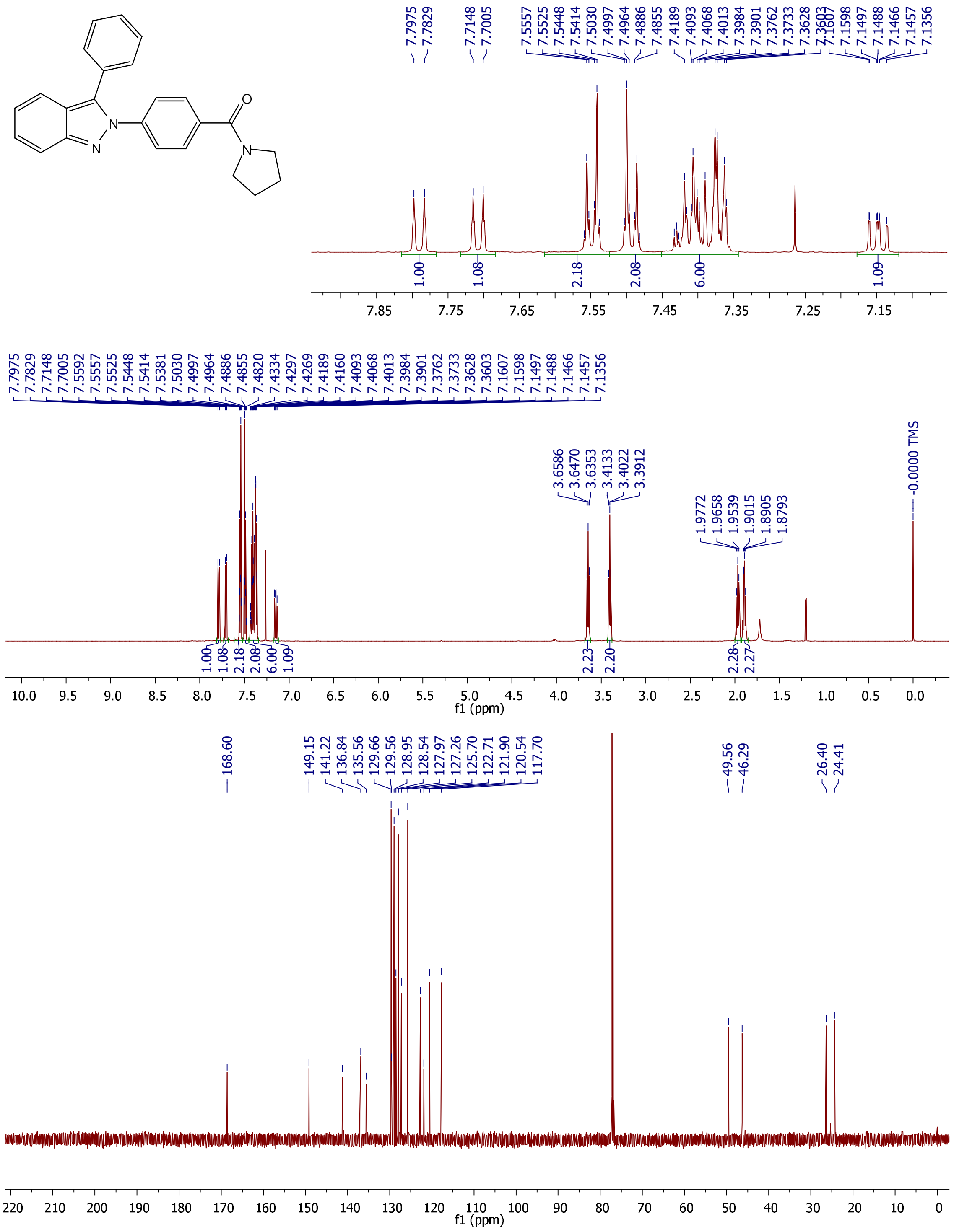

Figure S16. ^{1}H and ^{13}C NMR of *(4-(3-phenyl-2H-indazol-2-yl)phenyl)(pyrrolidin-1-yl)methanone* **3j**.

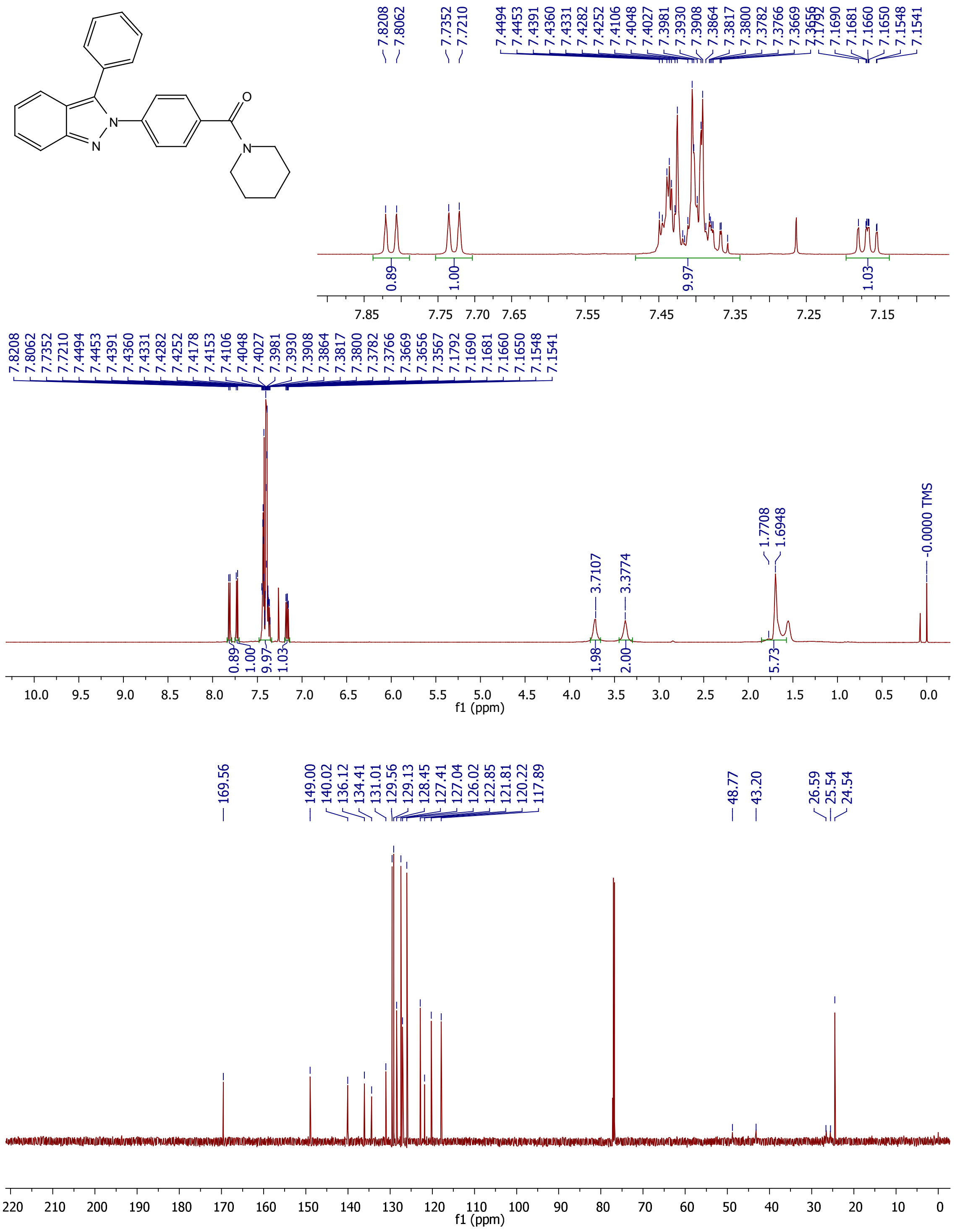

Figure S17. ^{1}H and ^{13}C NMR of *(4-(3-phenyl-2H-indazol-2-yl)phenyl)(piperidin-1-yl)methanone* **3k**.

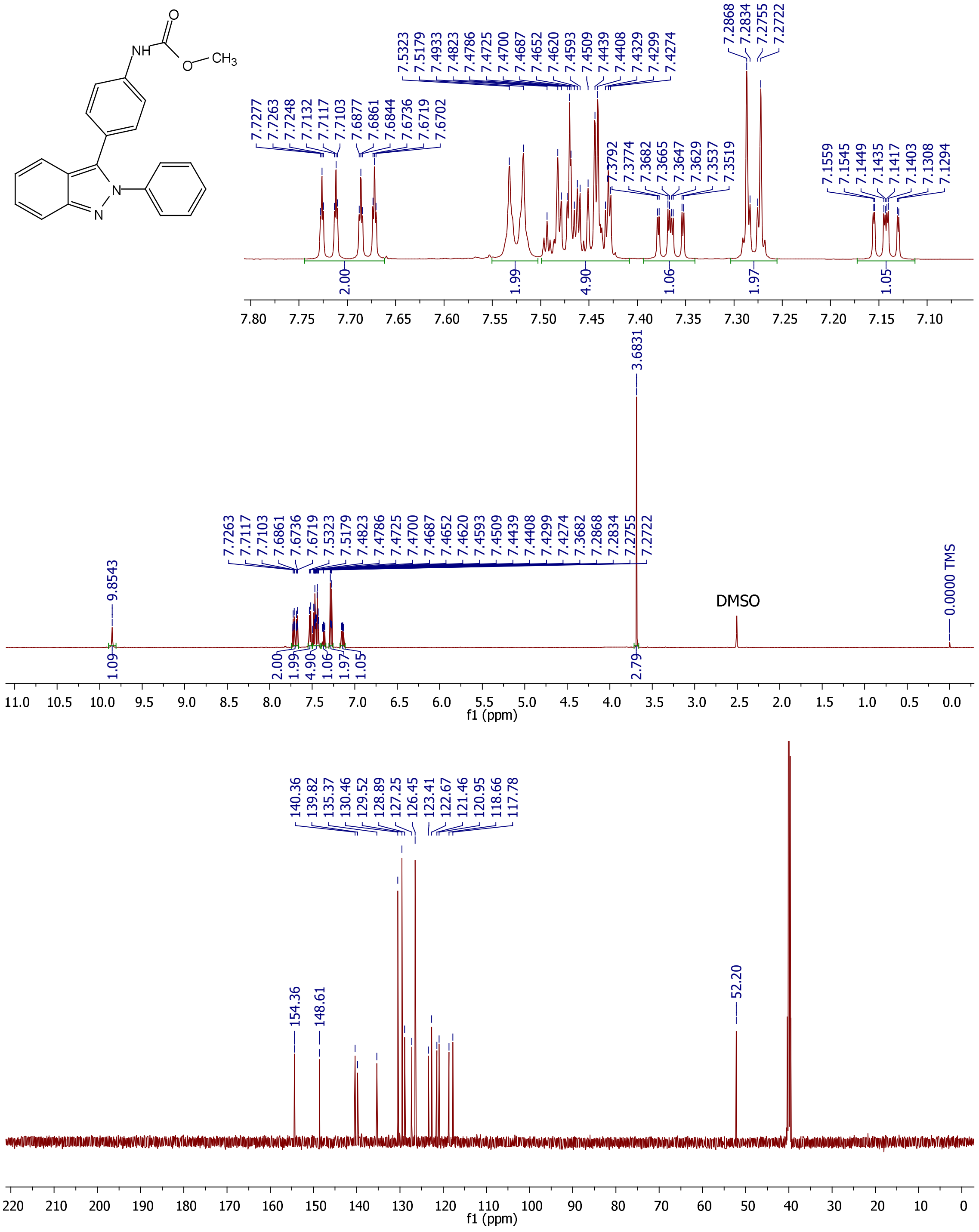

Figure S18. ^{1}H and ^{13}C NMR of *methyl (4-(2-phenyl-2H-indazol-3-yl)phenyl)carbamate* **3l**.

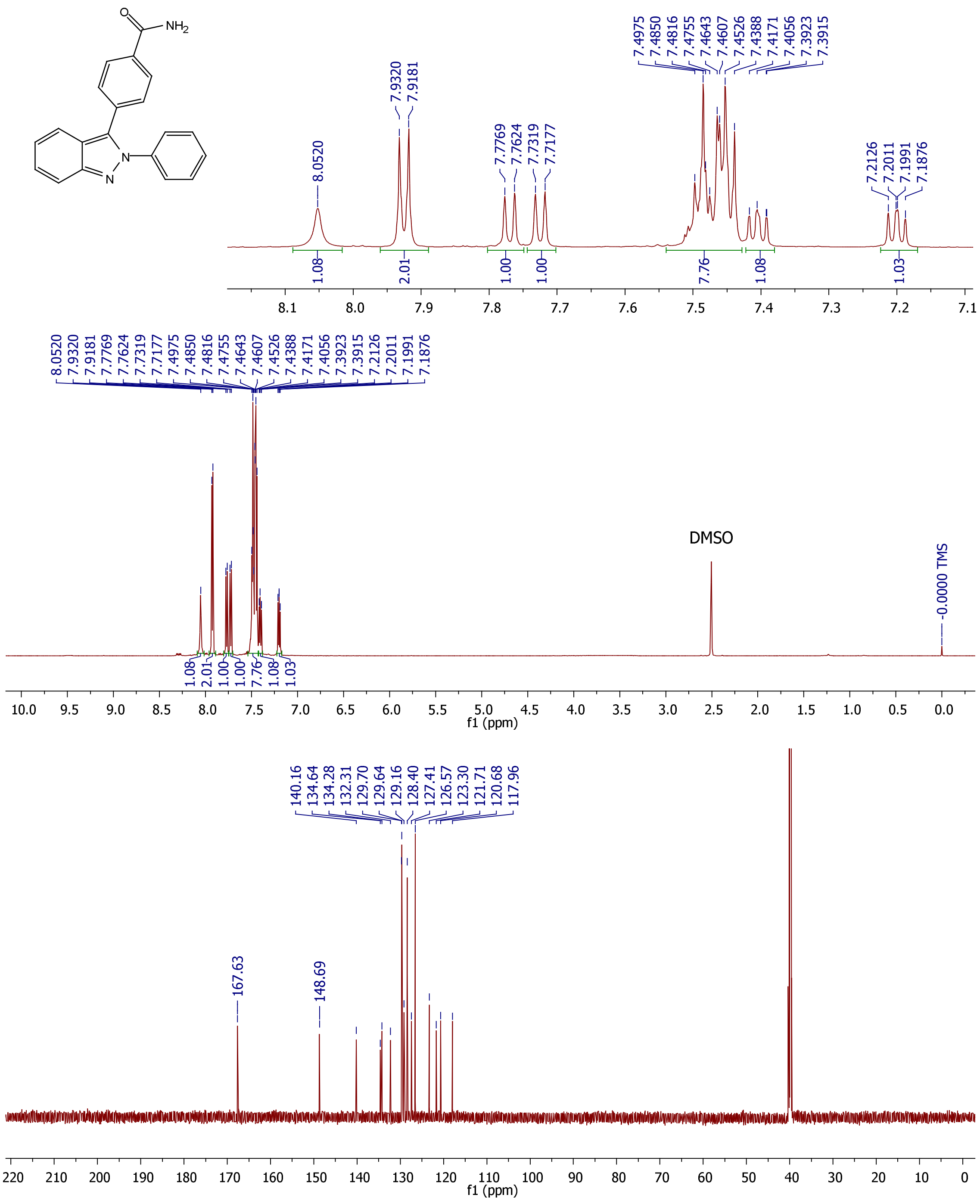

Figure S19. ^{1}H and ^{13}C NMR of *4-(2-phenyl-2H-indazol-3-yl)benzamide* **3m**.

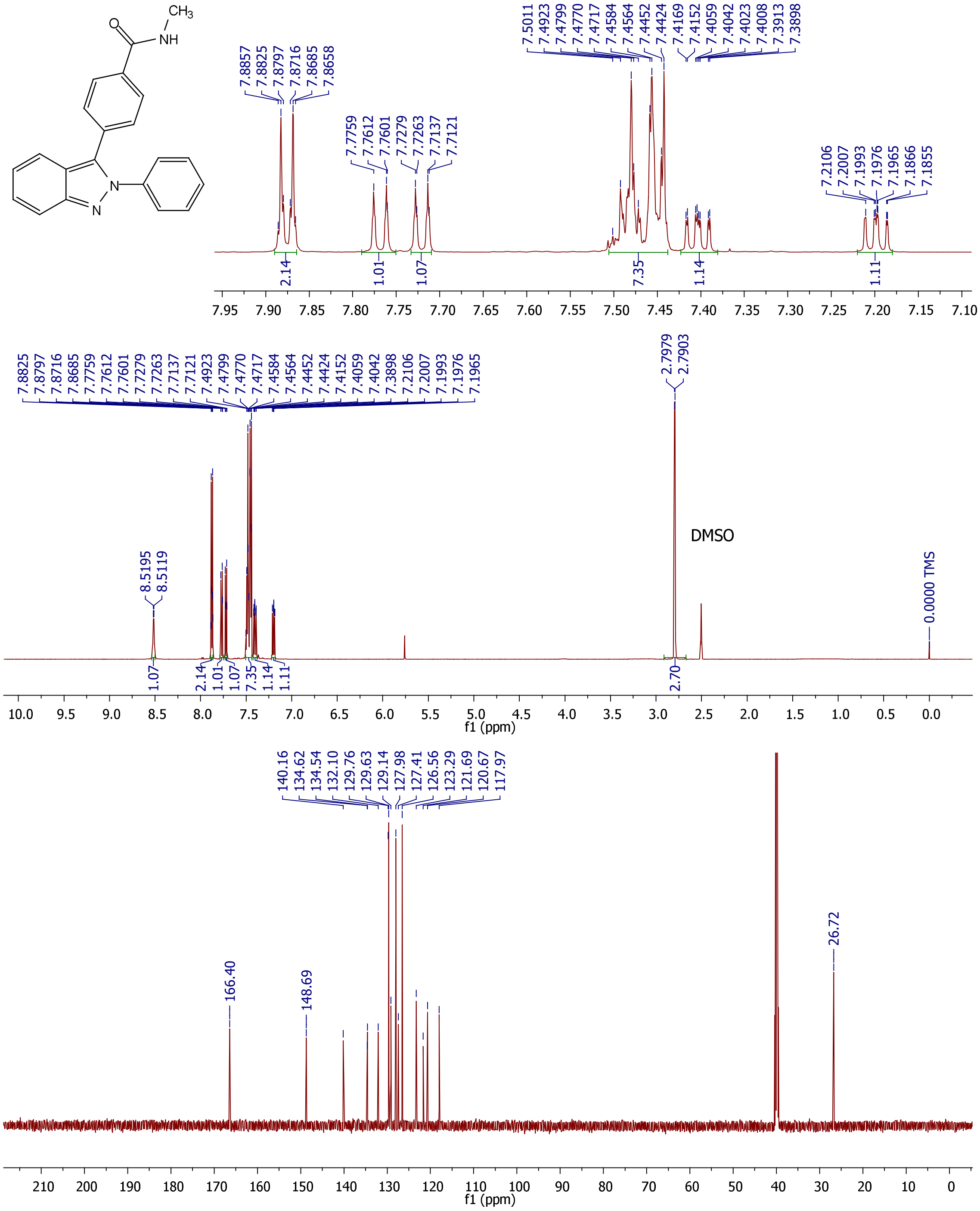

Figure S20. ^{1}H and ^{13}C NMR of *N-methyl-4-(2-phenyl-2H-indazol-3-yl)benzamide* **3n**

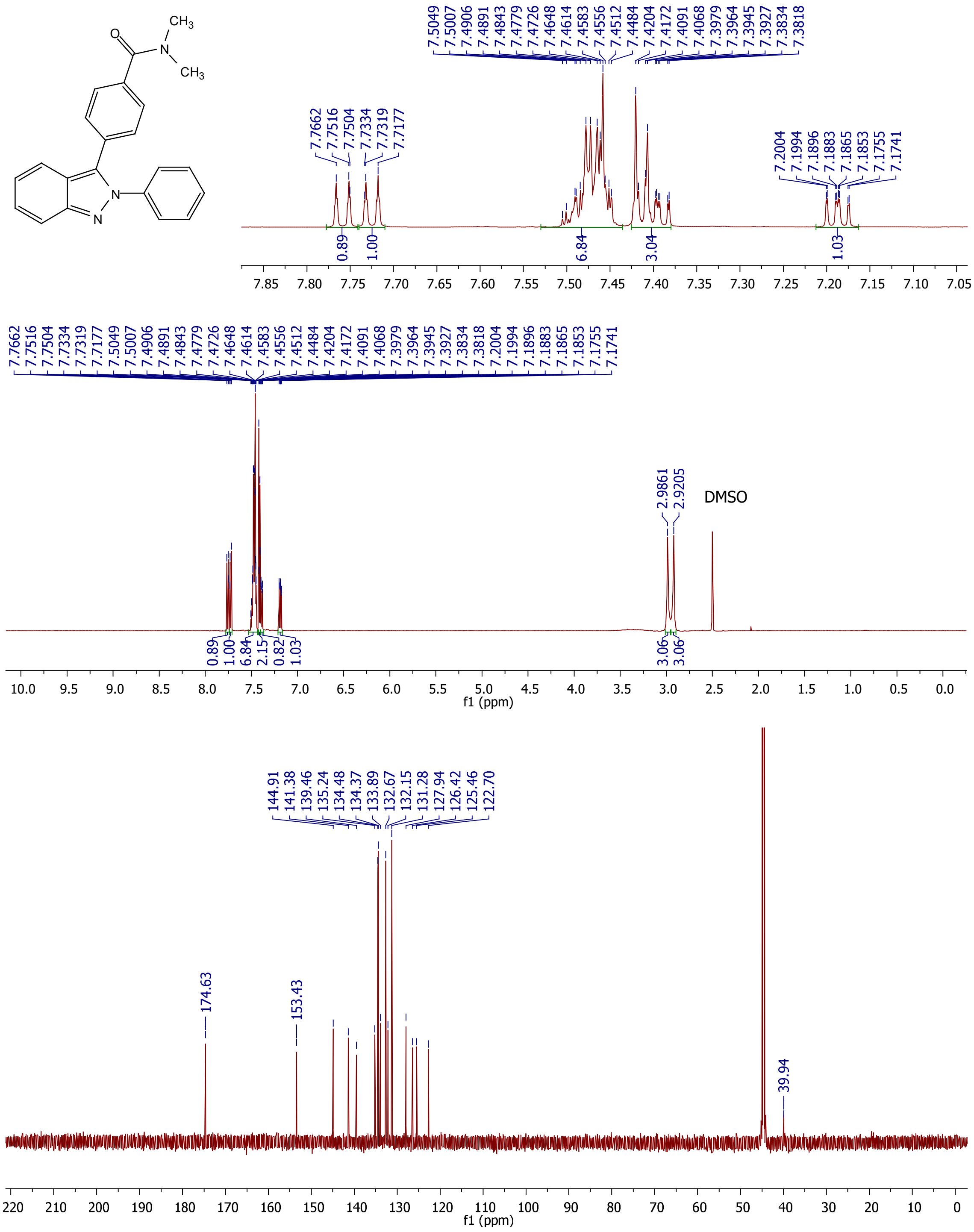

Figure S21. ¹H and ¹³C NMR of *N,N-dimethyl-4-(3-phenyl-2H-indazol-2-yl)benzamide* **3o**

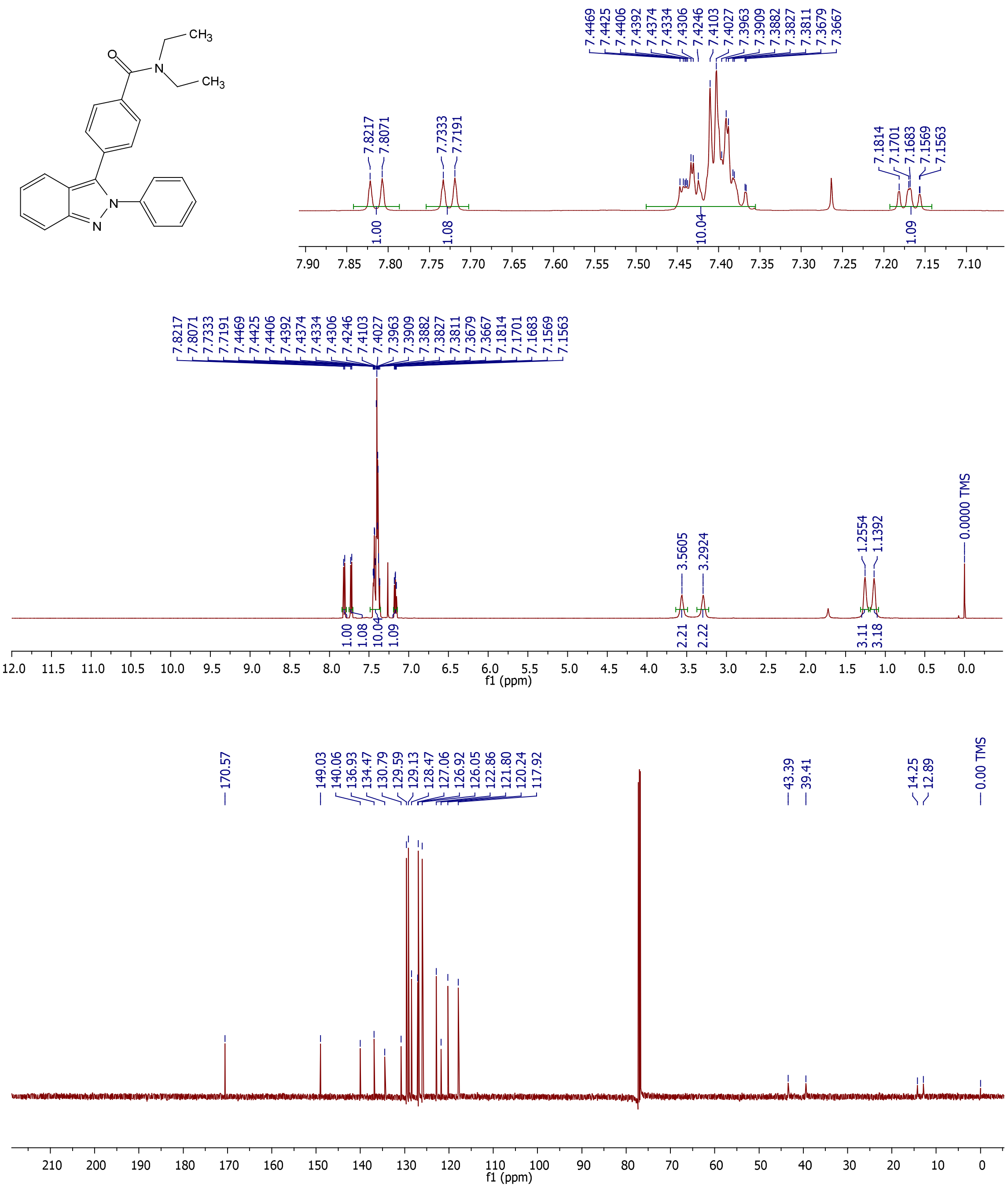

Figure S22. ^{1}H and ^{13}C NMR of *N,N-diethyl-4-(2-phenyl-2H-indazol-3-yl)benzamide* **3p.**

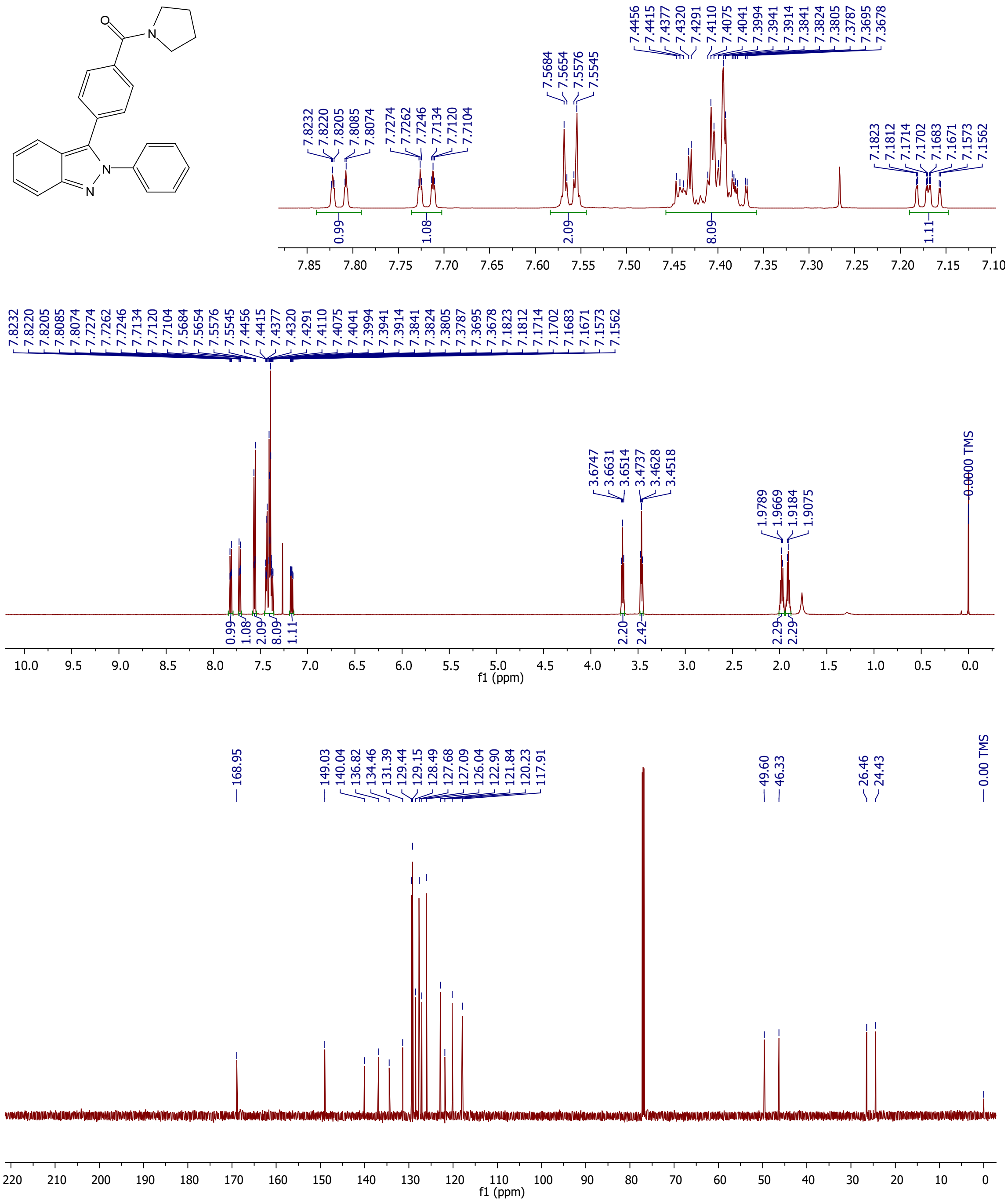

Figure S23. ^{1}H and ^{13}C NMR of *(4-(2-phenyl-2H-indazol-3-yl)phenyl)(pyrrolidin-1-yl)methanone* **3q**.

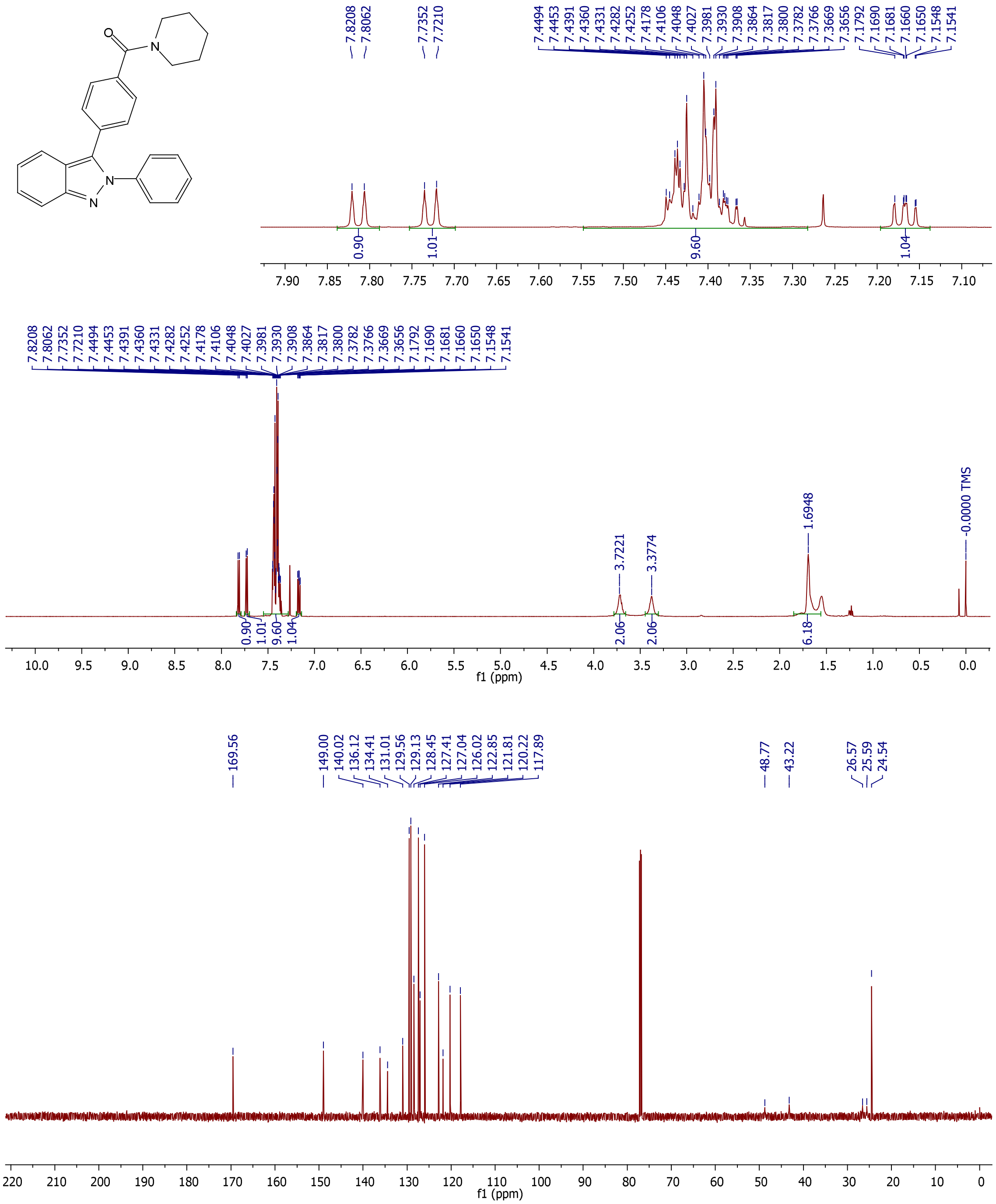

Figure S24. ^{1}H and ^{13}C NMR of *(4-(2-phenyl-2H-indazol-3-yl)phenyl)(piperidin-1-yl)methanone* **3r**

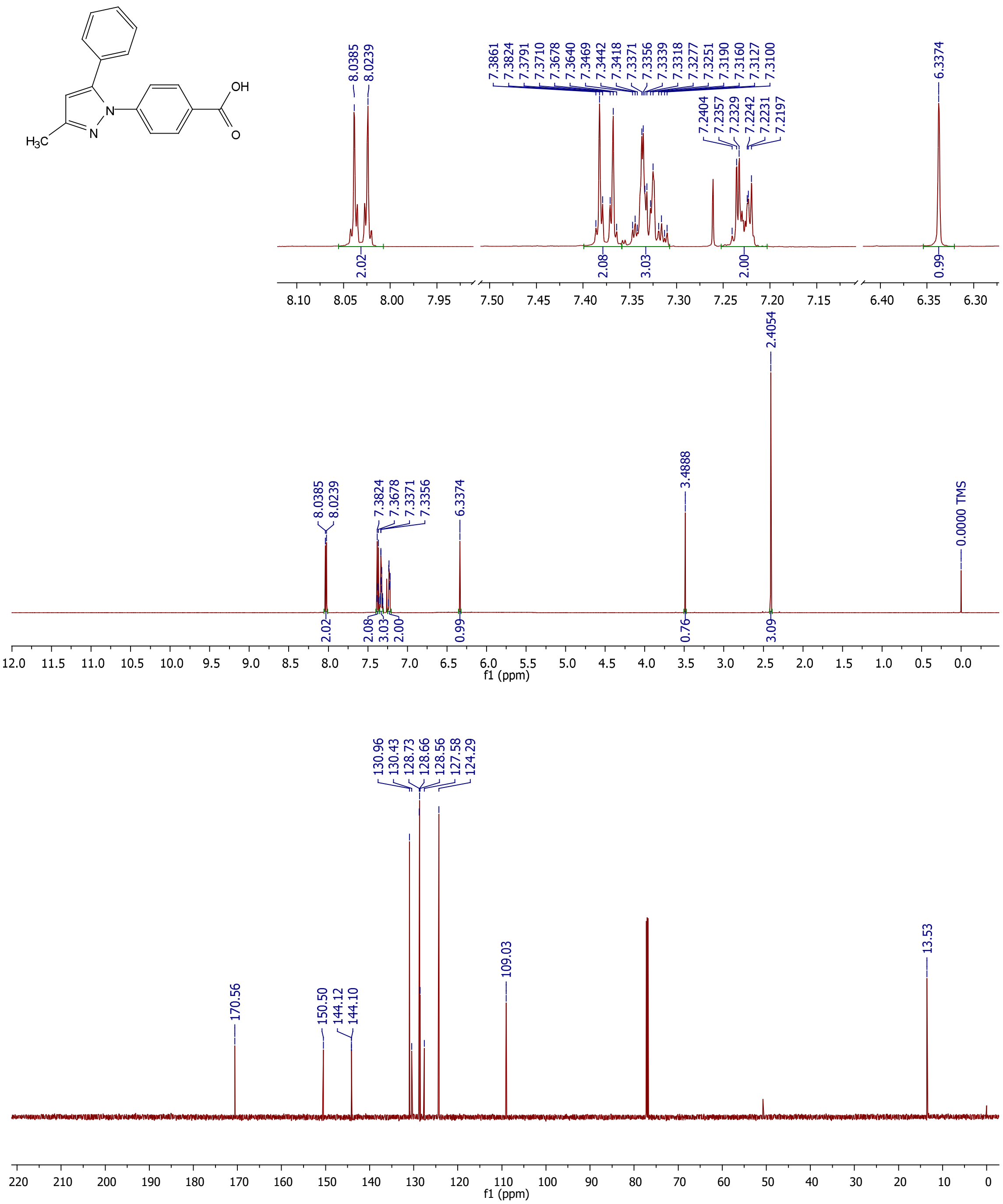

Figure S25. ^{1}H and ^{13}C NMR of *4-(3-methyl-5-phenyl-1H-pyrazol-1-yl)benzoic acid* **6a**

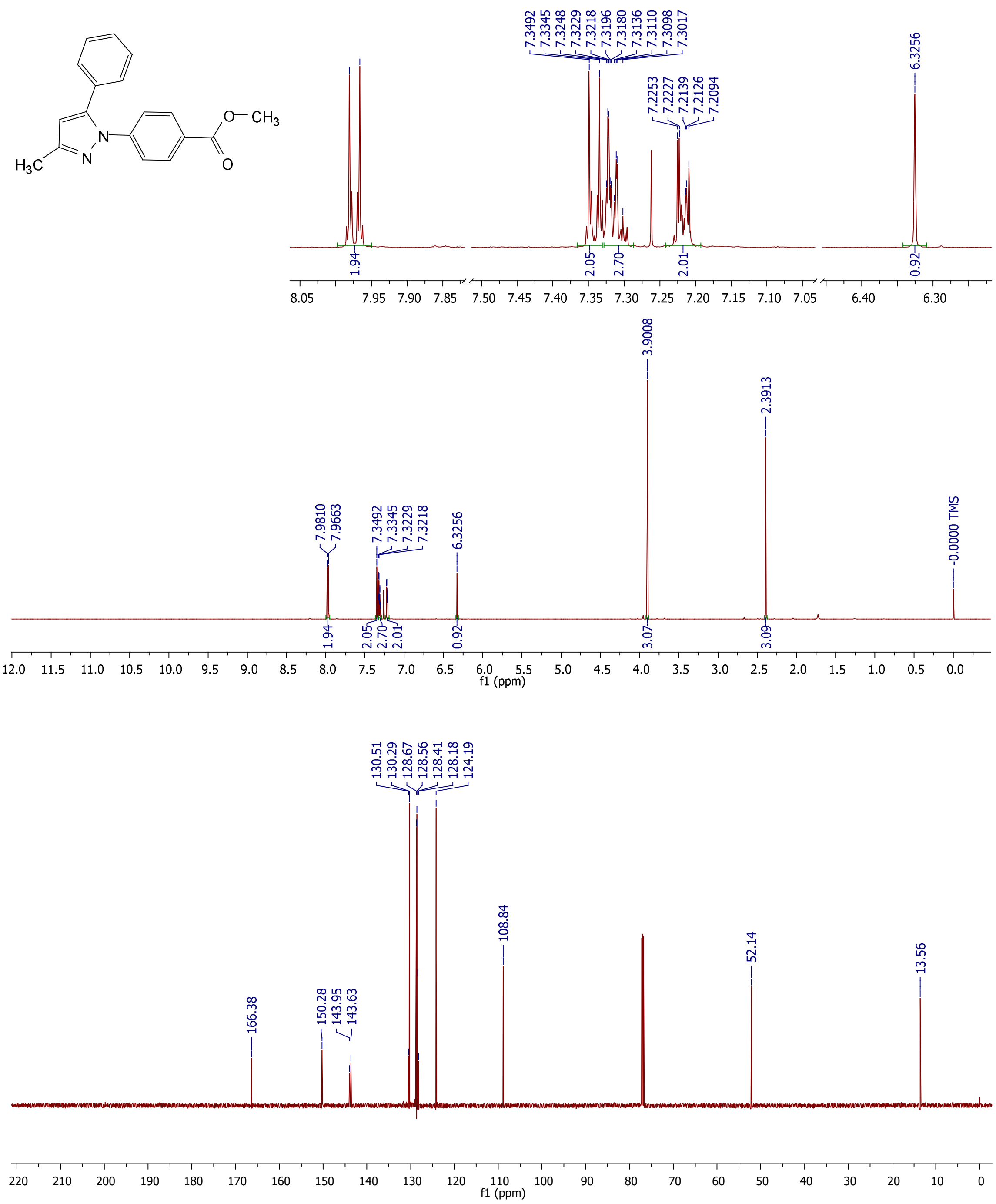

Figure S26. ^{1}H and ^{13}C NMR of *methyl 4-(3-methyl-5-phenyl-1H-pyrazol-1-yl)benzoate* **6b**

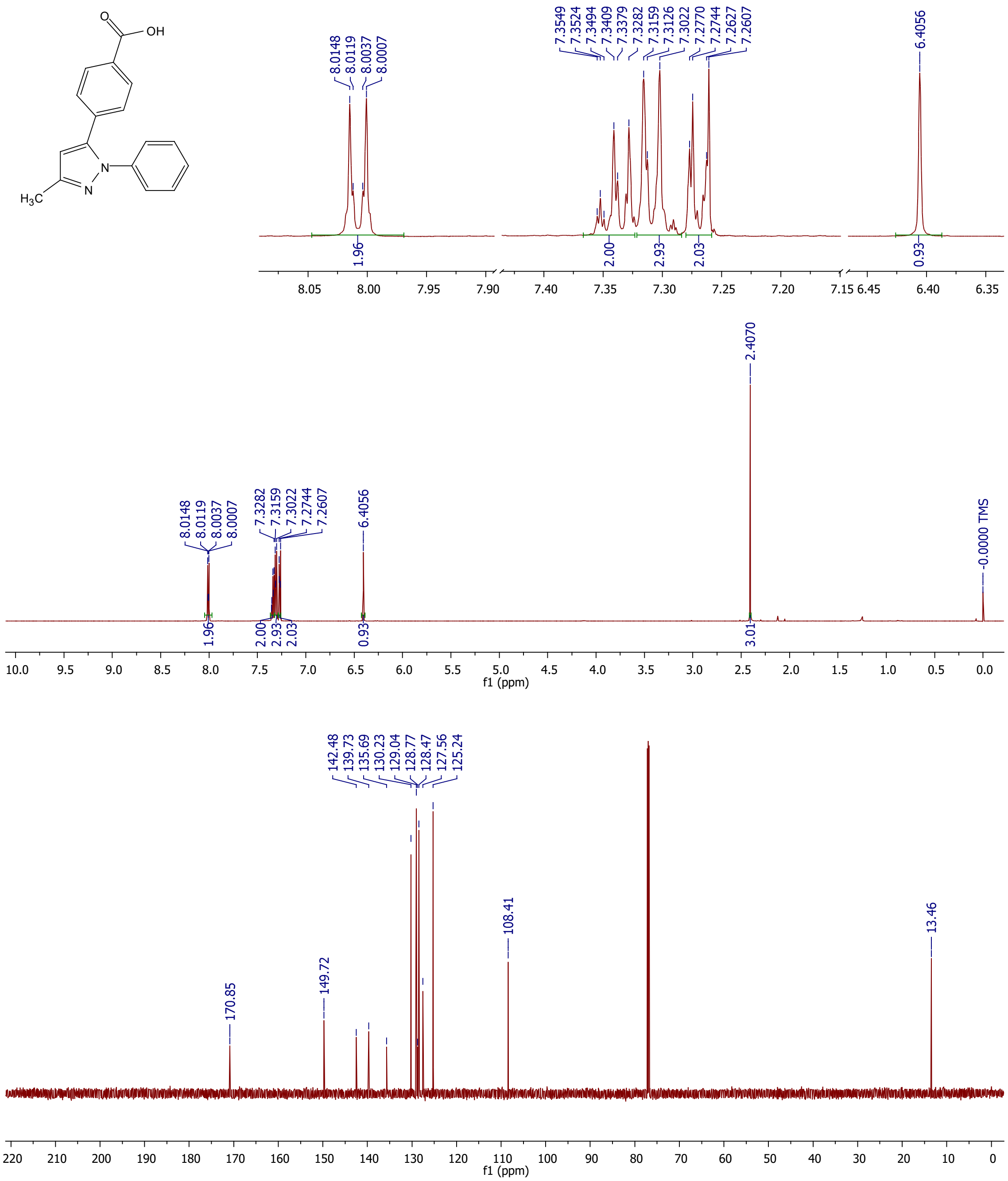

Figure S27. ^{1}H and ^{13}C NMR of *4-(3-methyl-1-phenyl-1H-pyrazol-5-yl)benzoic acid* **6c**

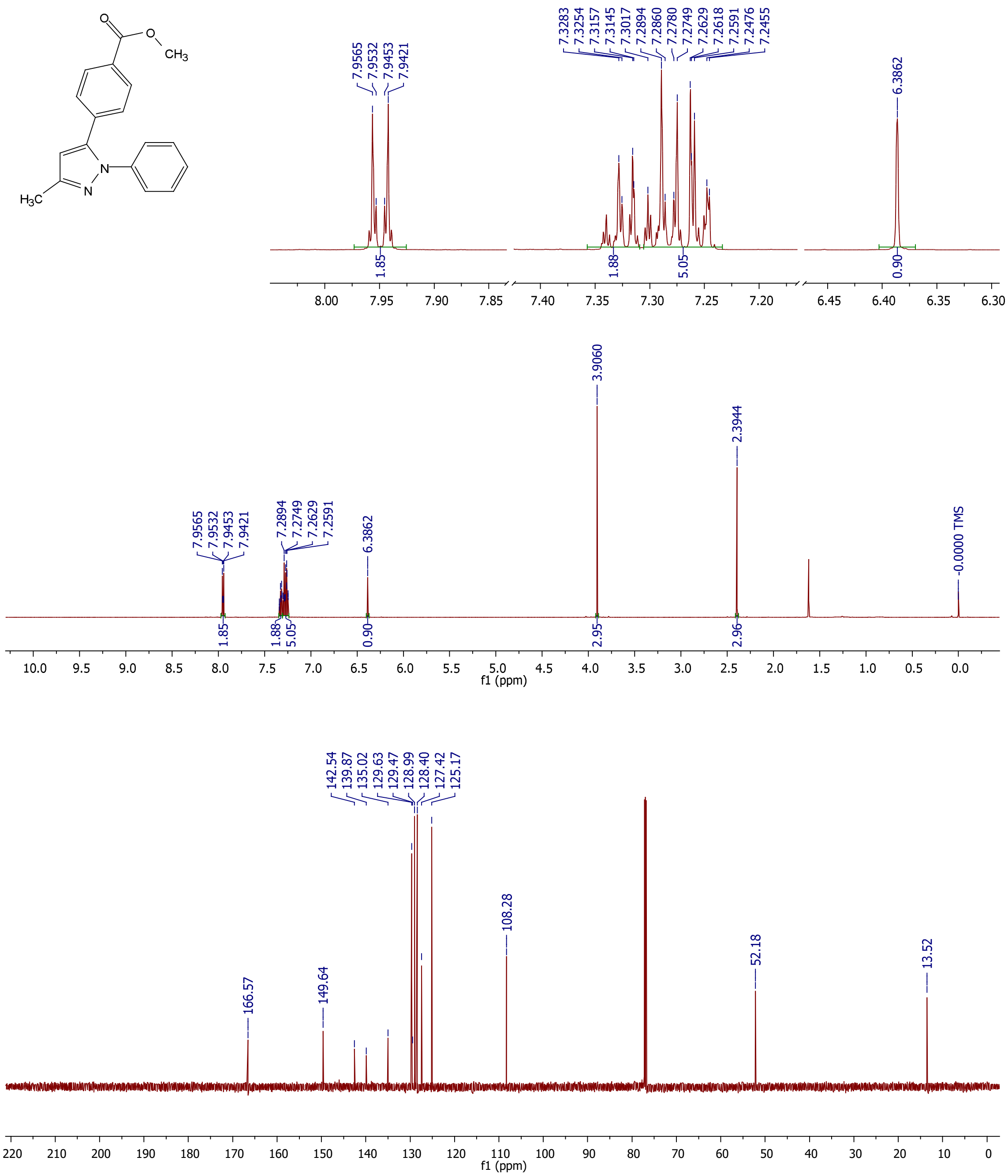

Figure S28. ¹H and ¹³C NMR of *methyl 4-(3-methyl-1-phenyl-1H-pyrazol-5-yl)benzoate* **6d**

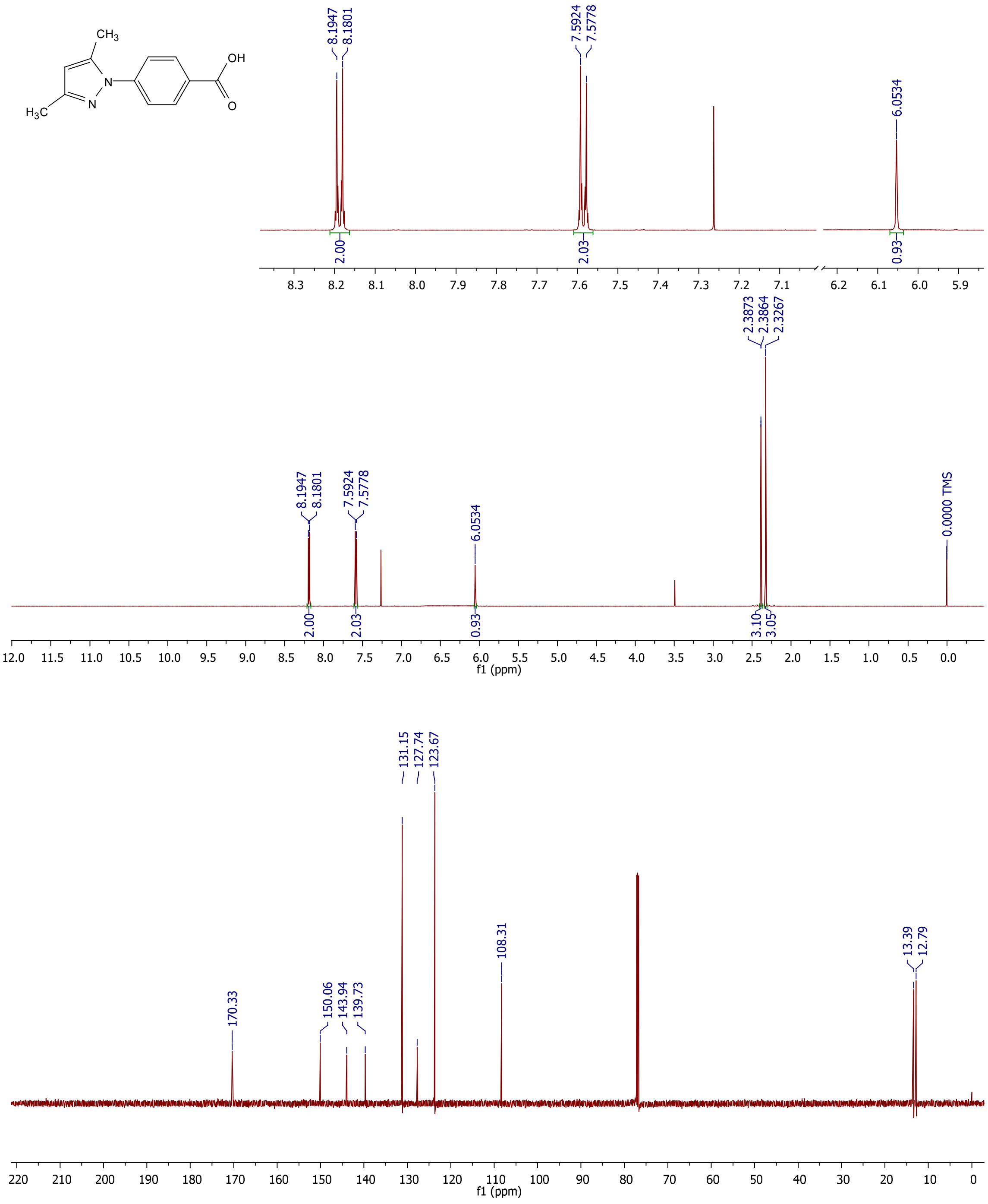

Figure S29. ^{1}H and ^{13}C NMR of *4-(3,5-dimethyl-1H-pyrazol-1-yl)benzoic acid* **6e.**

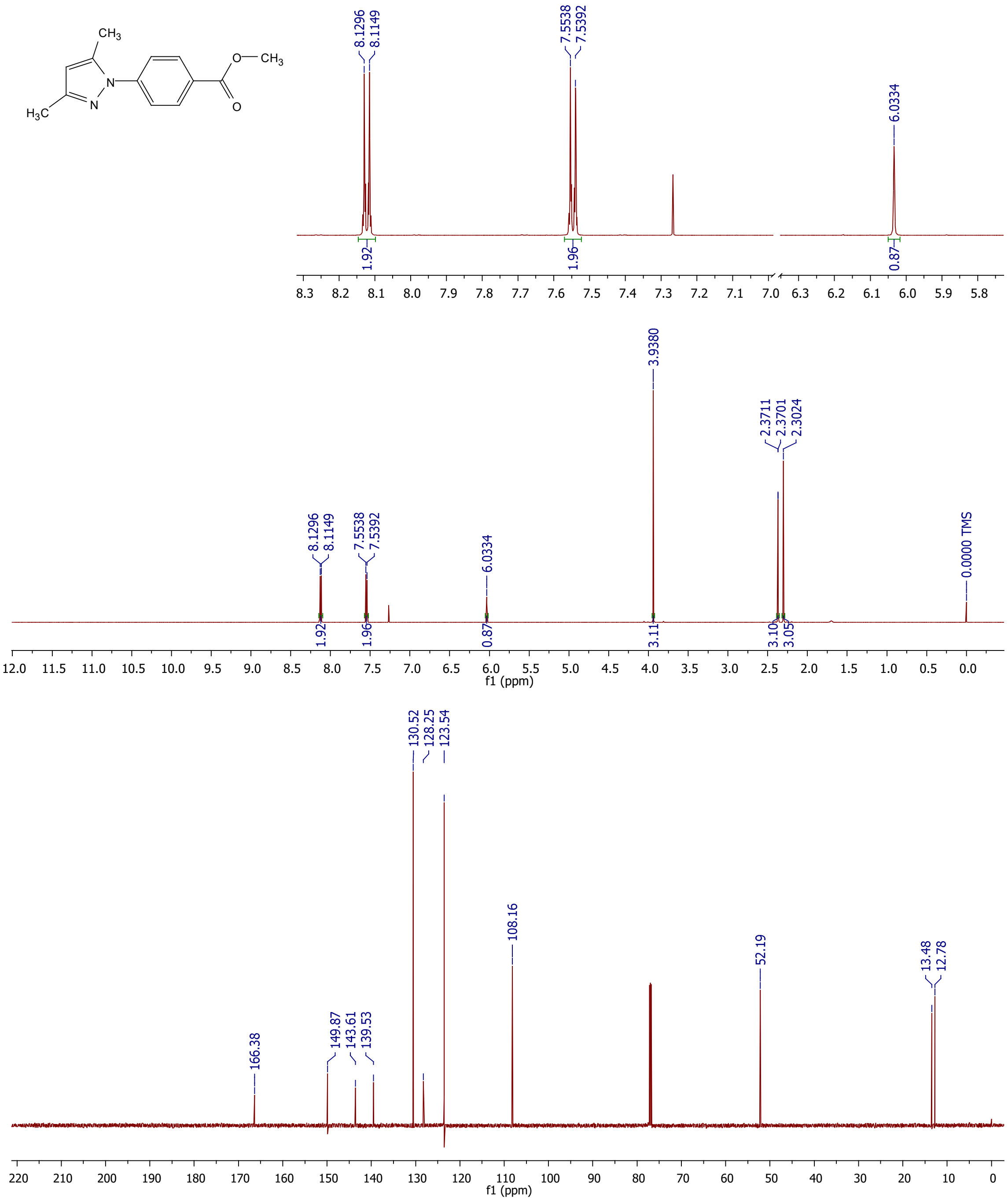

Figure S30. ^{1}H and ^{13}C NMR of *methyl 4-(3,5-dimethyl-1H-pyrazol-1-yl)benzoate* **6f.**

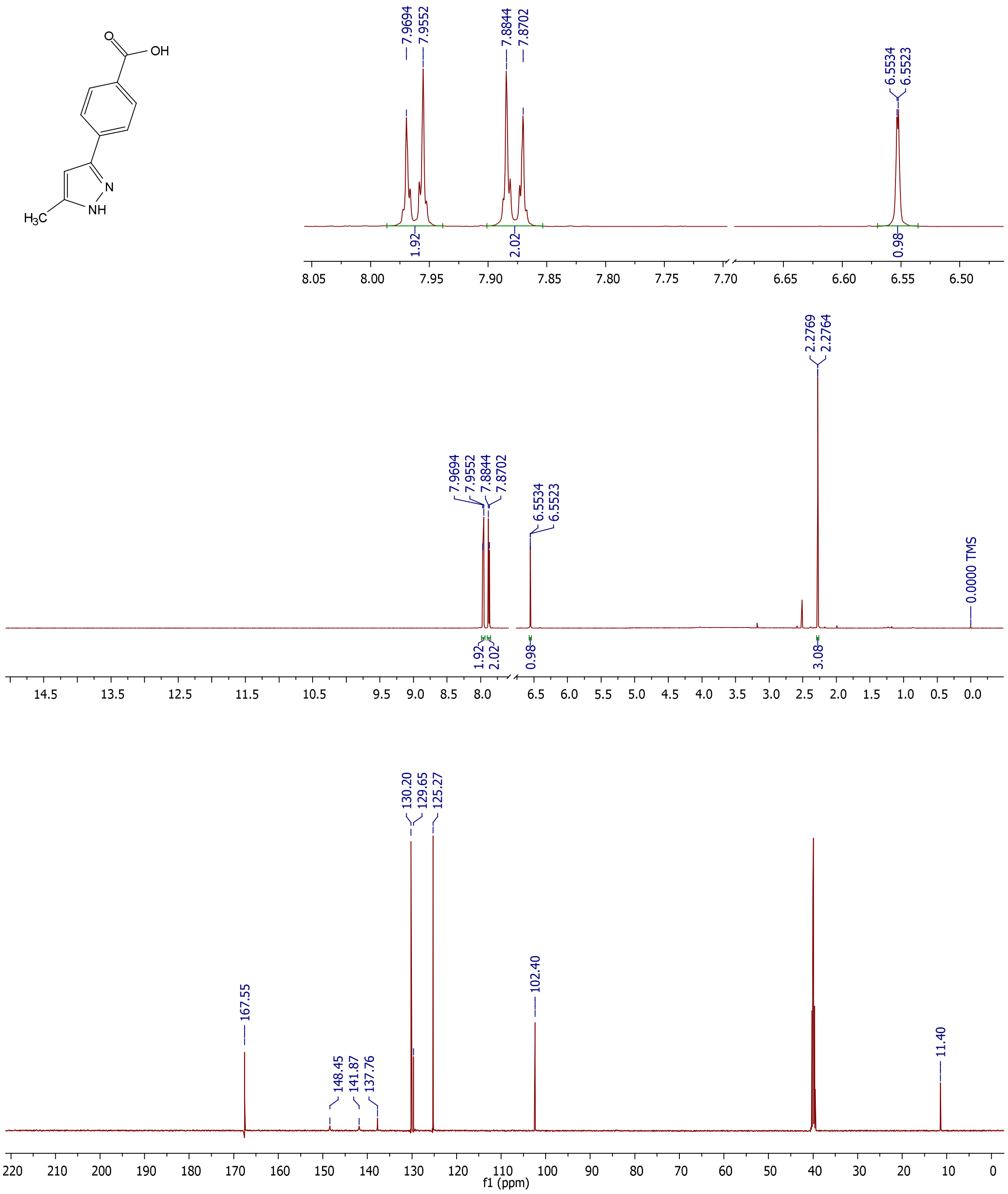

Figure S31. ^{1}H and ^{13}C NMR of *4-(5-methyl-1H-pyrazol-3-yl)benzoic acid* **6g.**

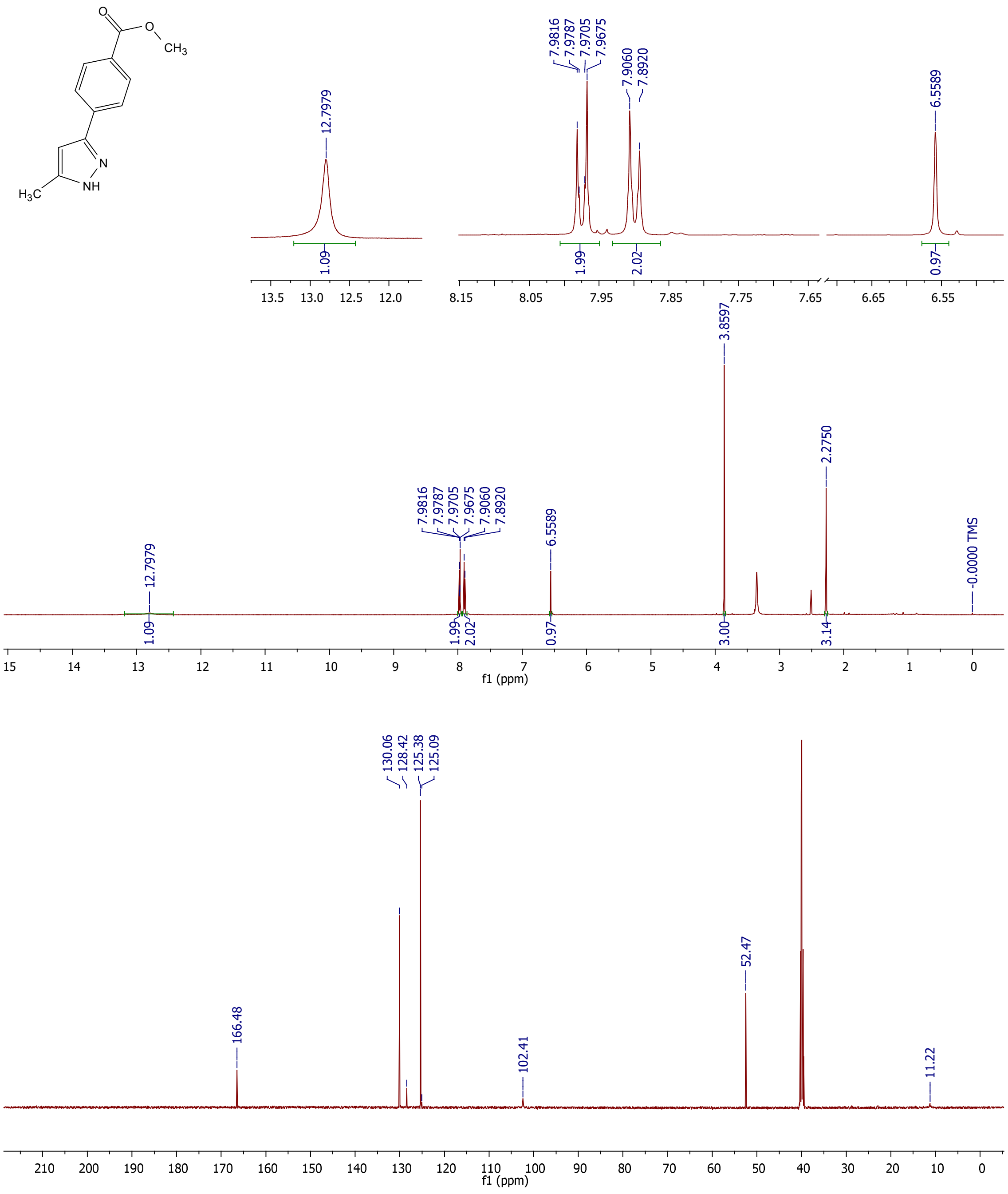

Figure S32. ^{1}H and ^{13}C NMR of *methyl 4-(3-methyl-1H-pyrazol-5-yl)benzoate* **6h.**

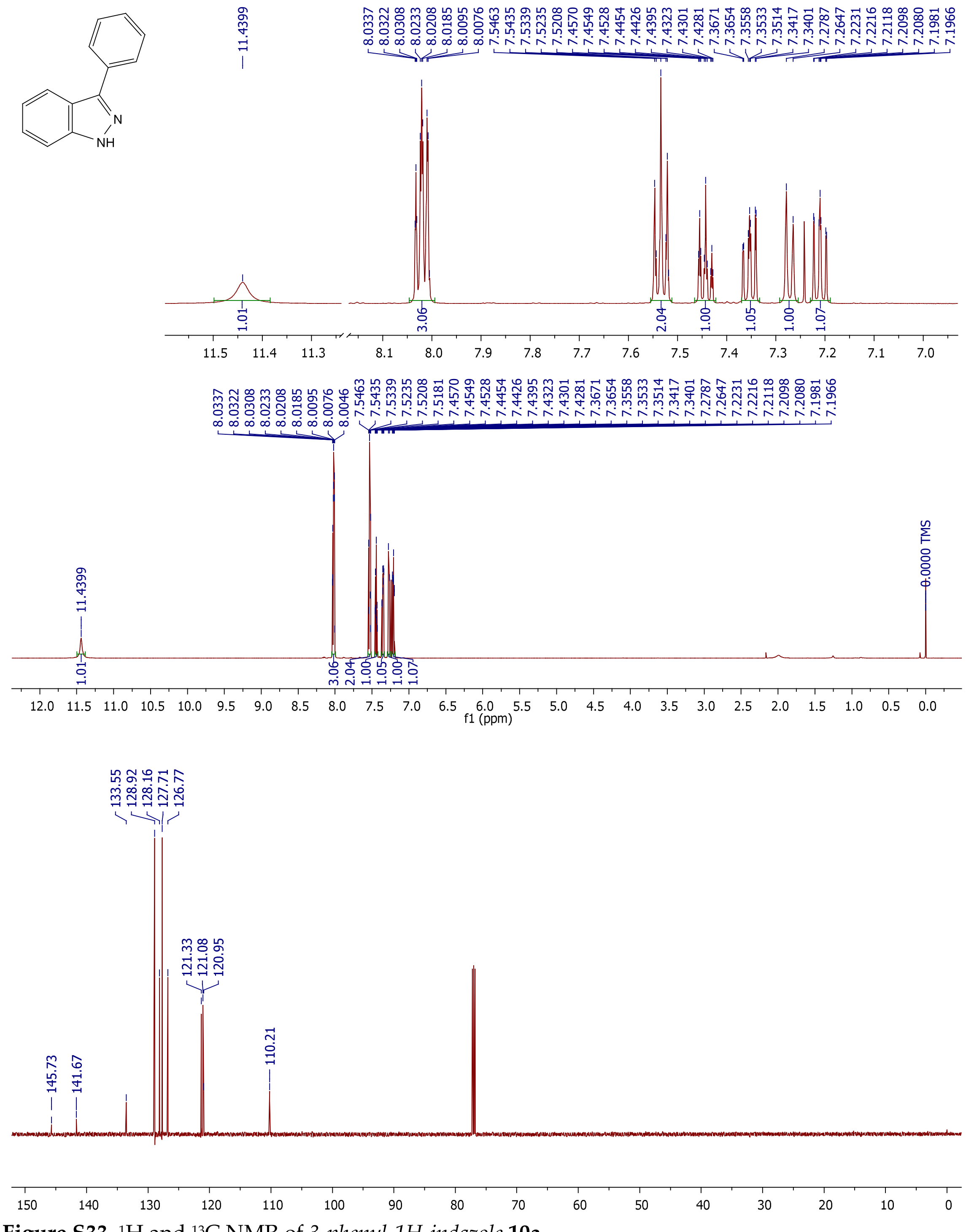

Figure S33. ^{1}H and ^{13}C NMR of *3-phenyl-1H-indazole* **10a**

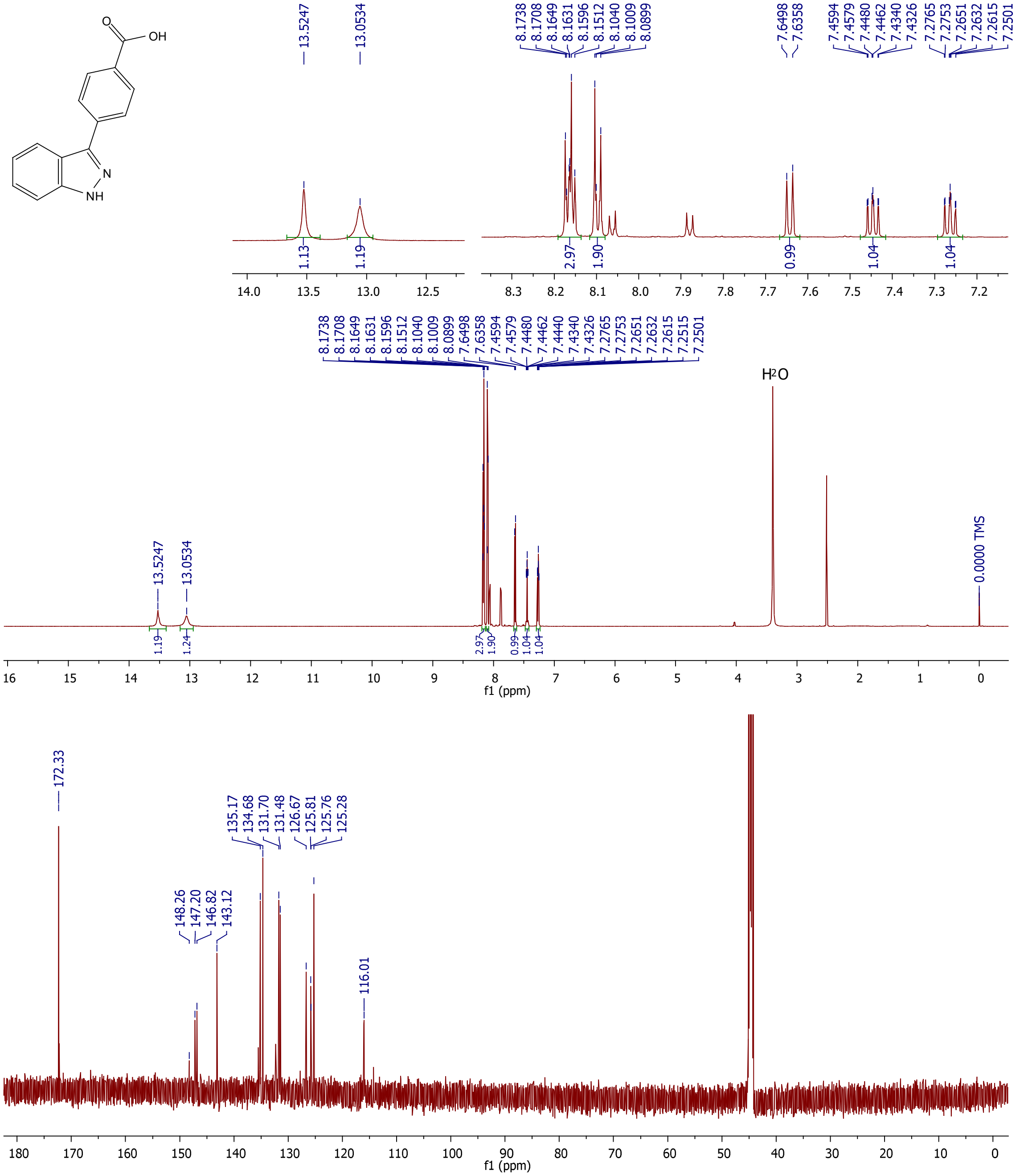

Figure S34. ^{1}H and ^{13}C NMR of *4-(1H-indazol-3-yl)benzoic acid* **10b.**

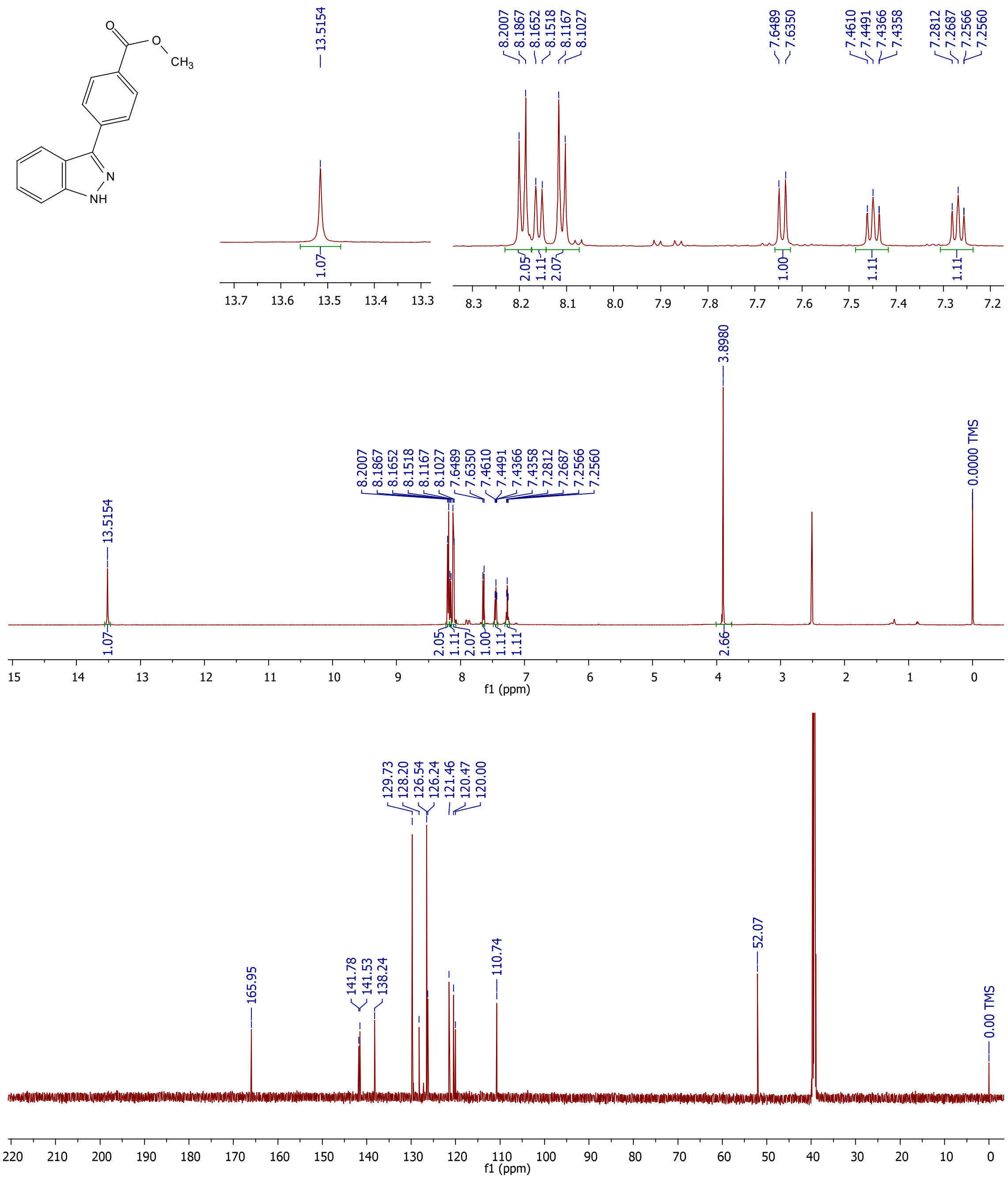

Figure S35. ^{1}H and ^{13}C NMR of *methyl 4-(1H-indazol-3-yl)benzoate* **10c**.

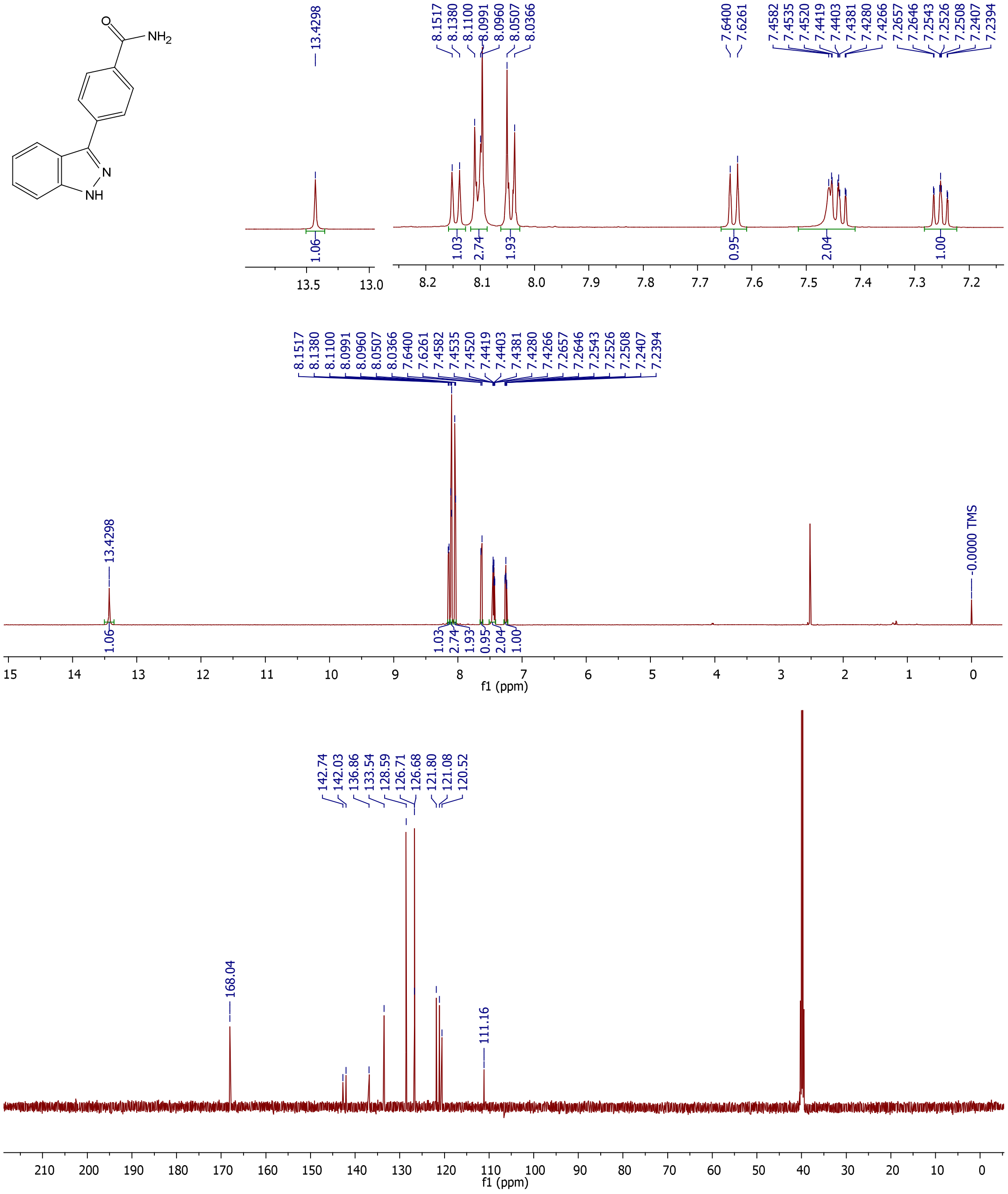

Figure S36. ^{1}H and ^{13}C NMR of *4-(1H-indazol-3-yl)benzamide* **10d**.

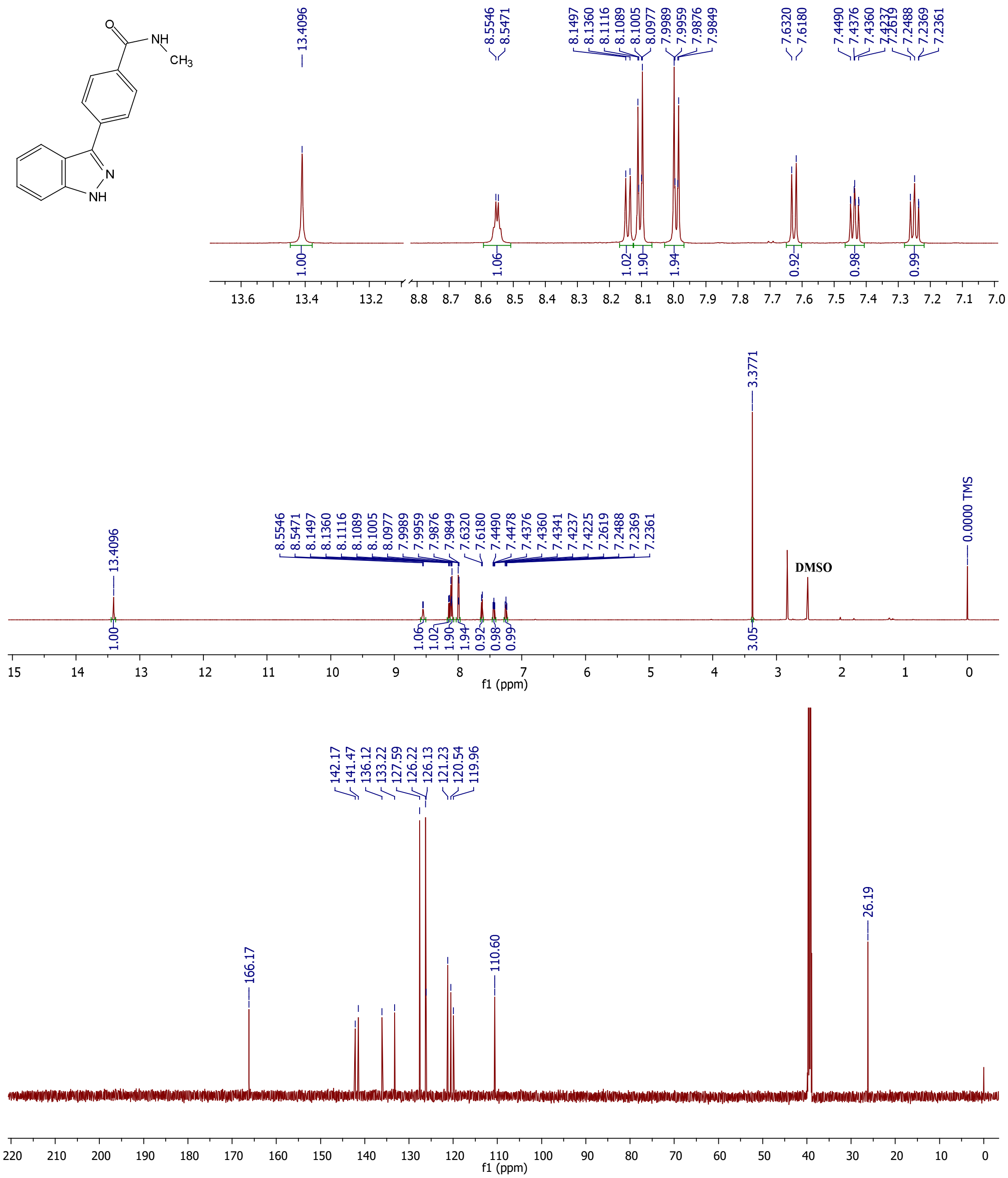

Figure S37. ^{1}H and ^{13}C NMR of *4-(1H-indazol-3-yl)-N-methylbenzamide* **10e**

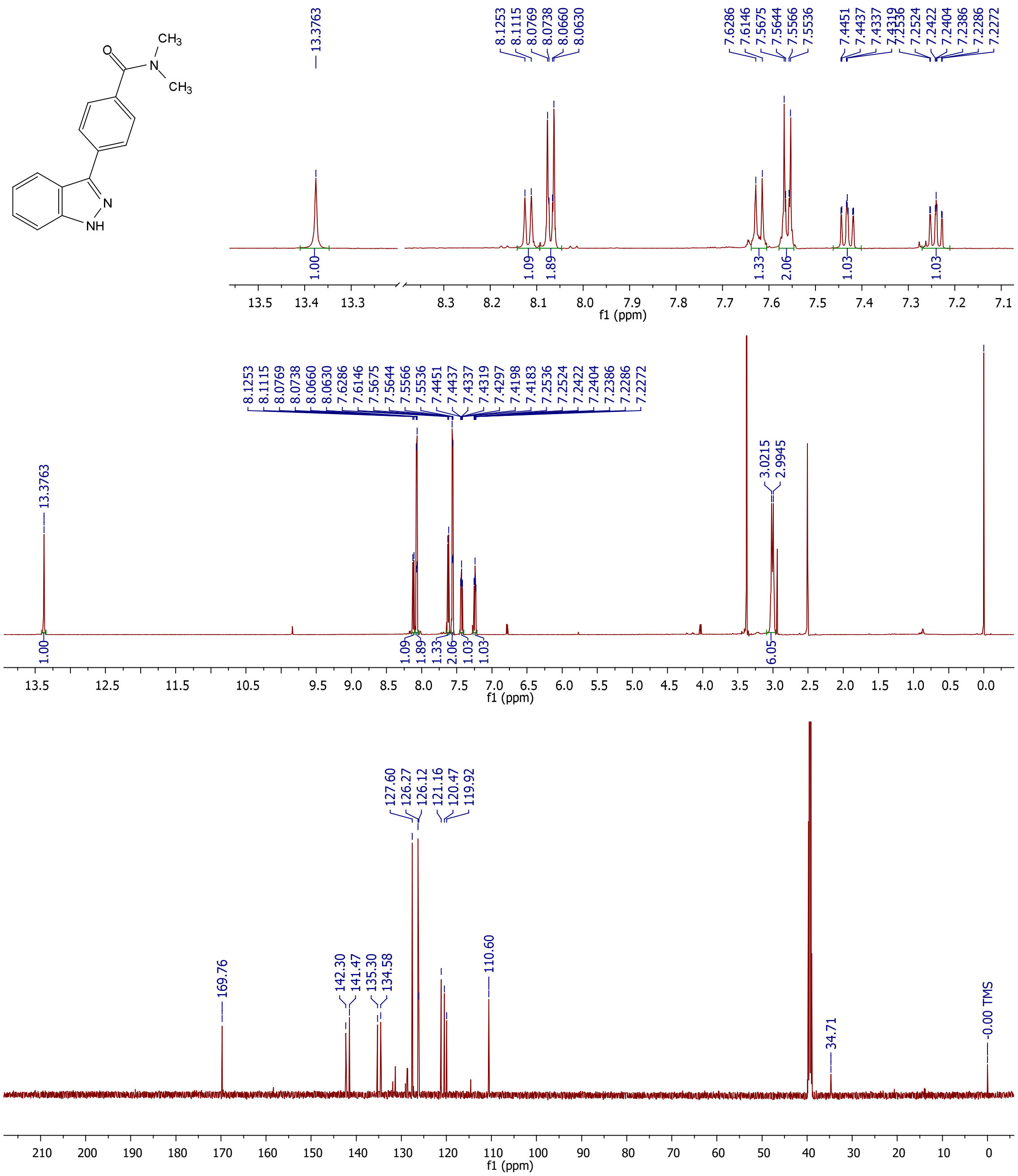

Figure S38. ^{1}H and ^{13}C NMR of *4-(1H-Indazol-3-yl)-N,N-dimethylbenzamide* **10f**.

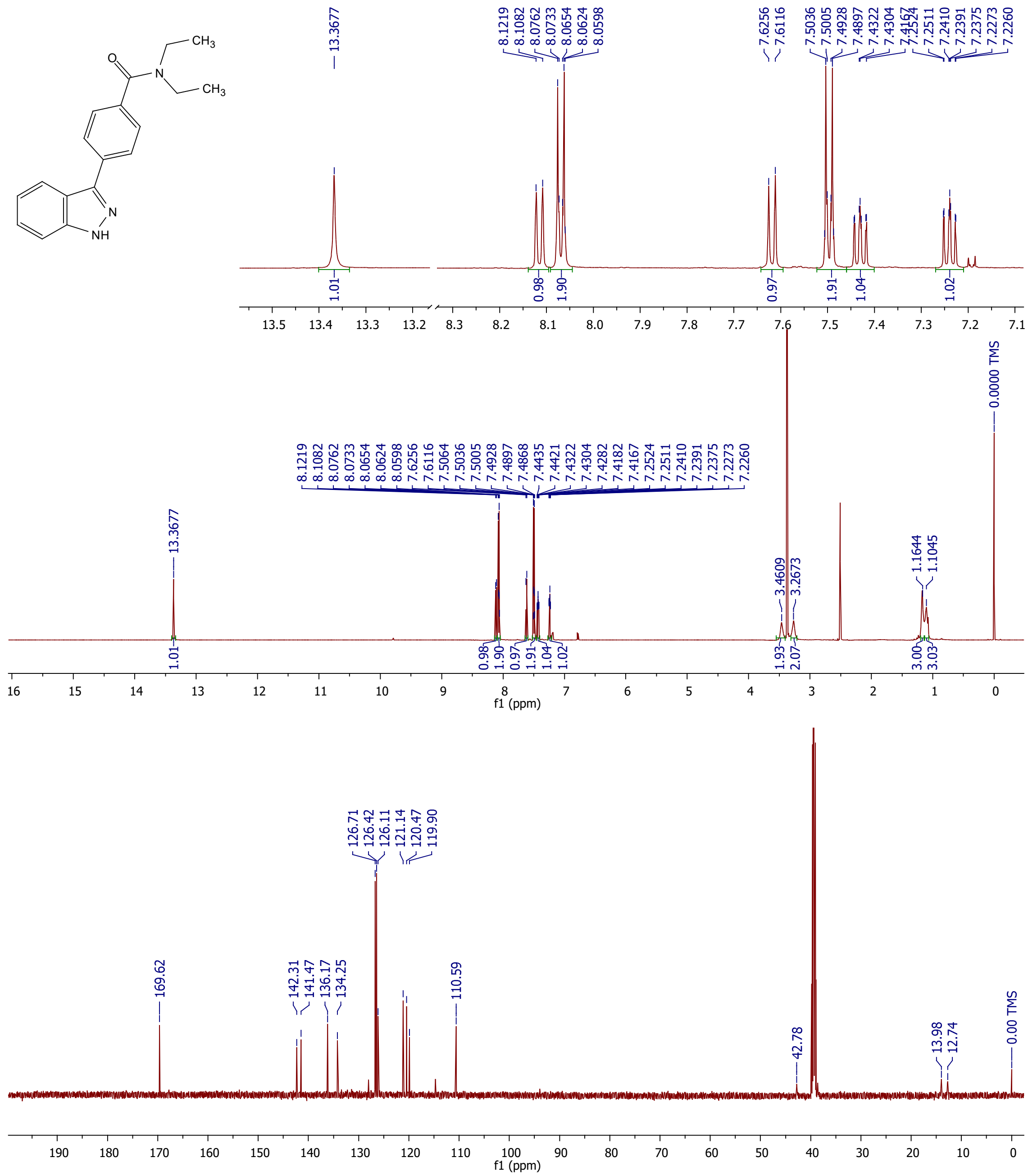

Figure S39. ^{1}H and ^{13}C NMR of *N,N-diethyl-4-(1H-indazol-3-yl)benzamide* **10g**.

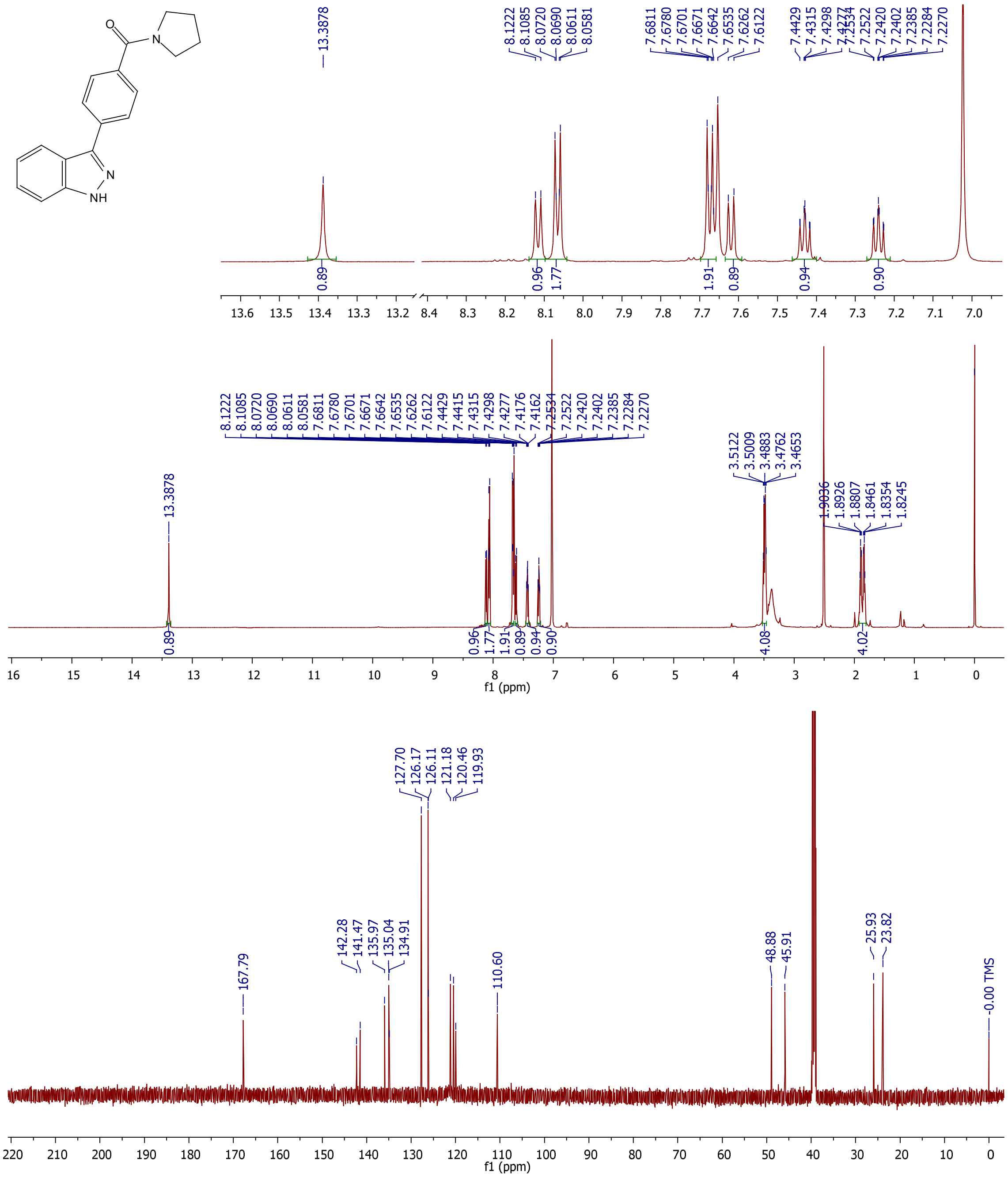

Figure S40. ^{1}H and ^{13}C NMR of *(4-(1H-indazol-3-yl)phenyl)(pyrrolidin-1-yl)methanone* **10h**

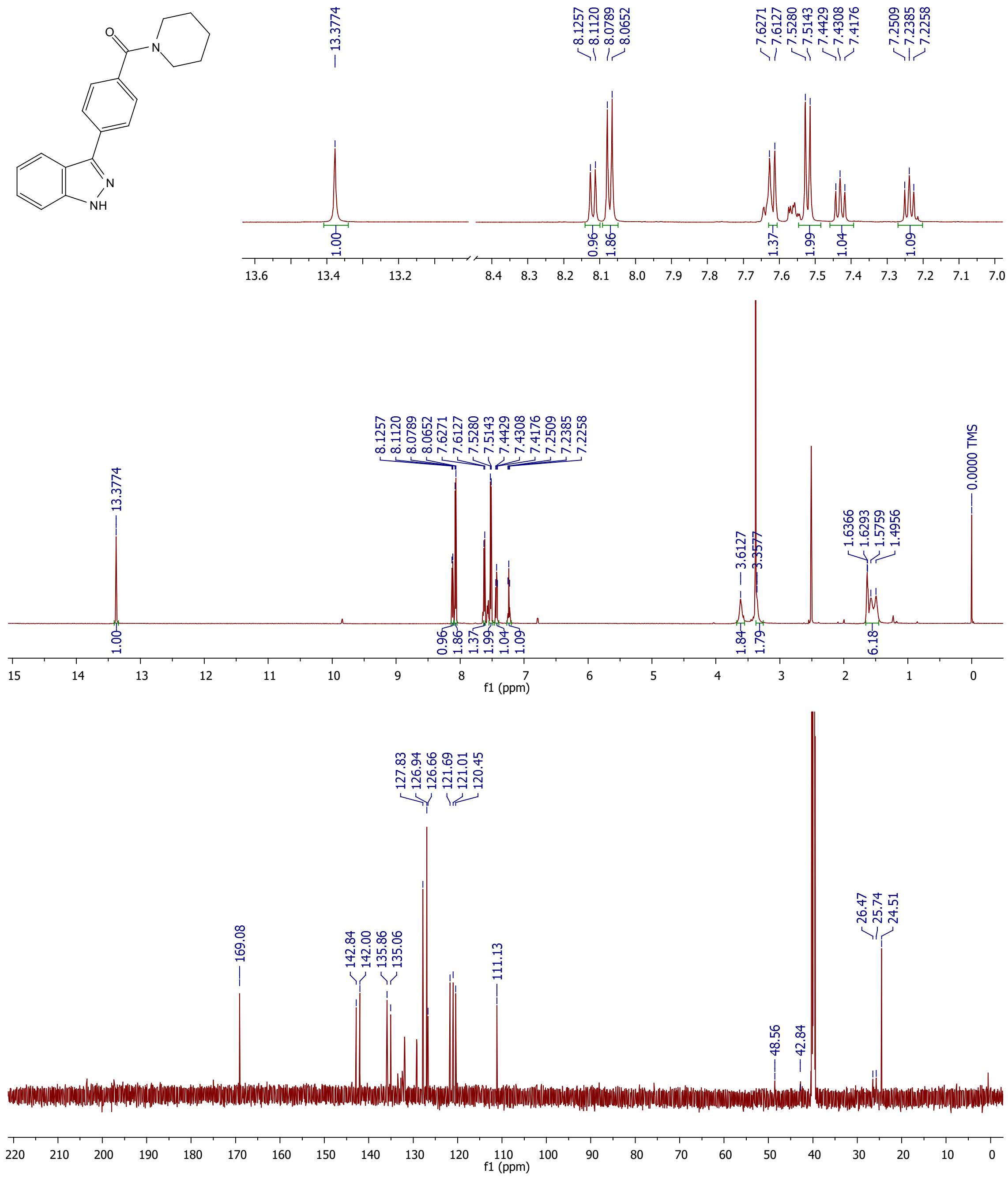

Figure S41. ^{1}H and ^{13}C NMR of *(4-(1H-indazol-3-yl)phenyl)(piperidin-1-yl)methanone* **10i.**

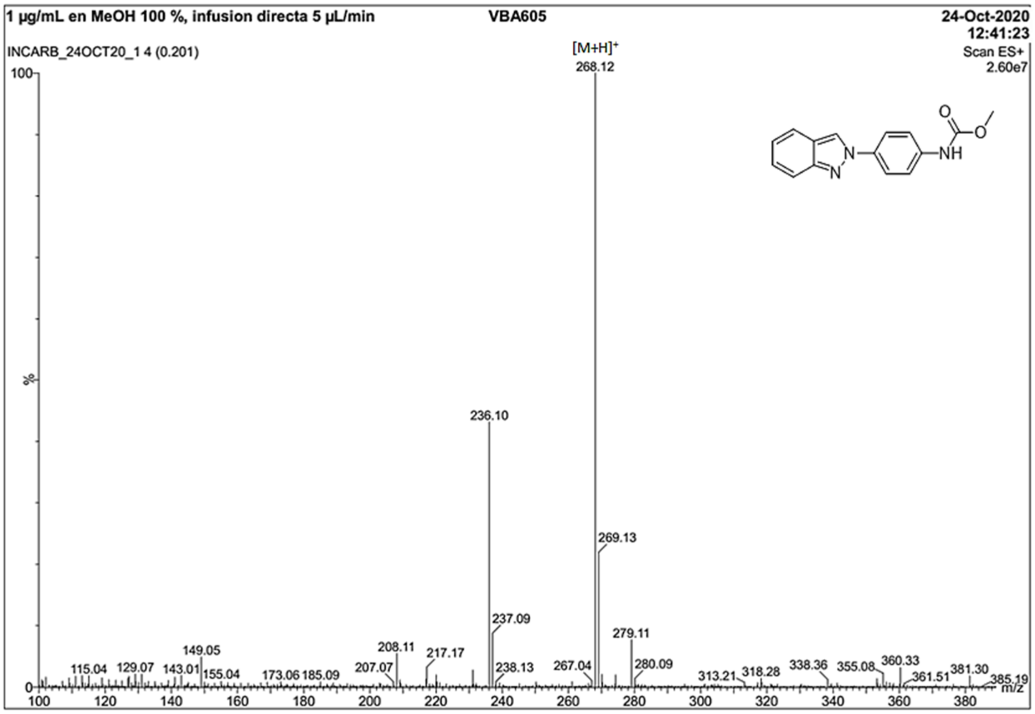

Figure S42. MS for *methyl (4-(2H-indazol-2-yl)phenyl)carbamate* **2d**.

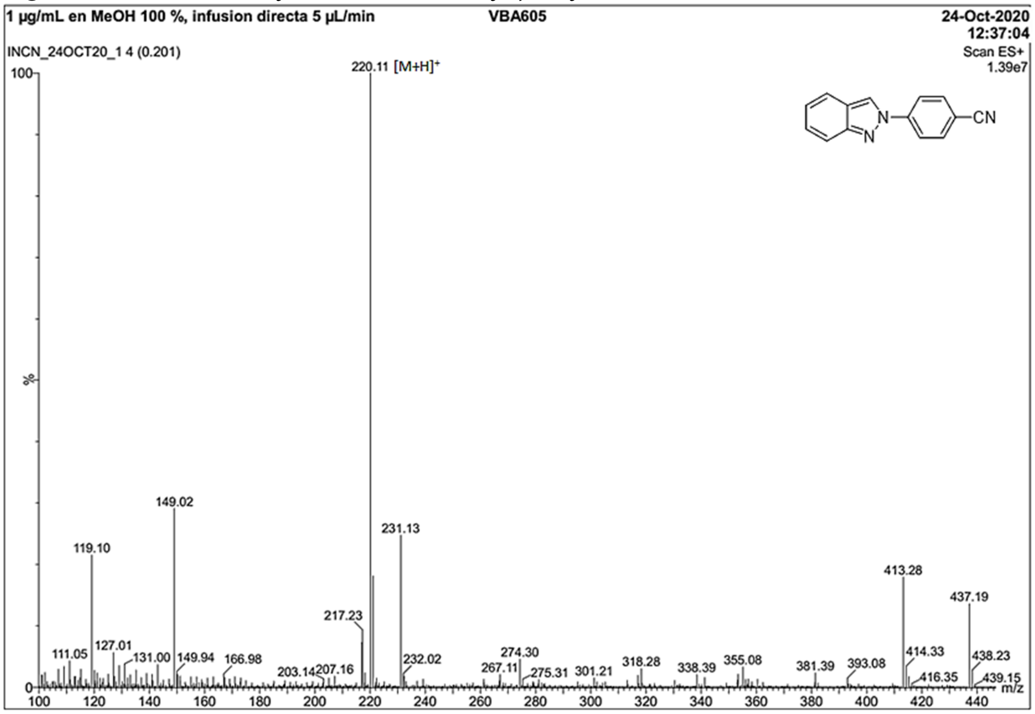

Figure S43. MS for *4-(2H-indazol-2-yl)benzonitrile* **2e**.

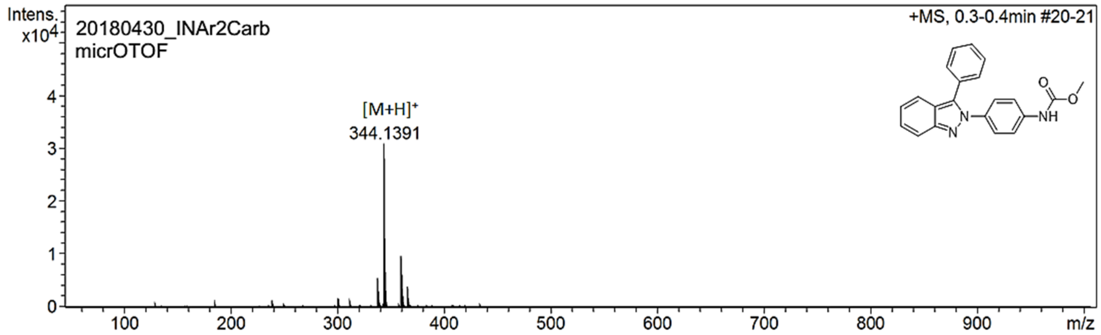

Figure S44. MS for *Methyl (4-(3-phenyl-2H-indazol-2-yl)phenyl)carbamate* **3e**.

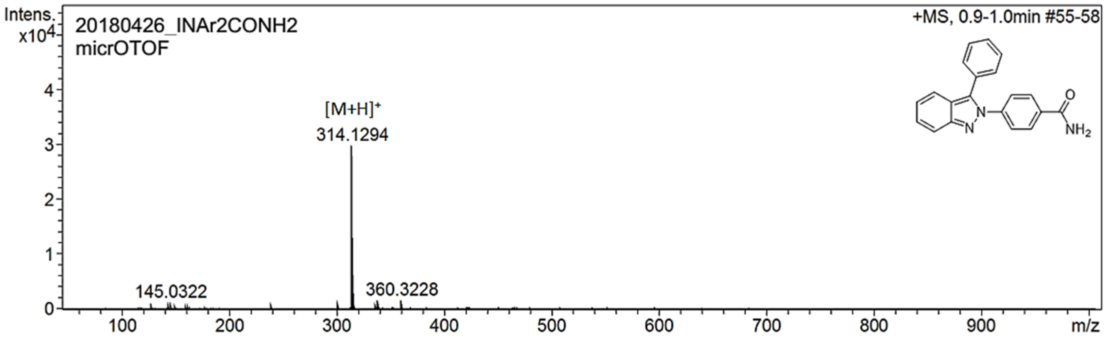

Figure S45. MS for *4-(3-Phenyl-2H-indazol-2-yl)benzamide* **3f**.

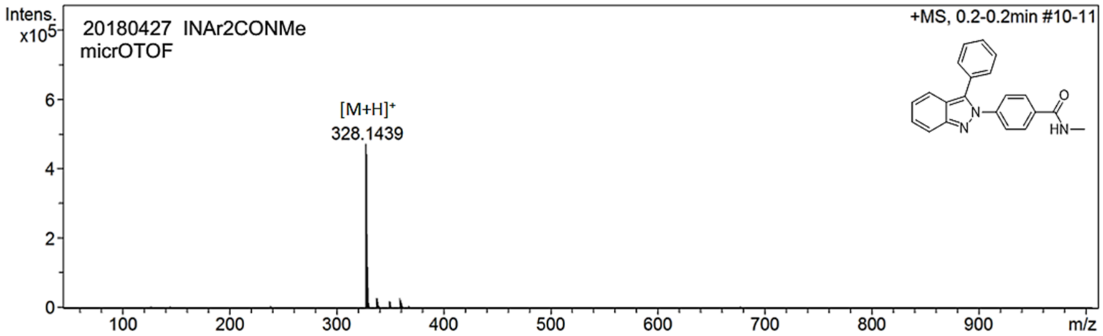

Figure S46. MS for *N-Methyl-4-(3-phenyl-2H-indazol-2-yl)benzamide* **3g**.

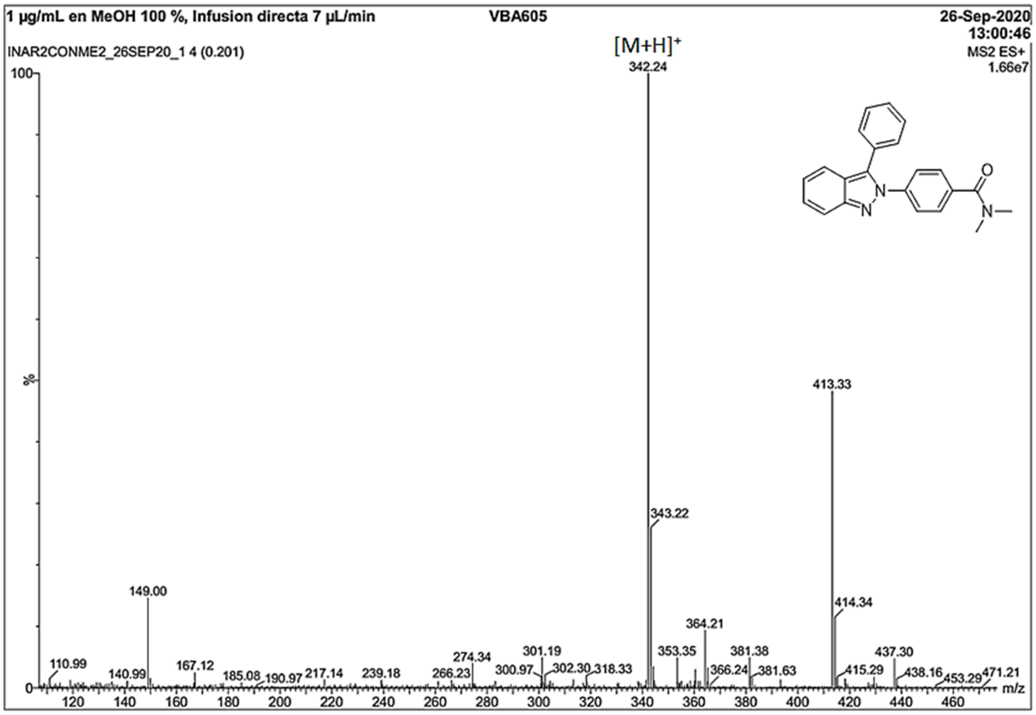

Figure S47. MS for *N,N-Dimethyl-4-(3-phenyl-2H-indazol-2-yl)benzamide* **3h**.

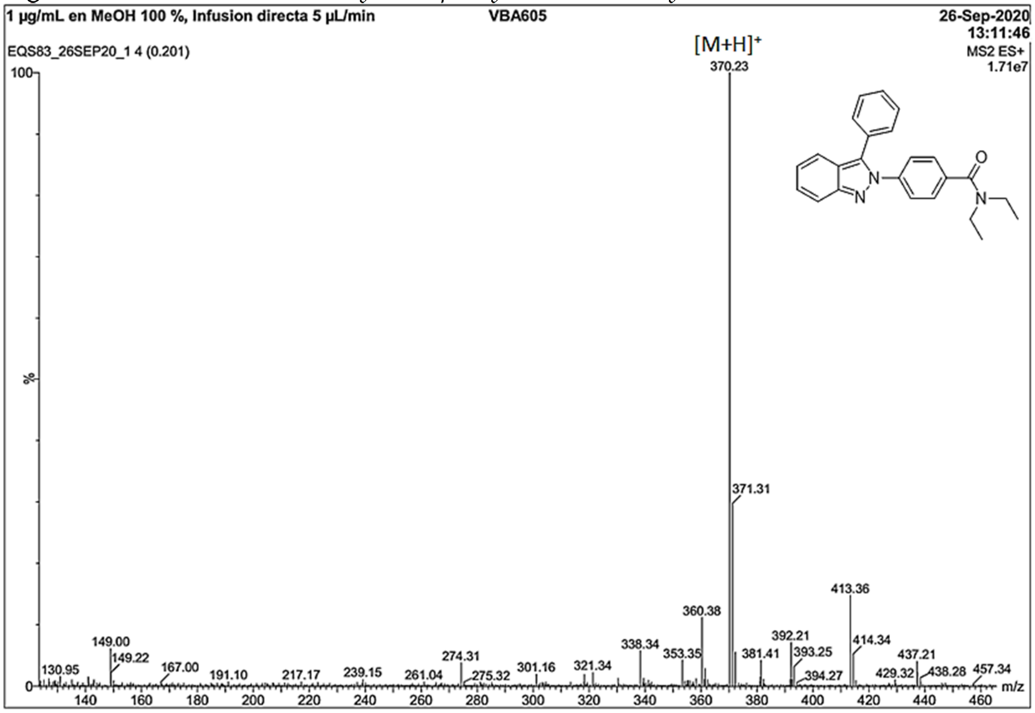

Figure S48. MS for *N,N-Diethyl-4-(3-phenyl-2H-indazol-2-yl)benzamide* **3i**.

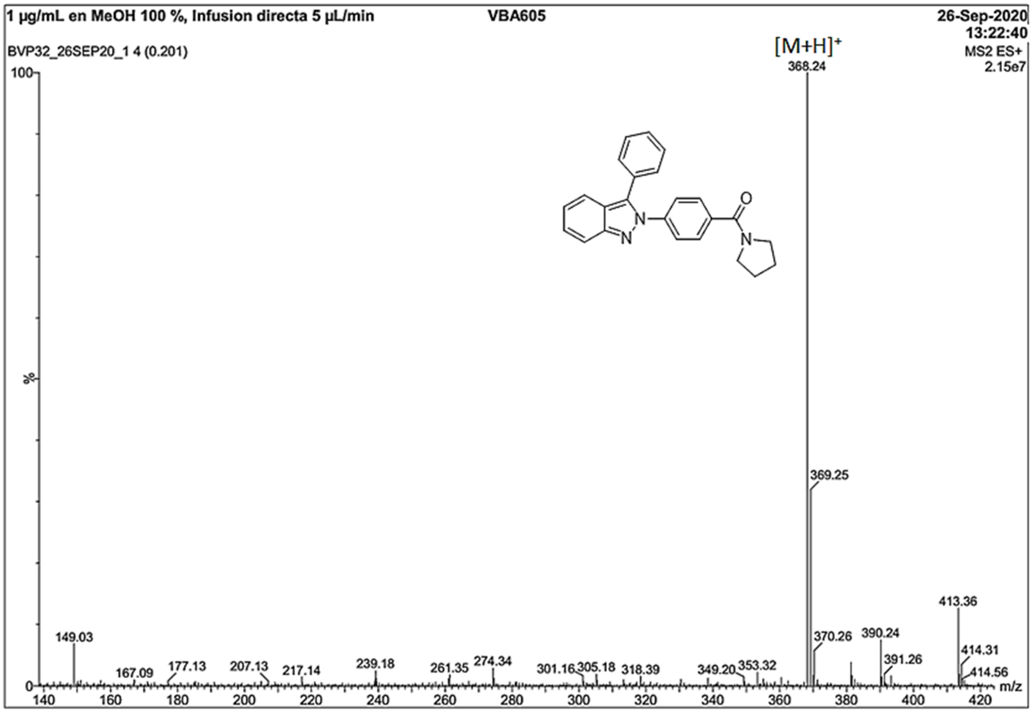

Figure S49. MS for *(4-(3-Phenyl-2H-indazol-2-yl)phenyl)(pyrrolidin-1-yl)methanone* **3j**.

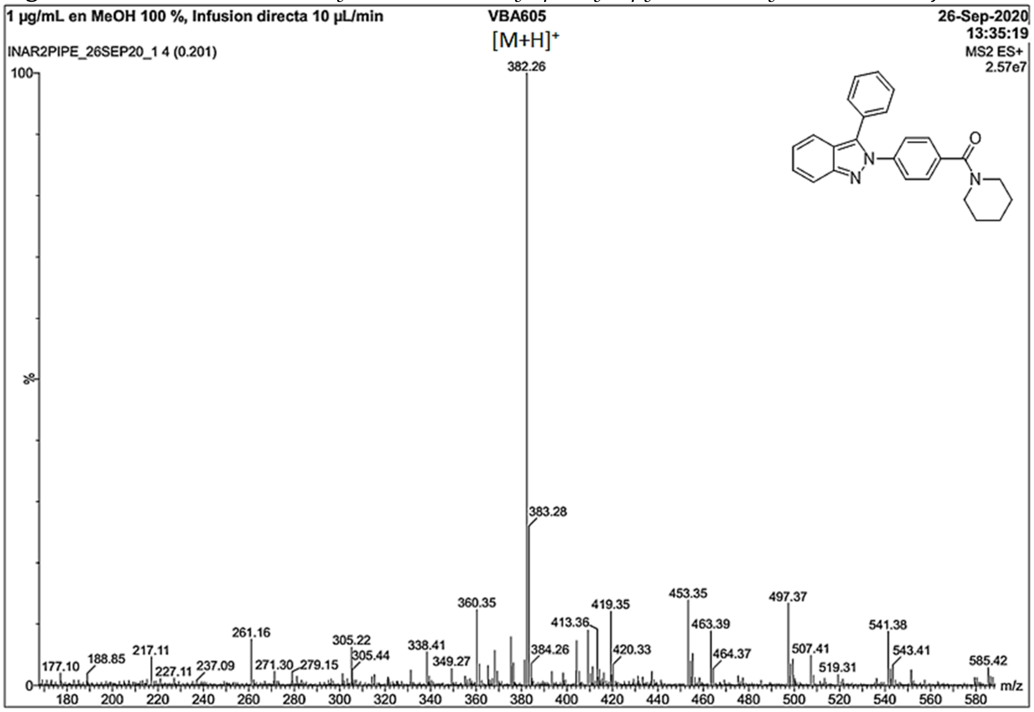

Figure S50. MS for *(4-(3-Phenyl-2H-indazol-2-yl)phenyl)(piperidin-1-yl)methanone* **3k**.

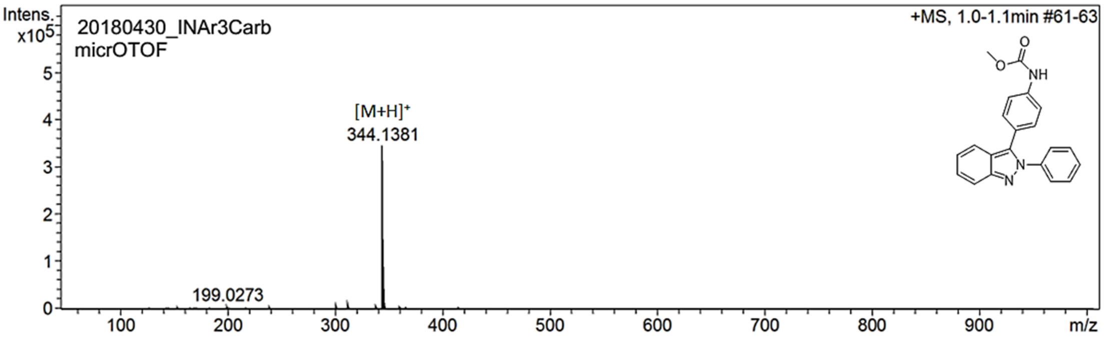

Figure S51. MS for *Methyl (4-(2-phenyl-2H-indazol-3-yl)phenyl)carbamate* **3l**.

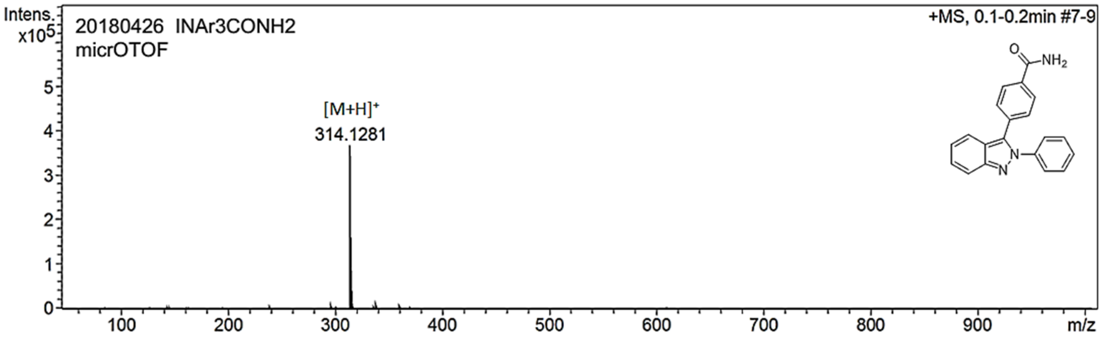

Figure S52. MS for *4-(2-Phenyl-2H-indazol-3-yl)benzamide* **3m**.

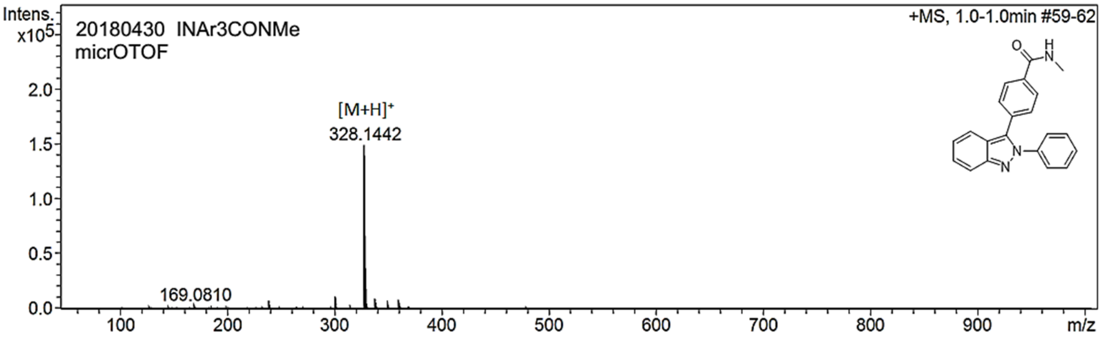

Figure S53. MS for *N-Methyl-4-(2-phenyl-2H-indazol-3-yl)benzamide* **3n**.

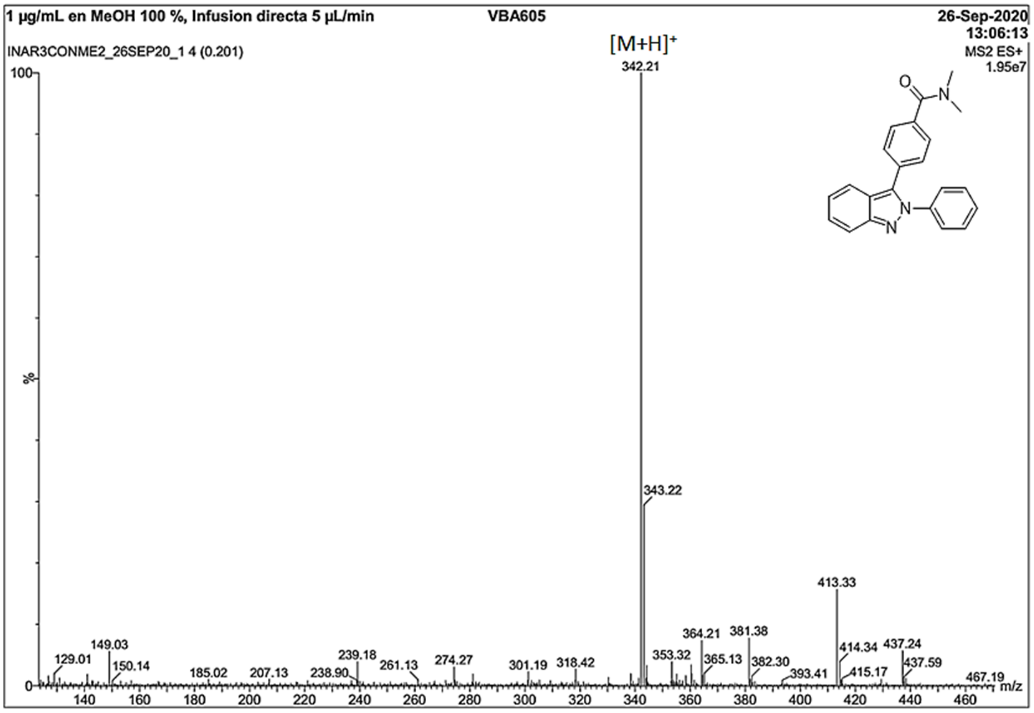

Figure S54. MS for *N,N-Dimethyl-4-(2-phenyl-2H-indazol-3-yl)benzamide* **3o**.

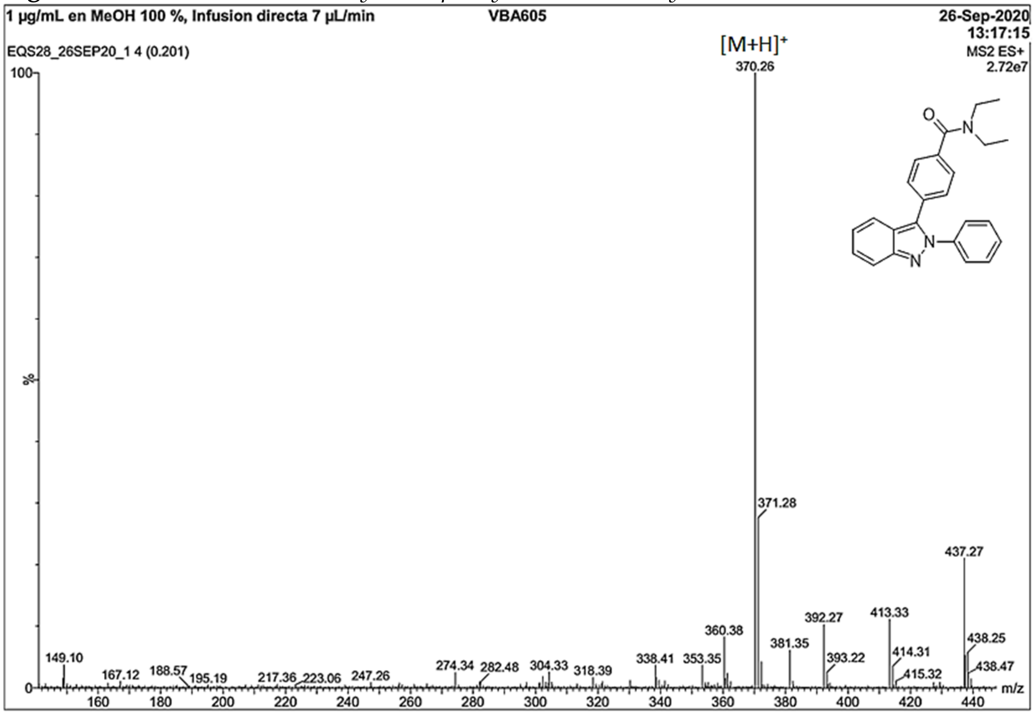

Figure S55. MS for *N,N-Diethyl-4-(2-phenyl-2H-indazol-3-yl)benzamide* **3p**.

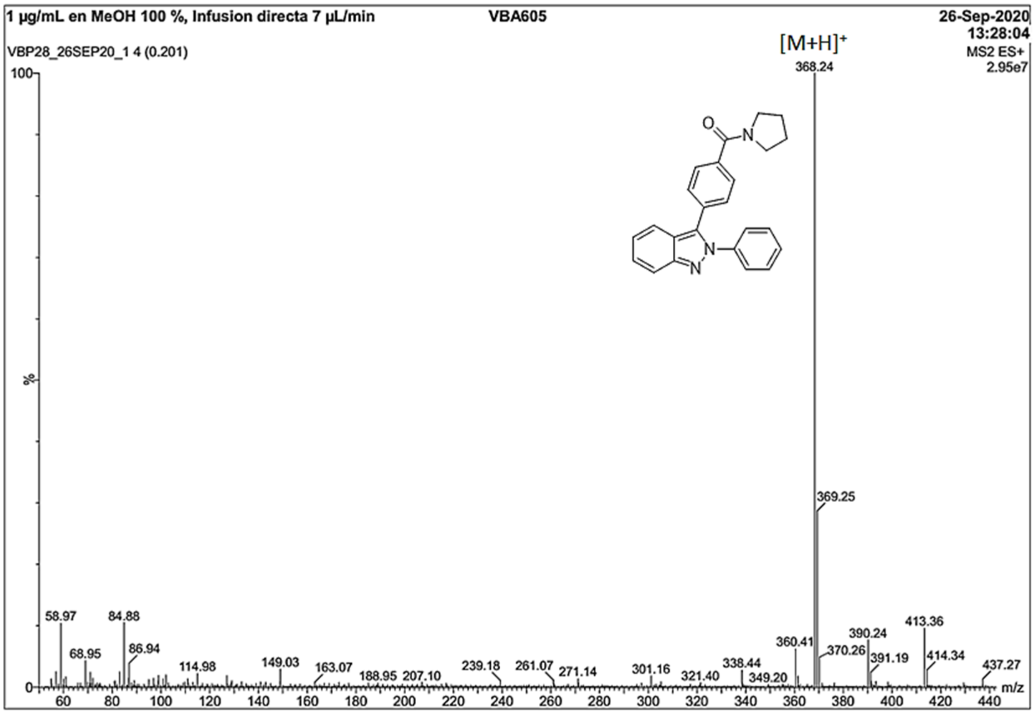

Figure S56. MS for *(4-(2-Phenyl-2H-indazol-3-yl)phenyl)(pyrrolidin-1-yl)methanone* **3q**.

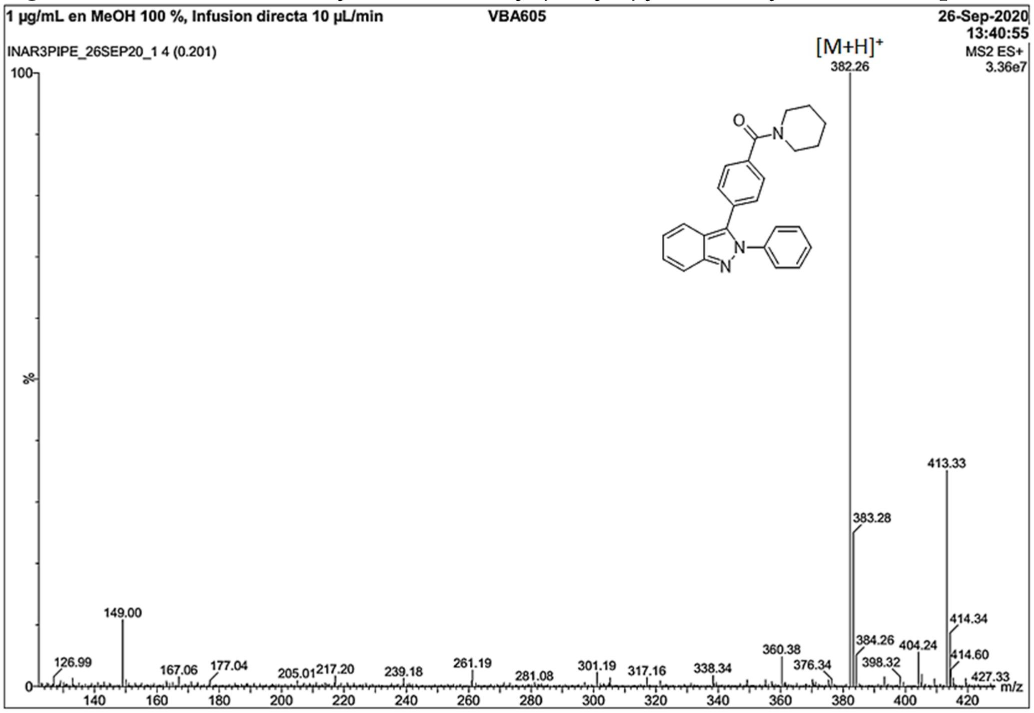

Figure S57. MS for *(4-(2-Phenyl-2H-indazol-3-yl)phenyl)(piperidin-1-yl)methanone* **3r**.

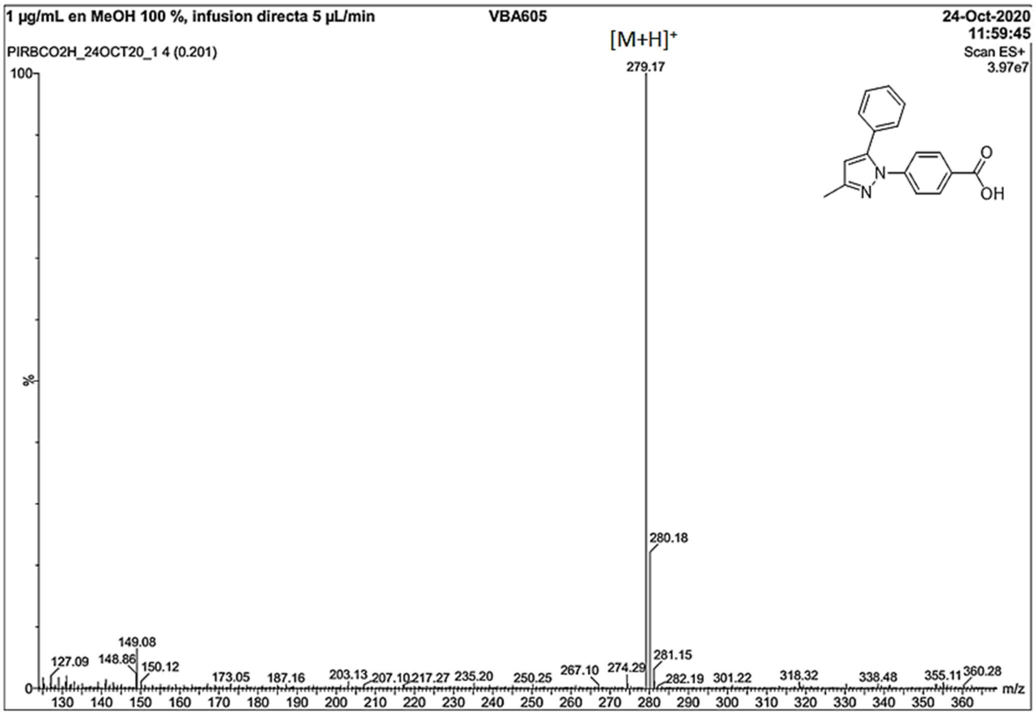

Figure S58. MS for *4-(3-Methyl-5-phenyl-1H-pyrazol-1-yl)benzoic acid* **6a**.

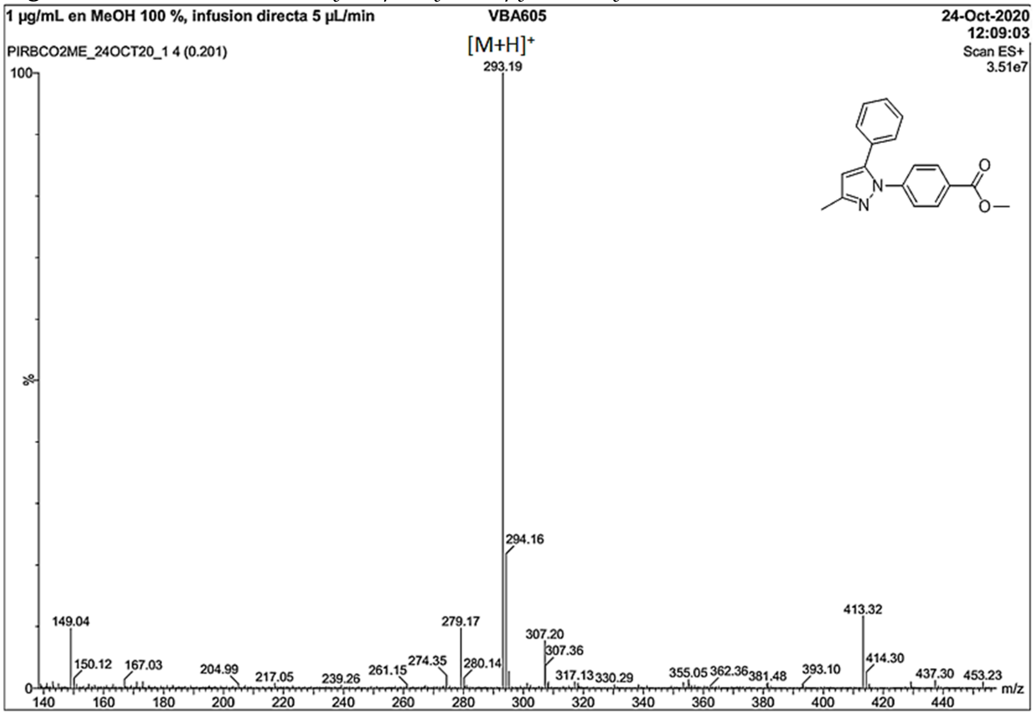

Figure S59. MS for *Methyl 4-(3-methyl-5-phenyl-1H-pyrazol-1-yl)benzoate* **6b**.

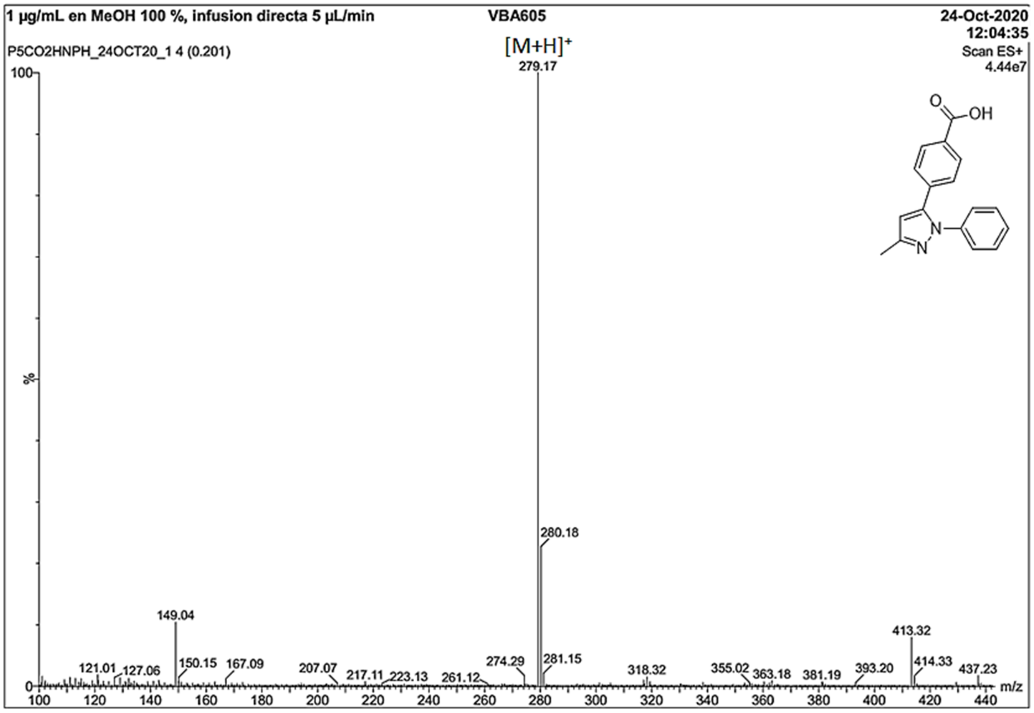

Figure S60. MS for *4-(3-Methyl-1-phenyl-1H-pyrazol-5-yl)benzoic acid* **6c**.

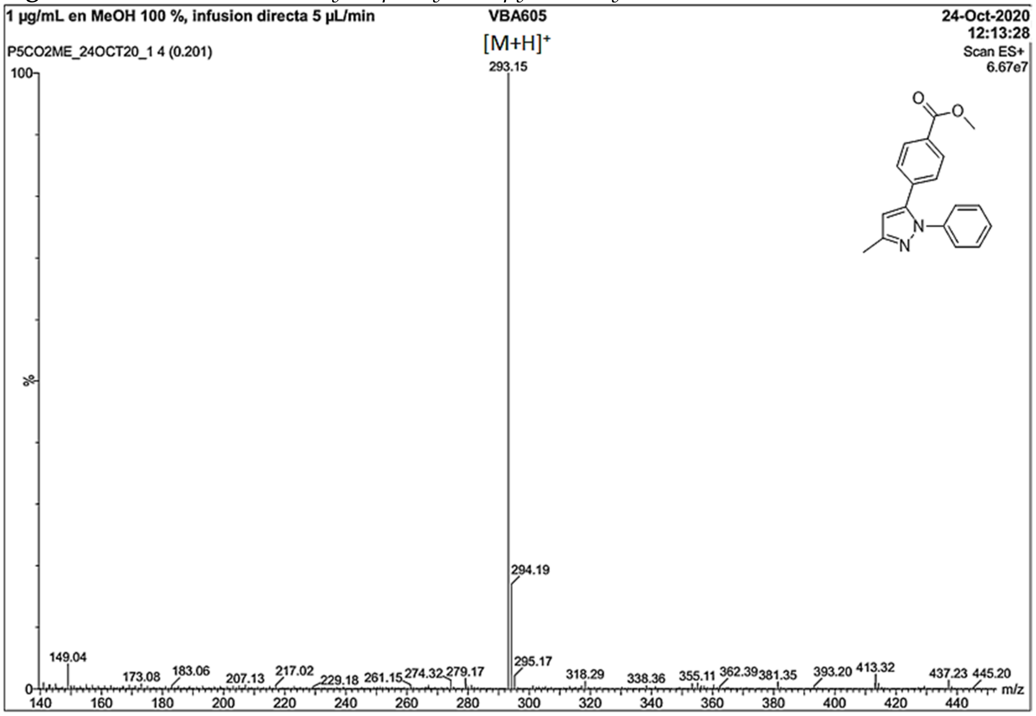

Figure S61. MS for *Methyl 4-(3-methyl-1-phenyl-1H-pyrazol-5-yl)benzoate* **6d**.

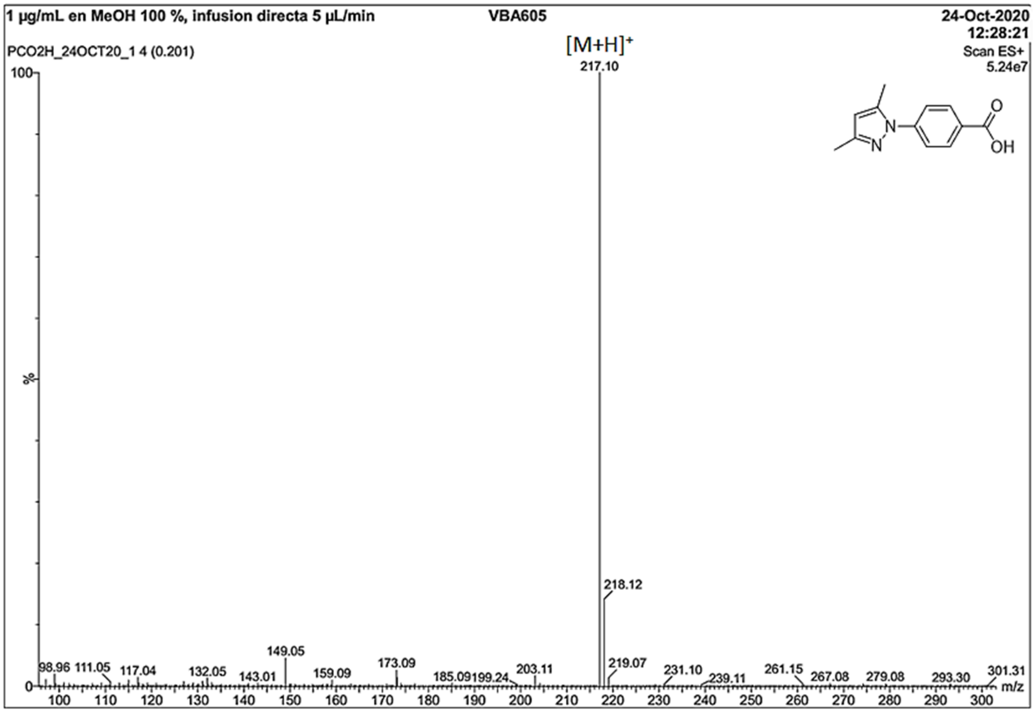

Figure S62. MS for *4-(3,5-Dimethyl-1H-pyrazol-1-yl)benzoic acid* **6e**.

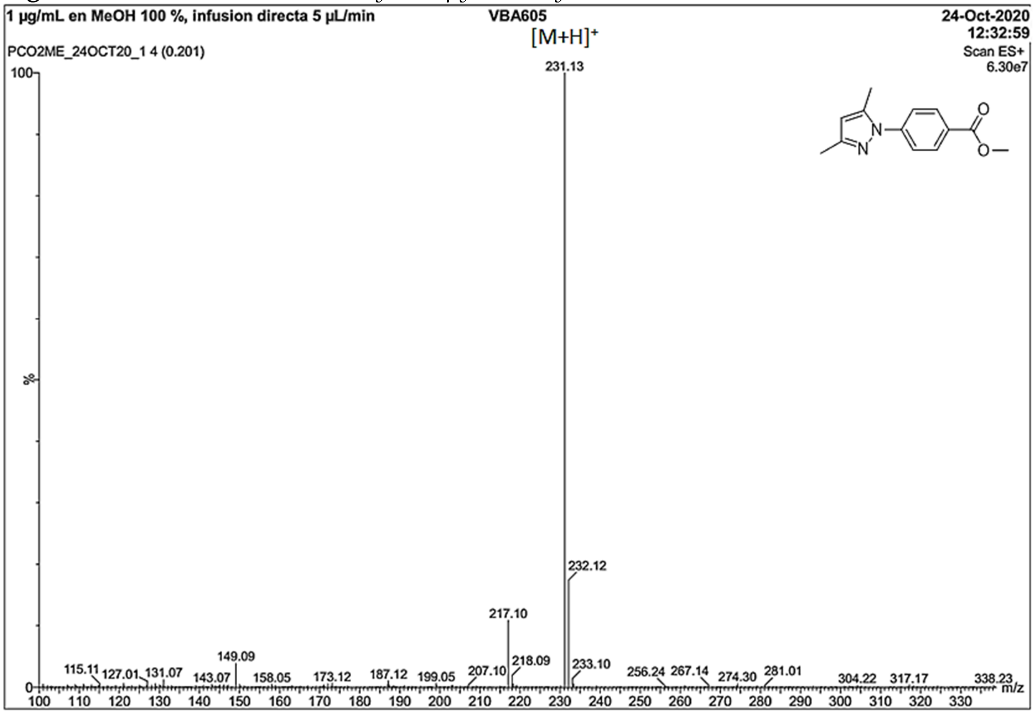

Figure S63. MS for *Methyl 4-(3,5-dimethyl-1H-pyrazol-1-yl)benzoate* **6f**.

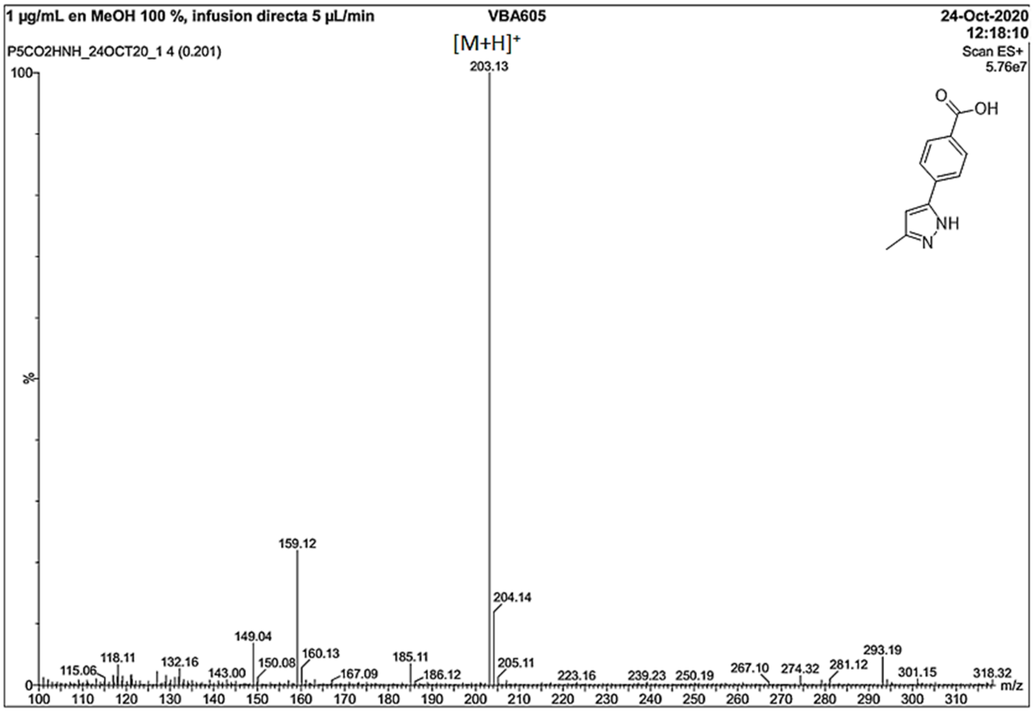

Figure S64. MS for *4-(5-Methyl-1H-pyrazol-3-yl)benzoic acid* **6g**.

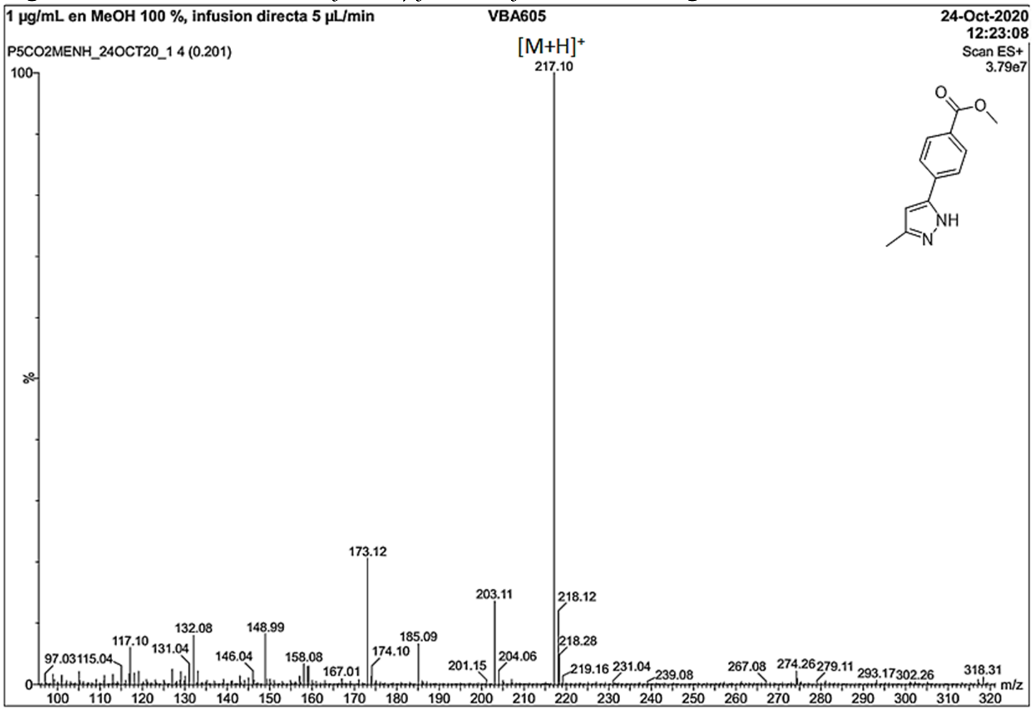

Figure S65. MS for *Methyl 4-(3-methyl-1H-pyrazol-5-yl)benzoate* **6h**.

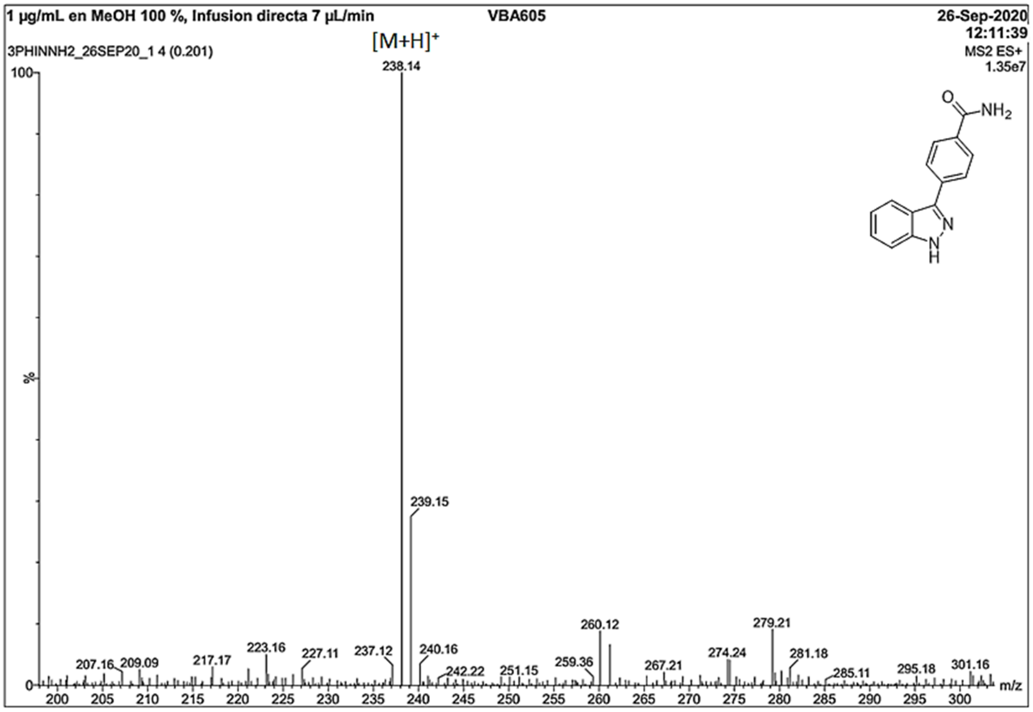

Figure S66. MS for *4-(1H-Indazol-3-yl)benzamide* **10d**.

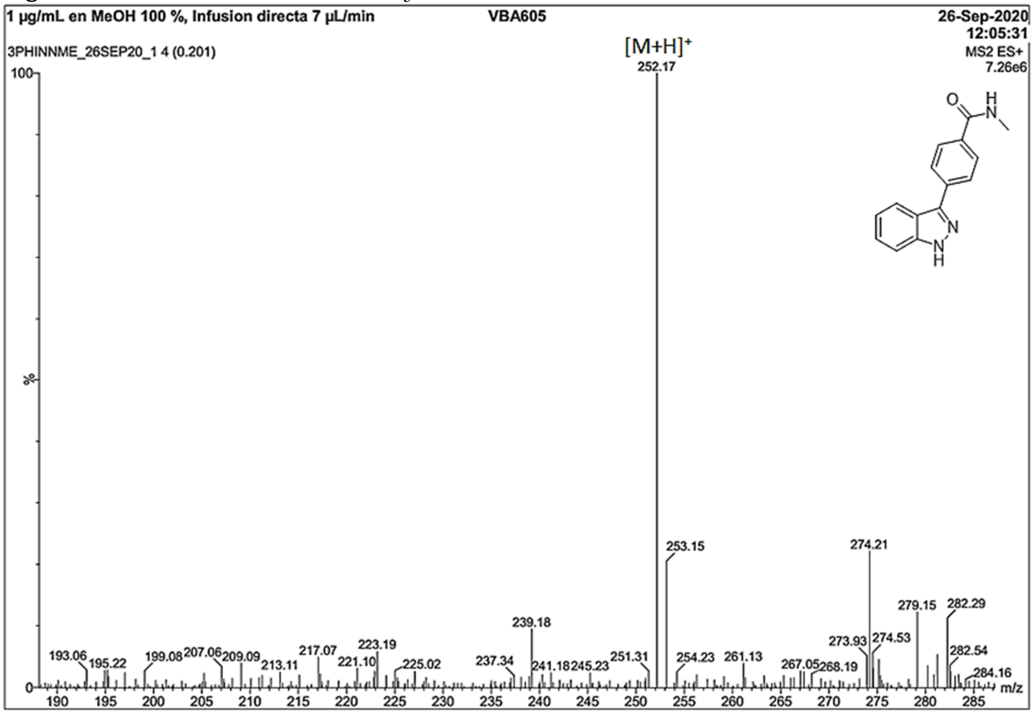

Figure S67. MS for *4-(1H-Indazol-3-yl)-N-methylbenzamide* **10e**.

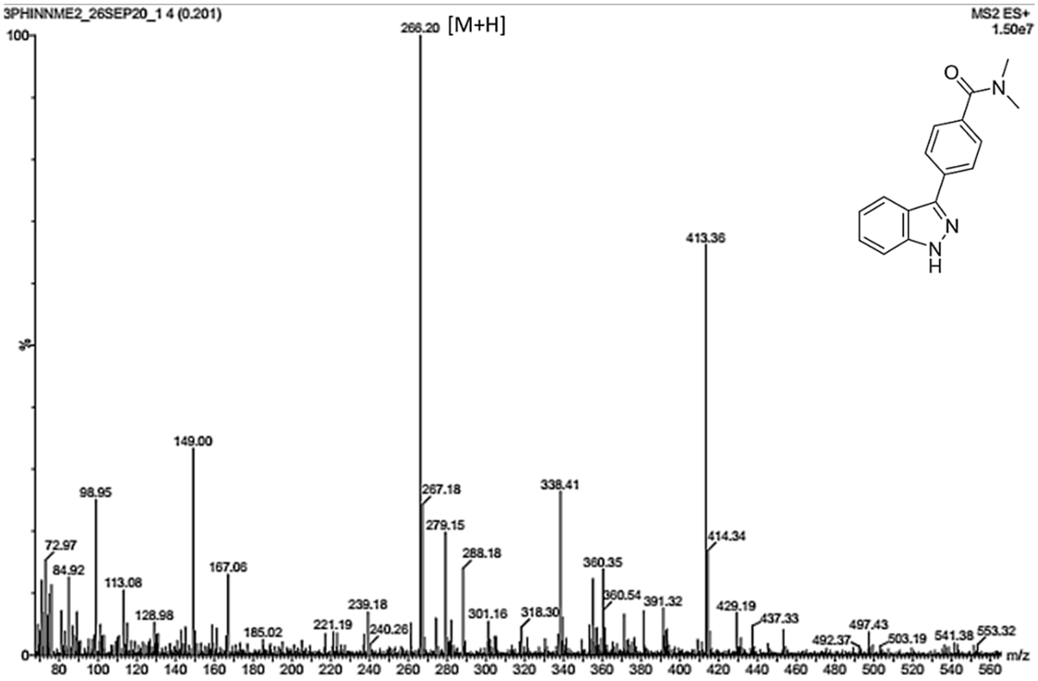

Figure S68. MS for *4-(1H-Indazol-3-yl)-N,N-dimethylbenzamide* **10f**.

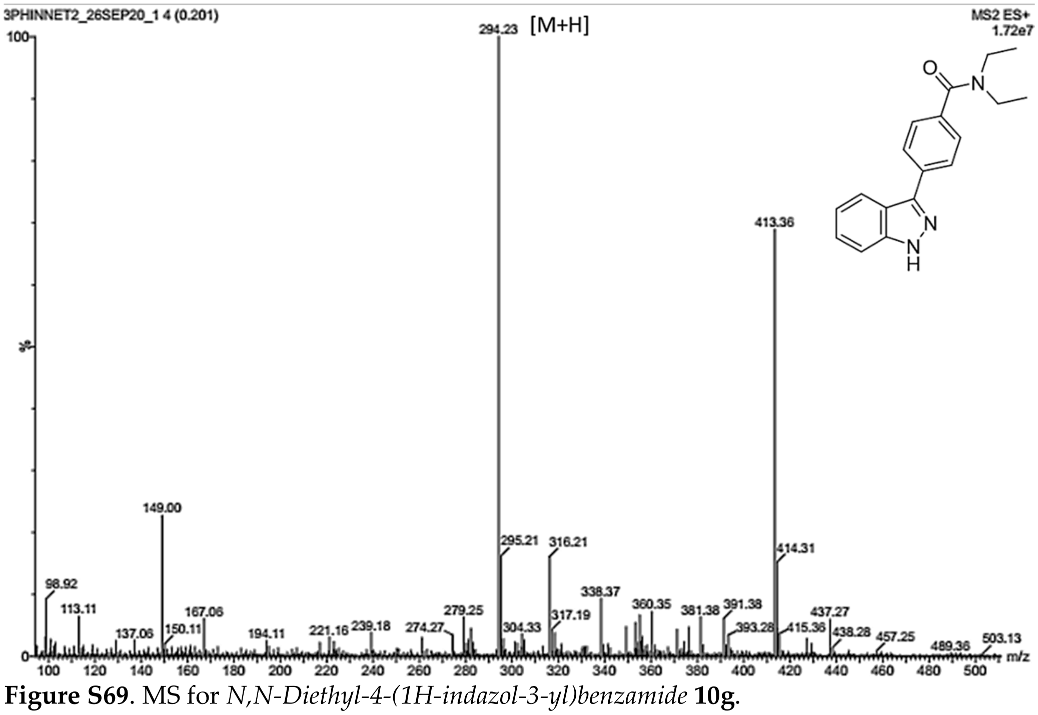

Figure S69. MS for *N,N-Diethyl-4-(1H-indazol-3-yl)benzamide* **10g**.

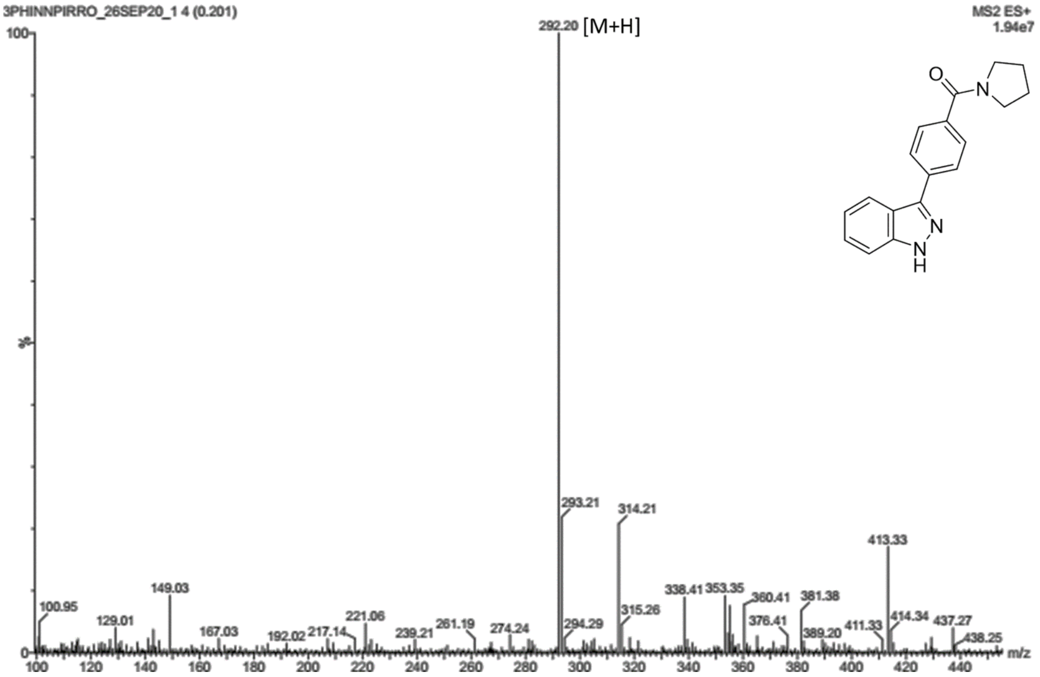

Figure S70. MS for *(4-(1H-Indazol-3-yl)phenyl)(pyrrolidin-1-yl)methanone* **10h**.

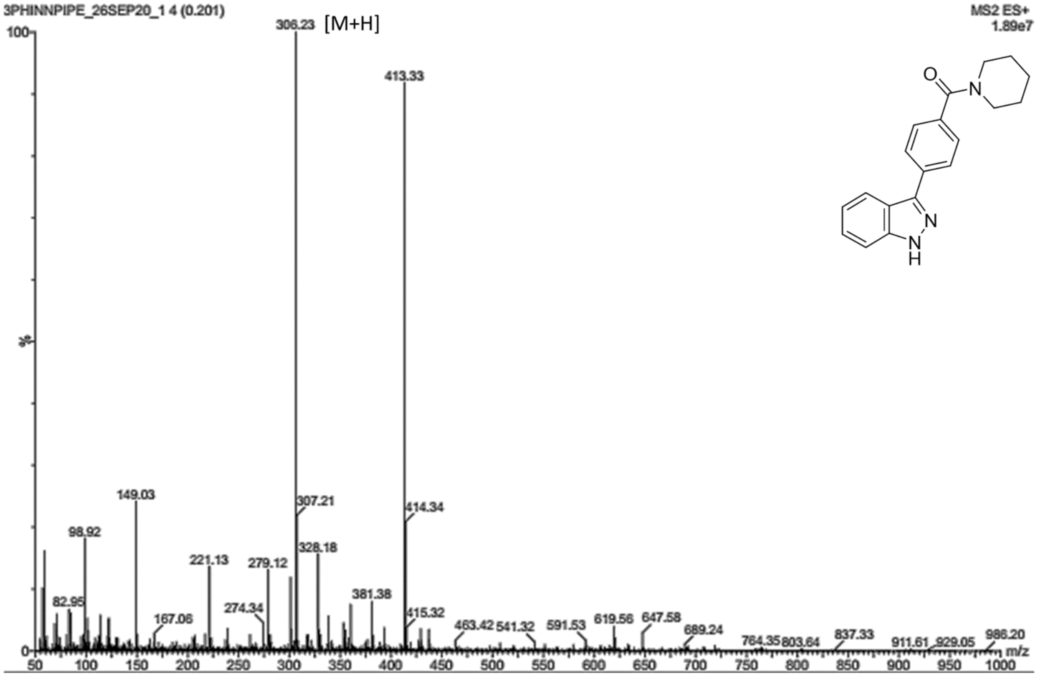

Figure S71. MS for *(4-(1H-indazol-3-yl)phenyl)(piperidin-1-yl)methanone* **10i**.

Table S1. Percentage of purity of compounds **10a**–**i** by qNMR used an internal standard (%purity, mean± standard deviation).

ID	Signal	(S-CH_2-) %	(-CH_2-) %	(-CH_2-Si) %	Mean±SD
**10a**	NH	95.81	97.18	99.62	98.89±2.29
	H5	98.47	99.88	102.38	
**10b**	H6	96.94	96.61	96.94	96.48±0.42
	H5	96.23	95.91	96.23	
**10c**	H6	97.69	96.03	97.95	96.76±1.06
	H5	96.77	95.12	97.02	
**10d**	H6	96.04	94.90	95.94	95.23±0.71
	H7	95.24	94.11	95.14	
**10e**	H6	97.11	95.79	97.44	96.59±0.80
	H5	96.72	95.42	97.06	
**10f**	H6	98.57	98.35	98.67	97.56±1.07
	NH	96.63	96.42	96.73	
**10g**	NH	96.96	96.28	96.86	96.68±0.33
	H6	96.91	96.24	96.81	
**10h**	NH	97.52	99.61	98.09	99.12±1.25
	H5	98.93	101.06	99.51	
	NH		97.26	96.52	
**10i**	H6	*	98.28	97.54	97.32±0.62
	H4+ArH (3H)		97.53	96.8	
**Std**	ArH (4H)	98.65	99.04	98.94	98.88±0.20

* Signal overlapped; Std standard.

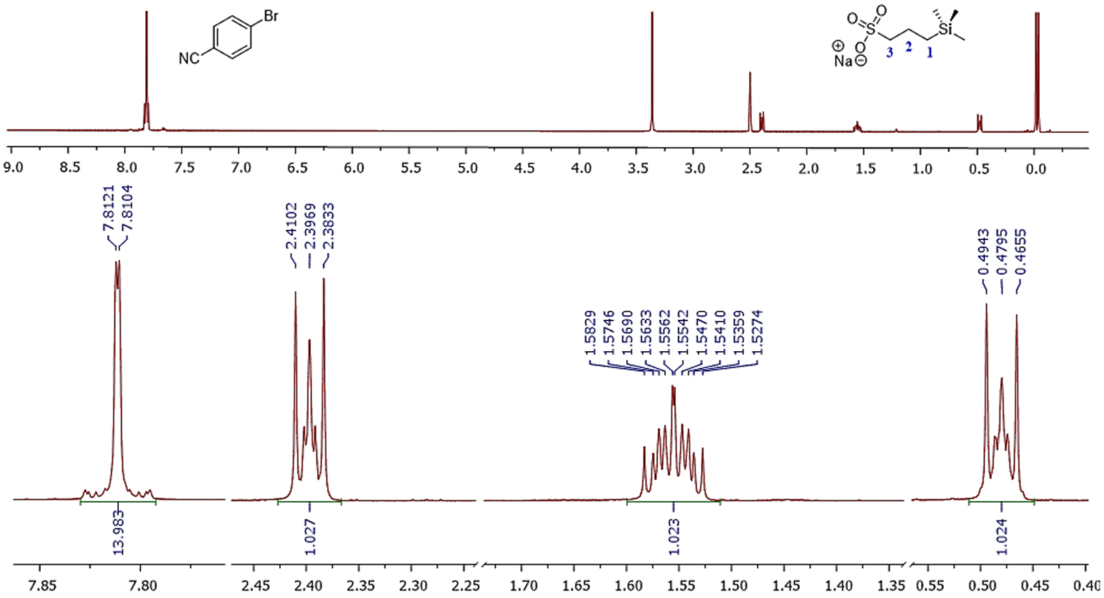

Figure S72. Spectra of qNMR for 4-bromobenzonitrile (purity>99%) as reference compound.
